# Design, Synthesis, and Microbiological Evaluation of Differently Decorated Pyrazoles Able to Overcome Antimicrobial Resistance and Biofilm Formation

**DOI:** 10.1021/acsomega.6c03582

**Published:** 2026-09-22

**Authors:** Matteo Lusardi, Debora Caviglia, Andrea Chiocca, Marco Ponassi, Gabriella Piatti, Annalisa Salis, Andrea Spallarossa, Anna M. Schito, Chiara Brullo

**Affiliations:** † Department of Pharmacy (DIFAR), Section of Medicinal Chemistry, 9302 University of Genoa, Viale Benedetto XV, 3, 16132 Genova, Italy; ‡ Department of Surgical Sciences and Integrated Diagnostics (DISC), University of Genoa, Viale Benedetto XV, 6, 16132 Genova, Italy; § Azienda Ospedaliera Metropolitana Genova, UOS Proteomica e Spettrometria di Massa, L.go. R. Benzi, 10, 16132 Genova, Italy; ∥ Department of Experimental Medicines (DIMES), University of Genoa, Largo Rosanna Benzi 10, PAD.3, 16132 Genova, Italy

## Abstract

Antimicrobial resistance is an urgent global public health threat, killing at least 1.27 million people worldwide and associated with nearly 5 million deaths. Antibiotic resistance has been recently recognized by the WHO as a global health emergency. In this scenario, the research and development of novel derivatives specifically active against resistant pathogens became an urgent need. Pyrazole-based compounds have recently attracted significant interest for their potential antimicrobial activity, particularly against resistant Gram-positive strains such as methicillin-resistant *Staphylococcus aureus* (MRSA). Based on this evidence, we carried out an in-depth investigation of this chemical class by the synthesis of unreported compounds differently decorated on the pyrazole scaffold. The newly prepared derivatives were tested on a large panel of Gram-negative and Gram-positive strains. Interestingly, pyrazolyl ureas (PUs) **1c** and **1h** showed excellent minimum inhibitory concentration (MIC) values (8–16 μg/mL) against different *Staphylococcus* strains and were therefore further evaluated by minimum bactericidal concentration (MBC) determination and time-kill assays and for their ability to inhibit biofilm formation on several MRSA strains. Pyrazole **1c** was shown to exert such inhibition at MIC and sub-MIC concentrations (1/2 MIC and 1/4 MIC), highlighting the pharmaceutical potential of this class of compounds against resistant MRSA. Finally, selected PUs did not affect GM-6114 cell viability, thus indicating the lack of cytotoxicity on healthy fibroblasts, and were predicted to possess favorable physicochemical, pharmacokinetic, and drug-likeness profiles.

## Graphical abstract

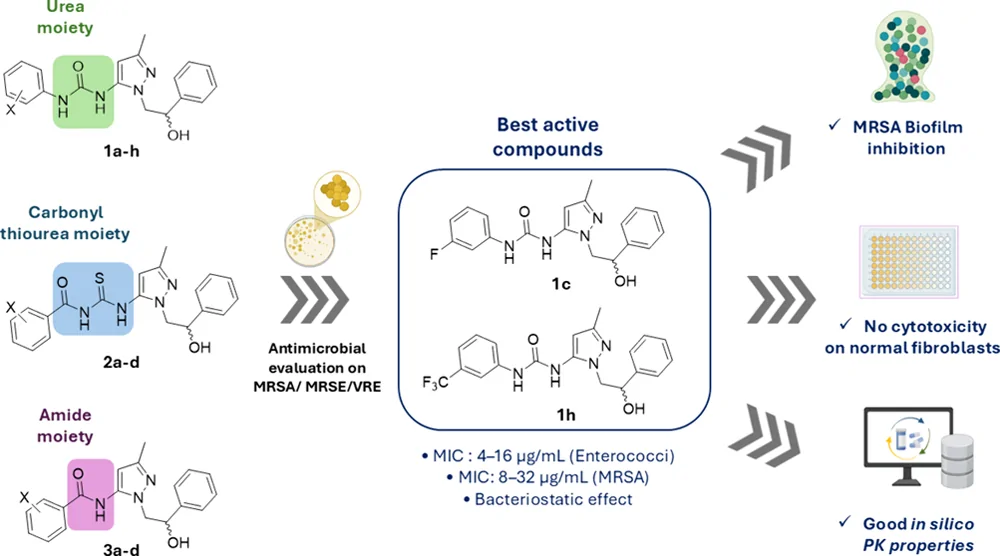

## Introduction

The use of antibiotics has significantly improved human quality of life, revolutionizing the approach to treating and preventing infections and related diseases.^1,2^ Unfortunately, their inappropriate use has led to the development of multidrug resistance (MDR), which is considered one of the most serious health threats of the 21st century.^3–5^ Furthermore, infections are among the most serious complications in cancer patients and antibiotic resistance is also becoming a main issue in cancer treatments.^6^ It is estimated that, by 2050, antibiotic resistance could become the leading cause of death globally if no preventive measures are taken. In 2019, over 1.2 million people died from antimicrobial resistance, and this number is expected to rise to approximately 20 million deaths per year by 2050 if adequate interventions are not adopted to control its spread.^7^

A group of bacteria of particular clinical relevance, frequently isolated in hospital settings and characterized by widespread multidrug resistance, is represented by the acronym ESKAPE, which includes the following microorganisms: *Enterococcus faecium*, *Staphylococcus aureus*, *Klebsiella pneumoniae*, *Acinetobacter baumannii*, *Pseudomonas aeruginosa*, and *Enterobacteriaceae*, that includes *Escherichia coli*, *Klebsiella spp*., *Yersinia spp*. and *Proteus spp*.^8^ *Staphylococcus aureus* is a major human pathogen and a leading cause of hospital-acquired infections, largely due to the emergence of methicillin-resistant strains (methicillin-resistant *Staphylococcus aureus* (MRSA)), which are frequently multidrug-resistant and often exhibit resistance not only to β-lactam antibiotics, but also to several other antibiotic classes, posing a significant challenge to clinical management.^9^

A major factor contributing to antibacterial resistance is the ability of bacteria to form biofilms, highly organized multicellular communities embedded in a self-produced extracellular matrix composed of polysaccharides, proteins, and extracellular DNA. In clinical settings, biofilms protect bacteria from antibiotics, host immune responses, and environmental stresses, promote persistence, and contribute to chronic infections. As a consequence, treatment failures are frequent, particularly in device-associated and hospital-acquired infections.^10,11^

According to the National Institutes of Health (NIH), biofilms may be responsible for up to 80% of human microbial infections, such as meningitis, kidney infections, urinary tract infections, endocarditis, cystic fibrosis, periodontitis, rhinosinusitis, osteomyelitis, and chronic nonhealing wounds, and for a variety of infections related to prosthetic and implantable devices.^12^ As target modification, efflux pump expression, inactivation of the antibiotic molecule, and target bypass, the biofilm formation represents one of the possible forms of bacterial resistance,^13^ allowing bacteria not only to resist antibiotics but also to evade host defense systems.^14^ One of the mechanisms proposed to explain this phenomenon is that the biofilm matrix can act as a physical and chemical barrier, hindering the diffusion of antibiotics and, thereby, limiting their access to metabolically active cells. However, this effect is strongly dependent on the specific antibiotic as some compounds retain the ability to effectively penetrate the biofilm and reach their cellular targets.^15^

Antibiotic penetration into biofilms is strongly influenced by the physicochemical properties of the antibiotic, including molecular size, charge, and hydrophobicity, resulting in variable diffusion through the extracellular matrix and reduced antimicrobial efficacy.^16^ Given the heterogeneous penetration of antibiotics into biofilms and their reduced efficacy against biofilm-embedded bacteria, there is a pressing need to develop new antimicrobial strategies that target biofilm-associated infections.

The search for new compounds, both of natural origin and small molecules capable of fighting MDR bacterial strains such as MRSA, is therefore of great interest. In this context, many pyrazole derivatives have been recently investigated by various research groups for their antimicrobial and antibacterial properties against MRSA strains,^17–25^ and particularly, pyrazole compounds functionalized at position 5 with urea and thiourea functions have been extensively studied.^26,27^ In fact, the urea functionality bears hydrogen bond donor and hydrogen bond acceptor atoms able to interact with the biological target and provide stabilization to the complex. Furthermore, the polar nature of the urea functional group can enhance aqueous solubility.^28–30^

In the past few years, a large library of pyrazolyl ureas (PUs) **I** (Figure 1) characterized by different chains on the pyrazole N1, carboxyethyl or *tert*-butyl as substituents on the C3 or C4 and a urea or thiourea function at position 5, has been synthesized; these compounds showed significant antibacterial activities against antibiotic-resistant Gram-positive strains, in particular *Staphylococcus* strains resistant to methicillin and linezolid (minimum inhibitory concentration (MIC) ranging from 32 and 64 μg/mL).^26,27^ In detail, PU **II** (Figure 1), decorated with a 2-hydroxy-2-phenylethyl chain on N1, a *tert*-butyl function on C-3, and a fluorine atom on the phenyl group of the urea function, resulted the most effective derivatives, being active against selected MDR Gram-positive bacteria, especially against *Staphylococcus* strains (MIC = 32–64 μg/mL) and also moderately active against *Mycobacterium tuberculosis*.^26^ *In silico* studies, time killing tests, and cytotoxicity evaluation confirmed PU **II** as valuable lead compounds for obtaining new antibacterials agents active against Gram-positive strains, including MDR.

**Figure 1:**
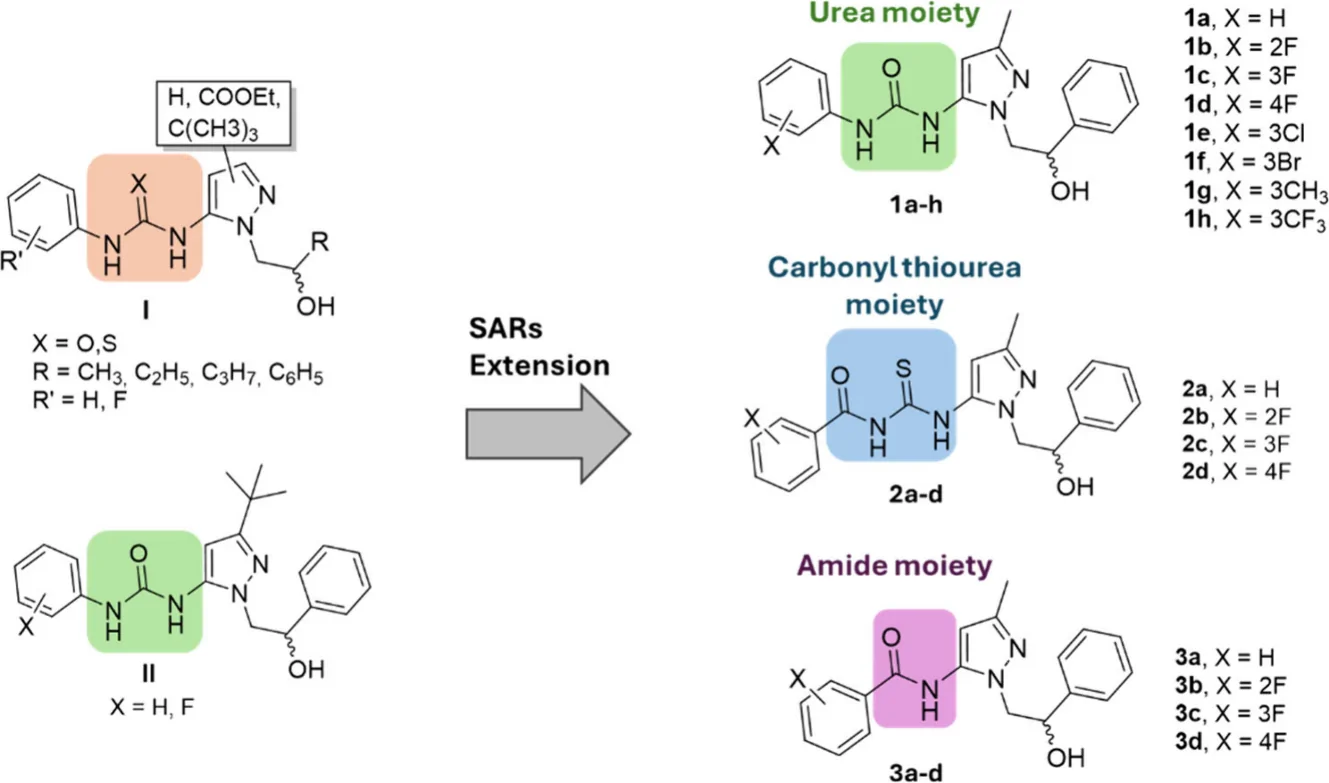
Chemical structures of previous pyrazole compounds **I** and **II** and novel derivatives **1**–**3**.

To further extend the structure–activity relationships (SARs) on PU **II** and identify novel pyrazole compounds with improved antimicrobial activity, compounds **1**–**3** (Figure 1) were designed and synthesized. In particular, the novel compounds share with PUs **II** the N1-pyrazole substituent and are functionalized at the C-5 position with different urea (compounds **1**, Figure 1), thiourea (compounds **2**, Figure 1), or amide (compounds **3**, Figure 1) functions. In all new synthesized derivatives, the *tert*-butyl substituent on C-3 of PU **II** was replaced with a smaller methyl group to investigate whether this reduction in steric hindrance led to enhanced microbiological activity. Based on our previous SAR studies on this class of pyrazoles, which identified the 2-hydroxy-2-phenylethyl group as the most favorable N1 substituent for antimicrobial activity,^26,27^ no further variations at this position were introduced in the present study. This strategy enabled a focused evaluation of the contribution of the C3 and C5 substituents to the antibacterial profile of the pyrazole scaffold.

The new compounds **1**–**3** were preliminarily tested on selected seven different pathogenic strains (four Gram-positive and three Gram-negative species), including MRSA, *methicillin-resistant Staphylococcus epidermidis* (MRSE), vancomycin-resistant *E*. *faecium* (VREF), vancomycin-resistant *Enterococcus faecalis* (VRE), and Gram-negative species (*E*. *coli*, *P*. *aeruginosa*, and *K*. *pneumoniae*, all resistant or multidrug-resistant to current antibacterial drugs). All tested compounds were inactive against Gram-negative strains (MIC > 128 μg/mL), whereas some derivatives **1** (namely, **1c**, **1e**, **1f**, **1h**) showed significant MIC values for the remaining four Gram-positive isolates tested (Table 1). To further characterize the biological profile of the active compounds, 11 additional strains, belonging to the species found to be susceptible in the initial screening, were added and analyzed. Table 1 shows the MIC values of compounds **1c**, **1e**, **1f**, and **1h** against a total of 15 strains belonging to Gram-positive species of *S*. *aureus*, *S*. *epidermidis*, *E*. *faecalis*, and *E*. *faecium* strains.

**Table 1:** MIC values of compounds **1c**, **1e**, **1f**, and **1h** against *S*. *aureus*, *S*. *epidermidis*, *E*. *faecalis*, and *E*. *faecium* Strains and Those of Reference Antibiotics^a^

Strains Used	**1c** (μg/mL)	**1e** (μg/mL)	**1f** (μg/mL)	**1h** (μg/mL)	Reference Compound (μg/mL)
*S*. *aureus* B^b^	16	16	32	8	512^(O)^
*S*. *aureus* 18^b^	32	16	32	8	512^(O)^
*S*. *aureus* 187^b^	16	32	64	8	512^(O)^
*S*. *aureus* 189^b^	32	16	32	16	128^(O)^
*S*. *epidermidis* 22^b^	32	32	64	8	256^(O)^
*S*. *epidermidis* 178^b^ ^,^ ^c^	64	64	64	16	256^(O)^
*S*. *epidermidis* 180^b^ ^,^ ^c^	32	64	64	16	128^(O)^
*S*. *epidermidis* 181^b^ ^,^ ^c^	32	32	64	16	256^(O)^
*E*. *faecalis* 1a^d^	8	16	16	8	256^(V)^, 64^(T)^
*E*. *faecalis* 431^e^	8	32	32	8	32^(V)^
*E*. *faecalis* 439^d^	8	32	32	8	256^(V)^, 64^(T)^
*E*. *faecalis* 365^e^	16	16	16	16	32^(V)^
*E*. *faecium* 479^d^	16	64	64	8	128^(V)^, 64^(T)^
*E*. *faecium* 179^d^	8	64	64	4	128^(V)^, 64^(T)^
*E*. *faecium* 185^d^	16	32	64	8	128^(V)^, 64^(T)^

a Only compounds with MIC values < 128 μg/mL are reported. Experiments were carried out in triplicate. Reference antibiotics were oxacillin (O), vancomycin (V), and teicoplanin (T). Gram-negative strains, for whom MIC values were greater than 128 μg/mL, were not reported.

b Indicates resistance toward methicillin.

c Indicates resistance to linezolid.

d Indicates resistance to vancomycin and teicoplanin.

e Indicates resistance to vancomycin.

Compounds **1c** and **1h** showed antibacterial properties against both *Staphylococcus* and *Enterococcus* species and were further studied through: i) additional microbiological evaluation against ten *S*. *aureus* and *S*. *epidermidis* strains resistant to methicillin and/or linezolid and endowed with biofilm-forming capacity (Table 2); ii) time killing experiments on *S*. *aureus* strains (Figure 2) and the minimum bactericidal concentration (MBC) determination against selected *S. aureus* strains (18, 187 and A) (Table 3) to verify if compounds are bacteriostatic or bactericidal, iii) inhibition of biofilm formation (Figure 3, Table 4, Figure S35, Table S1 and Table S2), and iv) cytotoxicity on healthy GM-6114 embryonic human fibroblasts (Table 5). Finally, the *in silico* pharmaceutical relevance of compounds **1c** and **1h** and their pharmacokinetics properties, as well as their drug-likeness, were calculated using SwissADME (Table S2).^31^

**Table 2:** MIC Values of Compounds **1c** and **1h** and Oxacillin (Reference Compound) against Other*S*. *aureus* and *S*. *epidermidis* Strains^a^

Strains	**1c** (μg/mL)	**1h** (μg/mL)	Oxacillin (μg/mL)
*S*. *aureus* A^b^	16	8	512
*S*. *aureus* C^b^	16	8	256
*S*. *aureus* D^b^	16	8	256
*S*. *aureus* 10^b^	32	16	256
*S*. *aureus* 11^b^	32	16	256
*S*. *aureus* 24^b^	32	16	128
*S*. *aureus* ATCC 29213	32	8	0.5
*S*. *aureus* N_2_	32	16	1
*S*. *epidermidis* 2^b^	32	16	128
*S*. *epidermidis* 198^b^ ^c^	32	8	256

a Experiments were carried out in triplicate.

b Indicates resistance toward methicillin.

c Indicates resistance to linezolid.

**Table 3:** MBCs for Compounds **1c** and **1h**

	MBC (μg/mL)	
Strain	**1c**	**1h**
*S*. *aureus* 18	>128	>128
*S*. *aureus* 187	>128	>128
*S*. *aureus* A	>128	>128

**Figure 2:**
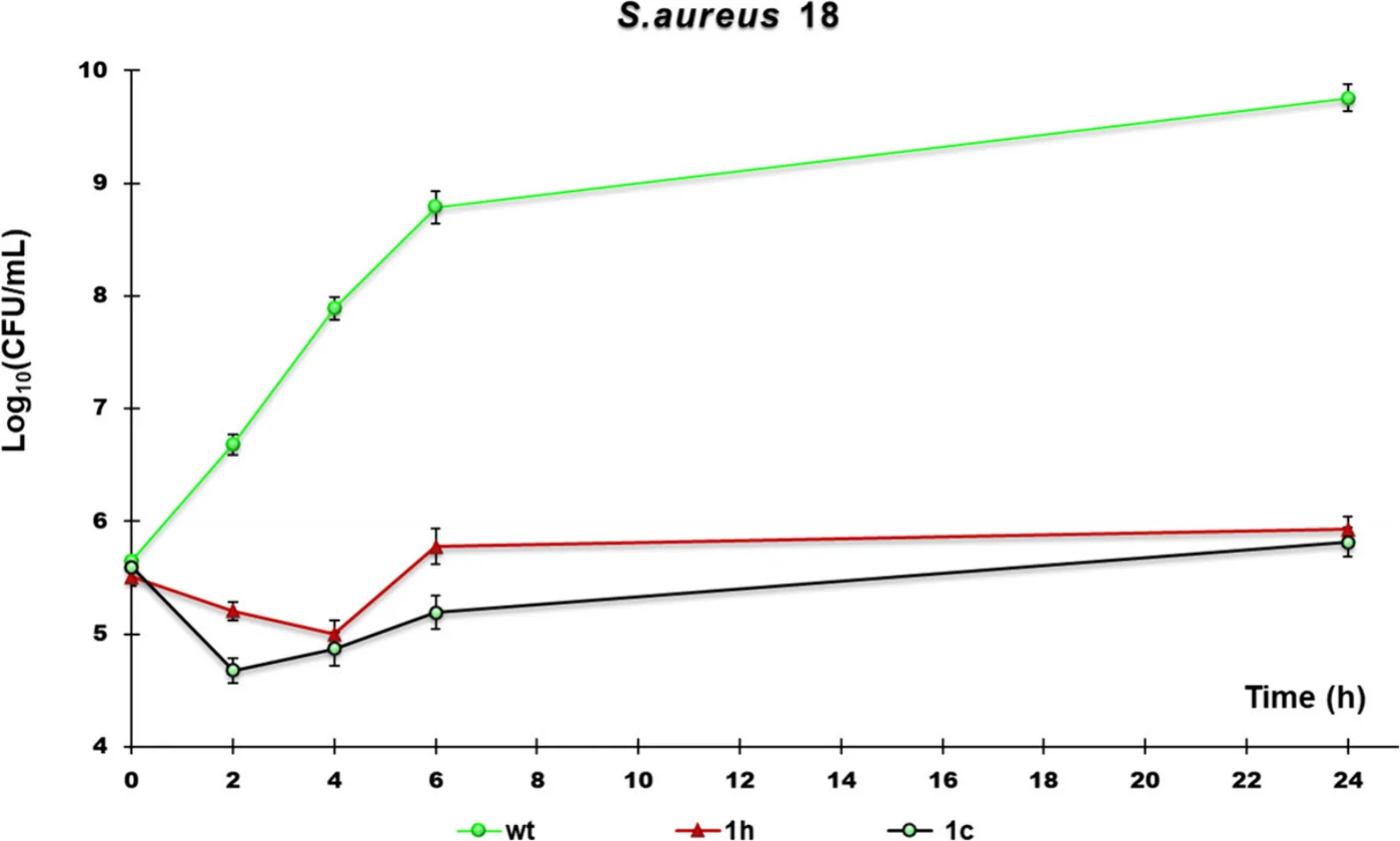
Time-killing curves for compounds **1c** (in black) and **1h** (in red) at 4× MIC concentration on *S*. *aureus* 18. Untreated control strain was run in parallel (**wt** in green).

**Figure 3:**
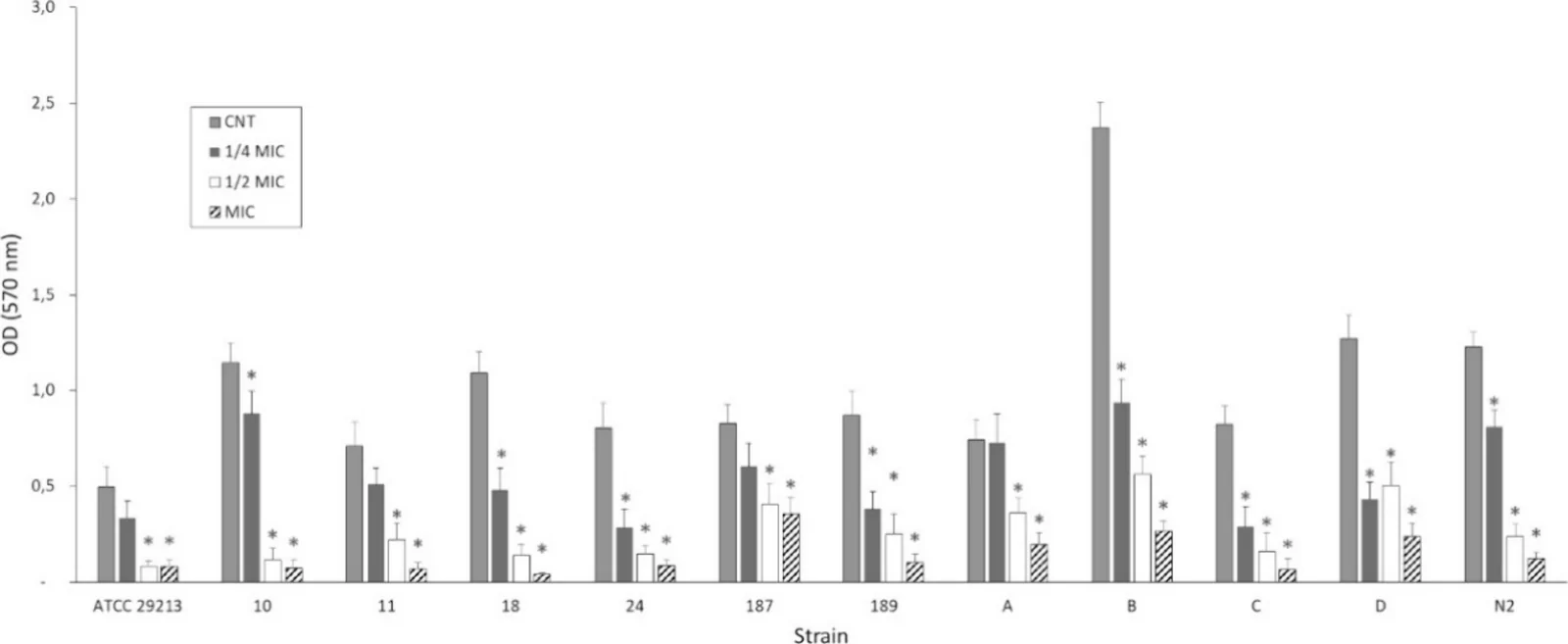
Effect of **1c** on bacterial growth of different *S*. *aureus* strains. Bacterial growth was quantified by measuring optical density at 570 nm (OD_570_) after 24 h of incubation. The reference strain *S*. *aureus* ATCC 29213 and clinical isolates (10, 11, 18, 24, 187, 189, A, B, C, D, and N2) were treated with compound **1c** at sub-inhibitory concentrations (1/4 MIC and 1/2 MIC) and at the minimum inhibitory concentration (MIC) and compared with untreated controls (CNT). Data are presented as mean ± standard deviation of at least three independent experiments. Statistical analysis was performed using one-way ANOVA test; asterisks indicate statistically significant differences compared to the corresponding control (Dunnett test, *p* < 0.0001).

**Table 4:** Inhibition Percentage of Biofilm Formation (±SD) of **1c** at Different Concentrations (1/4 MIC, 1/2 MIC, and MIC)^a^

	% Inhibition of Biofilm Formation		
Strain	1/4 MIC	1/2 MIC	MIC
*S*. *aureus* ATCC 29213	32 ± 8	83 ± 6	83 ± 1
*S*. *aureus* 10*	23 ± 3	90 ± 4	93 ± 2
*S*. *aureus* 11*	28 ± 8	69 ± 11	90 ± 2
*S*. *aureus* 18*	56 ± 5	87 ± 5	96 ± 1
*S*. *aureus* 187*	27 ± 14	51 ± 13	57 ± 8
*S*. *aureus* 189*	56 ± 6	71 ± 13	88 ± 3
*S*. *aureus* 24*	65 ± 10	82 ± 5	89 ± 4
*S*. *aureus* A*	12 ± 3	52 ± 11	74 ± 7
*S*. *aureus* B*	60 ± 4	76 ± 4	89 ± 1
*S*. *aureus* C*	65 ± 11	80 ± 7	92 ± 1
*S*. *aureus* D*	66 ± 3	60 ± 7	81 ± 5
*S*. *aureus* N2*	34 ± 1	81 ± 5	90 ± 1

a Experiments were carried out in triplicate; * = methicillin-resistant.

## Results and Discussion

Compounds **1**–**3** were synthesized as racemic mixtures through the two-step, divergent approach shown in Scheme 1. In detail, 2-hydrazino-1-phenylethanol **4** ^32^ was condensed with 3-aminocrotonitrile in absolute ethanol in the presence of 1 M hydrochloric acid under reflux for 5 h to give the desired 2-(5-amino-3-methyl-1*H*-pyrazol-1-yl)-1-phenylethan-1-ol **5**, which represents the key intermediate for the synthesis of all the final compounds. In detail, the reaction of **5** with properly substituted phenyl isocyanates in anhydrous toluene under reflux led to the isolation of PUs **1a**–**h** in good yield (25–58%). Alternatively, thiourea derivatives **2a**–**d** were prepared through the condensation of **5** and suitable benzoylisothiocyanates^33^ in anhydrous tetrahydrofuran (THF) at reflux for 12 h. Finally, the acylation of amino-pyrazole **5** with commercially available benzoyl chlorides in the presence of TEA in THF at reflux for 18 h afforded pyrazolyl amides **3a**–**d**.

**Scheme 1:**
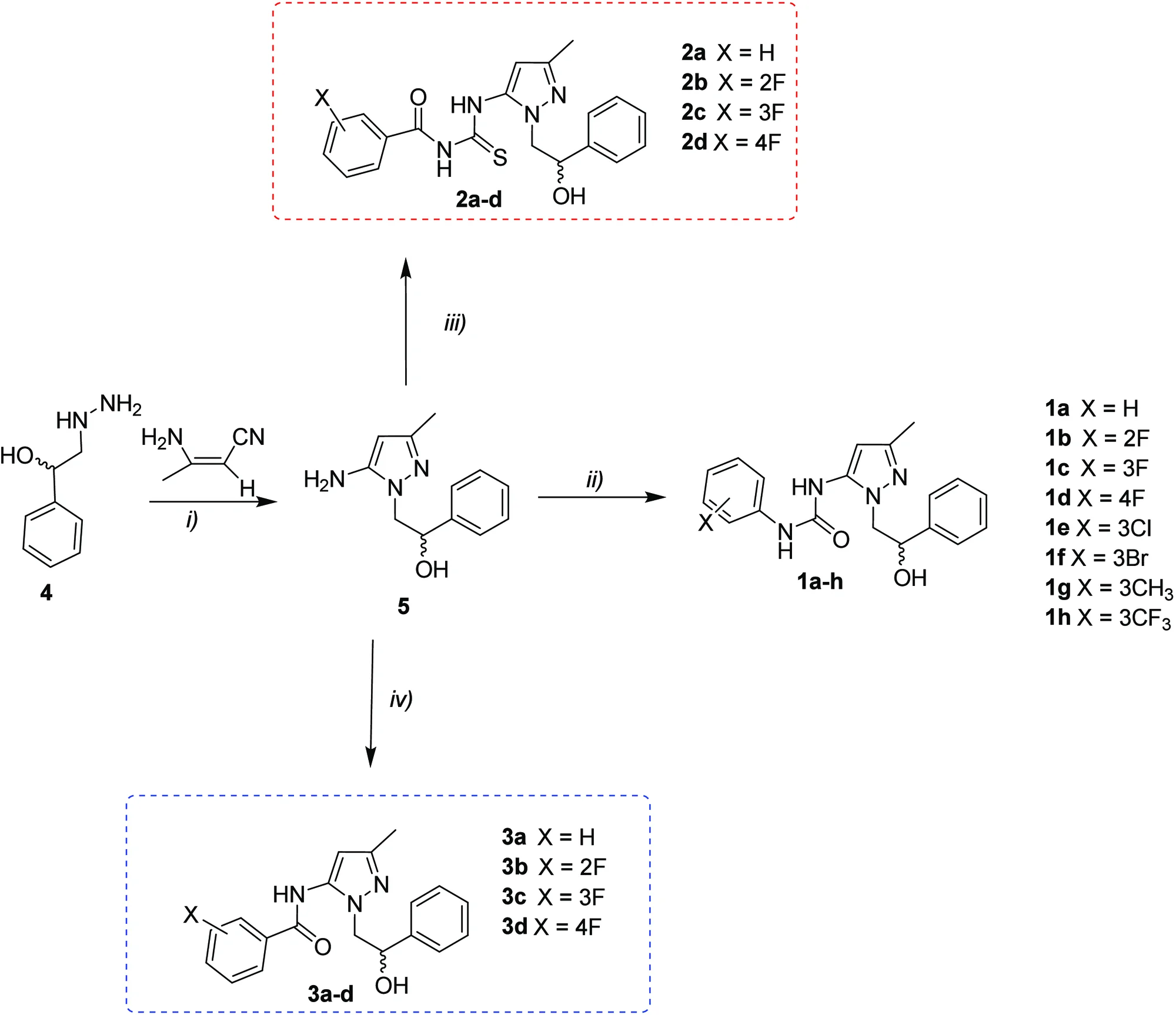
Synthesis of New Pyrazoles **1**–**3** ^a^

a Reagents and conditions: (i) 3-aminocrotonitrile, 1 M HCl, reflux, 5 h; (ii) proper phenyl isocyanate, an. Toluene, reflux, 6 h; (iii) proper benzoyl isothiocyanate, an. THF, reflux, 12 h; (iv) TEA, an. THF, 0 °C and then proper benzoyl chloride, reflux 18 h.

All synthesized compounds were fully characterized by ^1^H NMR and ^13^C NMR. In detail, the disappearance of the broad singlet at 4.95 ppm in the ^1^H NMR spectrum of **5** confirmed the functionalization of its primary amino group. The ^1^H NMR and ^13^C NMR spectra of the final compounds presented diagnostic peaks for the urea (compounds **1**), acylthiourea (compounds **2**), and amide (compounds **3**) functionalities (see Supporting Information). Elemental analysis confirmed the compounds’ identities and purities (>95%). In addition, HRMS analyses were carried out for representative derivatives **1c**, **1h**, **2a**, and **3c** to provide additional structural confirmation, whereas analytical HPLC was performed for the two most active compounds (**1c** and **1h**; *vide infra*).

The antibacterial activity of compounds **1**–**3** was preliminarily evaluated by determining their MIC values against seven pathogenic isolates, including four Grampositive (*S*. *aureus*, *S*. *epidermidis*, *E*. *faecalis*, and *E*. *faecium*) and three Gramnegative (*E*. *coli*, *P*. *aeruginosa*, and *K*. *pneumoniae*) strains. All isolates were MDR to currently used antibiotics and representative of the most common bacterial species encountered in clinical settings. As the previous PU **II**, all tested compounds were inactive against the Gram-negative strains (MIC > 128 μg/mL), whereas variable antibacterial activity was observed against Gram-positive isolates (Table 1). In particular, derivatives **1c**, **1e**, **1f**, and **1h** proved to be active against all considered Gram-positive species showing MIC value in the 8–64 μg/mL range (Table 1). These data highlighted the essential role of the urea functionality to define antimicrobial activity (in fact, acyl thiourea **2** and amide derivatives **3** proved to be inactive) and point at the *meta* substitution of the urea phenyl ring with an electron withdrawing group (i.e., F, Cl, Br, and CF_3_) as a key determinant for activity.

Compounds **1c**, **1e**, **1f**, and **1h** were further tested on an expanded panel of strains belonging to the species identified as being susceptible in the preliminary screening. As reported in Table 1, the selected derivatives showed a widespread activity against 15 clinical Gram-positive strains that included four isolates of *S*. *aureus* resistant to methicillin (MRSA), four *S*. *epidermidis* (one of which was resistant to methicillin (MRSE) and three, resistant to methicillin and linezolid), four *E*. *faecalis*, and three *E*. *faecium* Strains, variously resistant to vancomycin and teicoplanin. Noteworthily, the novel pyrazolyl ureas proved to be as active as or more potent than clinically used antibiotics (i.e., oxacillin, vancomycin, and teicoplanin).

In particular, the fluorine-containing compounds **1c** (X = 3F) and **1h** (X = 3CF_3_) exhibited higher antimicrobial potency than their chlorinated or brominated analogues **1e** and **1f**. Specifically, compound **1c** showed MIC values ranging from 16 to 64 μg/mL against staphylococci and from 8 to 16 μg/mL against enterococci, whereas compound **1h** displayed MIC values between 8 and 16 μg/mL against staphylococci and between 4 and 16 μg/mL against enterococci. To consolidate these findings and further explore the antimicrobial profile of these promising hits, PUs **1c** and **1h** were evaluated against ten additional *S*. *aureus* and *S*. *epidermidis* strains resistant to methicillin and/or linezolid and endowed with biofilm-forming capacity (Table 2).

Both derivatives showed good activity against numerous MRSA clinical isolates with overall MIC values ranging from 8 to 32 μg/mL against multiple MRSA clinical isolates. Compound **1h** demonstrated greater potency than its analogue **1c**, reaching MIC values of 8 μg/mL against numerous strains. The results were also satisfactory against the various MRSE strains. Thus, **1h** maintained consistent activities with MIC values between 8 and 16 μg/mL, while **1c** showed MIC values in the range of 16–32 μg/mL. Overall, among the staphylococcal species analyzed, **1h** confirmed its potent and widespread antibacterial profile against a number of drug-resistant strains.

Given their potent antibacterial activity, the most active compounds **1c** and **1h** were further investigated to better characterize their mode of action. Initially, the minimum bactericidal concentration (MBC) was determined against selected *S*. *aureus* strains (18, 187, and A). As reported in Table 3, no compound achieved the ≥99.9% reduction in viable bacterial counts required to define bactericidal activity within the concentration range tested, suggesting a predominantly bacteriostatic effect.

To further verify this behavior and assess the kinetics of bacterial inhibition over time, time-kill assays were subsequently performed on the same strains at 4× MIC (Figure 2). The time-kill experiments confirmed the bacteriostatic profile suggested by the MBC results; in detail, time-kill assays were performed against three *S*. *aureus* strains (18, 187, and A). This analysis is generally considered a more informative approach for assessing the kinetics of bacterial killing and distinguishing bactericidal from bacteriostatic effects. In fact, time-kill experiments, unlike end point MBC measurements, provide dynamic information on the rate, extent, and persistence of bacterial killing over time, thus allowing a more accurate preliminary pharmacodynamic characterization of the tested compounds. The experiments were carried out at 4× MIC concentrations (Figure 2). As exemplified for the MRSA strain 18 (Figure 2), the two compounds exerted a bacteriostatic effect. The same trends were observed also for *S*. *aureus* strains 187 and A. Specifically, compared with the untreated control, cultures treated with compounds **1c** and **1h** exhibited a slight reduction in bacterial growth during the initial hours of incubation. Subsequently, bacterial counts remained stable up to 24 h and comparable to the initial inoculum, supporting a bacteriostatic mode of action for both derivatives.

The inhibitory activities of the active pyrazole derivatives **1c** and **1h** against biofilm formation were investigated. *S*. *aureus* (strains ATCC 29213, 10, 11, 18, 24, 187, 189, A, B, C, D, and N2, Figure 3) and *S*. *epidermidis* (strains 2, 22, and 198) represent two pathogens posing a serious public health challenge worldwide^34,35^ and were grown in triplicate under static conditions for 24 h in 96-well polystyrene plates and simultaneously exposed to the compounds at different concentrations (1/4 MIC, 1/2 MIC, and MIC). Compound **1h** did not exhibit significant inhibition of biofilm formation in the two species (data not shown), whereas its close analogue **1c** was inactive on *S*. *epidermidis* (data not shown) but endowed with a marked inhibitory effect on *S*. *aureus* biofilm formation.

The antibiofilm activity of compound **1c** against 12 *S*. *aureus* strains (ATCC 29213, 10, 11, 18, 24, 187, 189, A, B, C, D, and N2) is summarized in Figure 3 and Table 4. Treatment with compound **1c** resulted in a concentration-dependent reduction of biofilm biomass in the majority of the tested strains, as assessed by OD_570_ measurements (Figure 3, Table 4), including both the reference strain ATCC 29213 and clinical isolates. At subinhibitory concentrations (1/4 MIC), biofilm inhibition was already detectable in most strains, although with marked strain-to-strain variability, ranging from minimal effects (e.g., strain A) to reductions exceeding 60% in several isolates. Increasing the concentration to 1/2 MIC resulted in a pronounced decrease in biofilm biomass with inhibition values generally ranging from approximately 50% to 90%. At the MIC, compound **1c** exhibited a strong antibiofilm effect against all strains, leading to biofilm reductions typically above 80% and reaching up to 95% in certain isolates (e.g., strain 18).

The selective antibiofilm activity observed for compound **1c** against *S*. *aureus*, but not *S*. *epidermidis*, is likely related to species-specific differences in biofilm regulatory networks and extracellular matrix composition.^36^ Such differences, which reflect distinct mechanisms of biofilm development in the two species, highlight the complexity of staphylococcal biofilms and merit further investigation to clarify the molecular targets underlying the antibiofilm effects of compound **1c**.

The antibiofilm activity of compound **1c** was compared to that of vancomycin against the same strains (Figure S35, Table S1 and Table S2). While vancomycin significantly reduced planktonic growth, its ability to inhibit biofilm formation was limited and highly variable among strains, particularly at subinhibitory concentrations. Under those conditions (1/4 MIC and 1/2 MIC), most strains showed little or no reduction in biofilm biomass and, in some cases, even increased biofilm formation relative to the untreated control. Increasing the concentration to MIC did not result in a consistent antibiofilm effect across the strains. Overall, these data indicate that vancomycin lacks a clear concentration-dependent antibiofilm activity, in agreement with previous reports, describing its limited efficacy against biofilm-associated *S*. *aureus* cells.^37,38^

The cytotoxicity of the most active PUs **1c** and **1h** was evaluated against embryonic normal human fibroblast GM-6114 at 4× MIC concentrations, according to the ECVAM guidelines.^39,40^ As reported in Table 5, the two compounds did not affect human healthy fibroblast GM-6114 cell viability, thus indicating the lack of cytotoxicity on healthy fibroblasts and a favorable selectivity profile toward bacterial cells over normal human cells under the tested conditions.

**Table 5:** Cell Growth Percentage Values of Compounds **1c** and **1h** on Human Embryonic Fibroblasts (GM-6114) at 100 μM Concentration^a^

	Cell Growth Percent (%)^b^	
Cell Line	**1c**	**1h**
Human embryonic fibroblasts GM-6114	53	69

a Data are mean values for three separate experiments.

b Variation among triplicate samples was less than 10%.

To further characterize their pharmaceutical potential, the pharmacokinetics and drug-likeness properties of **1c** and **1h** were calculated by SwissADME (Table S3).^31^ As detailed in Table S3, the two derivatives displayed a comparable number of rotatable bonds (seven and eight, respectively), a favorable range for balancing conformational flexibility leading to improved affinity and selectivity. Compounds **1c** and **1h** present four and six hydrogen acceptor bonds, respectively, while both molecules possess two hydrogen bond donors. These interactions would be important for specific target binding and may affect compound solubility and absorption.^41^ PU **1h** displays a higher logP value (3.10, Table S3) than **1c** (2.32, Table S3), consistent with the presence of a trifluoromethyl group on the phenyl ring that increases molecule hydrophobicity. This characteristic would be associated with lower aqueous solubility for **1h** (2.55 × 10^–3^ mg/mL, Table S3) compared with **1c** (1.08 × 10^–2^ mg/mL, Table S3), suggesting less efficient absorption under physiological conditions.^42^ Interestingly, both compounds are predicted to hold high gastrointestinal absorption and good blood–brain barrier (BBB) permeability. However, they seemed to be P-gp substrates, indicating possible active efflux that could limit intracellular bioavailability. Derivatives **1c** and **1h** share a similar cytochrome P450 inhibition profile, being predicted CYP2C19, CYP2D6, and CYP3A4 inhibitors. Notably, no violations of Lipinski’s rules are observed, supporting good compatibility with the physicochemical requirements of orally bioavailable bioactive molecules. Overall, pyrazole **1c** appears to possess a slightly more favorable profile in terms of solubility and pharmacokinetic properties than **1h**, whereas the latter would better interact with cell membranes and hydrophobic targets due to its higher lipophilicity.

## Conclusions

The results obtained further expand the SARs of PU **II** derivatives and confirm that optimal antimicrobial activity is achieved through a precise functionalization of the pyrazole core, consisting of a urea moiety at position 5, a small methyl group at C-3, and a hydrogen atom at C-4, as summarized in Figure 4. Within this framework, compounds **1c**, **1e**, **1f**, and **1h**, bearing a halogen substituent at the *meta* position of the phenyl ring of the urea moiety, exhibited broad-spectrum activity against drug-resistant Gram-positive pathogens.

**Figure 4:**
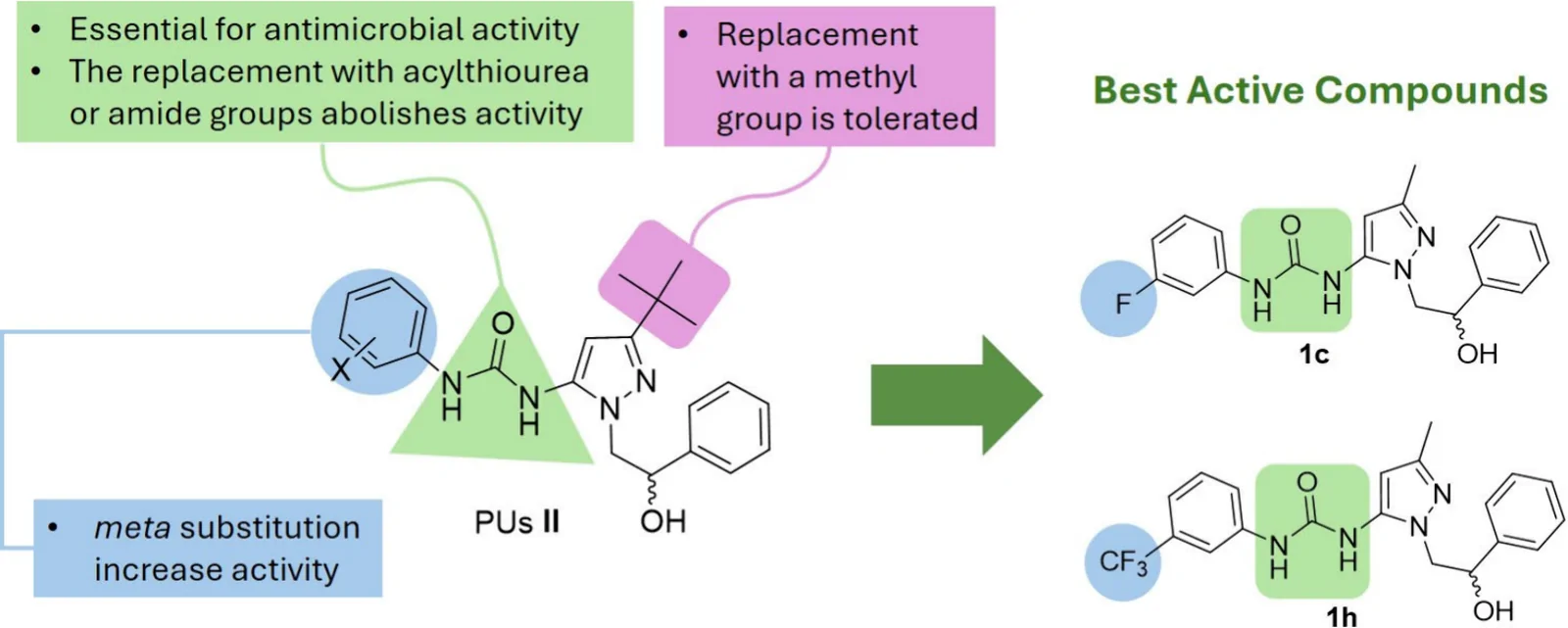
SARs emerging from the present study and chemical structures of the most active PUs of the series.

In particular, compound **1c** emerged as the most promising derivative, displaying consistent and potent activity against the tested Gram-positive strains, as reflected by the reproducible MIC values. Furthermore, **1c** inhibited the biofilm formation by *S*. *aureus*, including multidrug-resistant isolates, which is a pathogen of major clinical concern. Notably, the antibiofilm activity of compound **1c** was evident even at sub-MIC concentrations, suggesting that the compound may interfere with early stages of biofilm development or with regulatory pathways governing biofilm formation rather than solely relying on growth inhibition.

In addition, compound **1c** showed no detectable cytotoxicity in MTT assays, even at high concentrations (100 μM), and was predicted to possess favorable physicochemical, pharmacokinetic, and drug-likeness profiles.

The activity displayed by some of the newly synthesized pyrazoles (namely, **1c**, **1e**, **1f**, and **1h**) against several multidrug-resistant Gram-positive bacterial strains suggests that their mechanism of action is likely distinct from that of β-lactam antibiotics to which most of the tested strains are resistant. In particular, for the most active derivative **1c**, the bacteriostatic profile together with the inhibition of biofilm formation at sub-MIC concentrations suggests that this pyrazolyl urea may interfere with bacterial adaptive responses and biofilm regulatory systems rather than exert a direct bactericidal effect. These findings suggest that the compound primarily interferes with the early stages of biofilm development rather than with the eradication of an established biofilm. However, the molecular target and the mechanism underlying this effect remain unknown and will be investigated in future studies. Nevertheless, compound **1c** represents a promising new lead scaffold for the development of a novel antibacterial chemotype with potential utility against difficult-to-treat bacterial infections.

## Experimental Section

### Chemistry Section

#### General Information

All chemicals were purchased from Chiminord srl and Merck (formerly Aldrich Chemical) (Milan, Italy). Solvents were reagent-grade. All commercial reagents were used without further purification. Aluminum-backed silica gel plates (Merck DC-Alufolien Kieselgel 60 F254, Darmstadt, Germany) were used in thin-layer chromatography (TLC) for routine monitoring of reaction progress. Merck silica gel, 230–400 mesh, was used for chromatography. Flash chromatography was performed using an Isolera One Instrument (Biotage, Uppsala, Sweden) using a Silica gel column. Melting points were measured with a Buchi M-560 instrument (Buchi instruments, Flawil, Switzerland). ^1^H and ^13^C NMR spectra were recorded on a JEOL JNM ECZ-400S/L1 instrument (400 MHz, Tokyo, Japan); chemical shifts are reported as δ (ppm) relative to tetramethylsilane (TMS) as internal standard, and signals were characterized as s (singlet), d (doublet), t (triplet), q (quartet), m (multiplet), or bs (broad signal). *J* values were reported in Hz. IR spectra were recorded on a PerkinElmer 398 instrument. The HRMS experiments were performed using a Q Exactive Orbitrap instrument by Thermo Scientific (Waltham, MA, USA). The analytical instrument was an Agilent 1260 high-performance liquid chromatograph (HPLC, Agilent Technologies, Santa Clara, CA, USA). The analytical HPLC column was a Phenomenex column (Torrance, CA, USA) C18 Luna (4.6 × 250 mm, 5 μm). Compound purity was determined by integrating peak areas of the chromatogram obtained in the liquid phase, monitored at 254 nm. Elemental analysis was determined with an elemental analyzer EA 1110 (Fison Instruments, Milan, Italy); products were considered pure when the difference between calculated and found values was ±0.4.

##### Synthesis of 2-(5-Amino-3-methyl-1*H*-pyrazol-1-yl)-1-phenylethanol (**5**)

A solution of 1-phenyl-2-hydrazinoethanol **4** (1.50 g, 10 mmol) and 3-aminocrotonitrile (0.90 g, 11 mmol) in 1 M hydrochloric acid (20 mL) was heated to reflux for 5 h. After cooling to room temperature, the reaction mixture was basified with 1 M sodium hydroxide. The obtained solid was filtered, washed with water, and recrystallized from absolute ethanol to afford the desired compound **5** as a pure, white solid.

##### General Synthesis of Pyrazolyl Ureas (PUs) (**1a**–**h**)

To a solution of 2-(5-amino-3-methyl-1*H*-pyrazol-1-yl)-1-phenylethanol **5** (217 mg, 1 mmol) in anhydrous toluene (10 mL), the appropriate phenyl isocyanate (1.1 mmol) was added dropwise, and the reaction mixture was heated at reflux for 6 h. The white solid obtained was filtered and purified by flash or column chromatography on silica gel, eluting first with DCM and then with a mixture of diethyl ether/methanol (9/1). The product was then recrystallized from diethyl ether/petroleum ether (bp 50–60 °C) 1/1 to afford white solids.

##### General Synthesis of Pyrazolyl Thioureas (**2a**–**d**)

To a solution of 2-(5-amino-3-methyl-1*H*-pyrazol-1-yl)-1-phenylethanol **5** (217 mg, 1.0 mmol) in anhydrous THF (15 mL), the appropriate benzoyl isothiocyanate (1.1 mmol) was added, and the reaction mixture was heated at reflux for 12 h. After cooling to room temperature, the solvent was removed under reduced pressure. The crude product was dissolved in ethyl acetate (10 mL) and washed once with a saturated aqueous solution of NaHCO_3_ (10 mL), once with 3 M HCl (10 mL), and three times with brine (3 × 10 mL). The organic phase was dried (MgSO_4_) and concentrated under reduced pressure. The crude was then purified by flash or conventional column chromatography on silica gel, eluting with a mixture of diethyl ether/petroleum ether (bp 50–60 °C) (4:1), affording white solids.

##### General Synthesis of Pyrazolyl Benzamides (**3a**–**d**)

To a solution of 2-(5-amino-3-methyl-1*H*-pyrazol-1-yl)-1-phenylethanol **5** (217 mg, 1.0 mmol) in anhydrous THF (20 mL), triethylamine (TEA, 0.5 mL) was added, and the reaction mixture was cooled in an ice–water bath. The proper commercially available benzoyl chloride (1.0 mmol) was then added dropwise, and the reaction mixture was heated at gentle reflux for 18 h. After cooling to room temperature, the solvent was removed under reduced pressure. The crude product was solved in ethyl acetate (10 mL) and washed once with NaHCO_3_ saturated aqueous solution (10 mL), once with 3 M HCl (10 mL), and three times with brine (3 × 10 mL). The organic phase was dried (MgSO_4_) and concentrated under reduced pressure, affording white crystalline solids, which were recrystallized from absolute ethanol.

### Biological Section

#### General Information

All bacterial growth media used for the experiments were purchased from the Biolife Italiana srl- Mascia Brunelli spa– Milan (Italy) and prepared according to manufacturer’s procedures. Chemical reagents and reference antibiotics were purchased from Merck, Milan (Italy).

#### Bacterial Strains

Bacterial isolates used in this study were of clinical origin and belonged to a collection of strains from the DISC laboratories of the University of Genoa (Italy), which also included the *S*. *aureus* ATCC 29213 strain. They were isolated from human specimens and identified using the VITEK 2 technique (Biomerieux, Florence, Italy) or matrix-assisted laser desorption/ionization time-of-flight (MALDI-TOF) mass spectrometry (Biomerieux, Florence, Italy). Twenty-six were Gram-positive and three were Gram-negative for a total of twenty-eight strains. Among the Gram-positive species studied, there were 18 *Staphylococcus* strains, including 12 *S*. *aureus* (isolates A, B, C, D, 10, 11, 18, 24, 187, 189, N2, ATCC 29213—all MRSA except N2 and ATCC 29213) and 6 *S*. *epidermidis* (isolates 2, 22, 178, 180, 181, 198—all MRSE, with the last four also resistant to linezolid). Additionally, there were 7 *Enterococcus* strains: 4 *E*. *faecalis* (isolates 1a and 439 resistant to vancomycin and teicoplanin, and strains 365 and 431 resistant to vancomycin) and 3 *E*. *faecium* (isolates 179, 185, 186—all resistant to vancomycin and teicoplanin). The 3 Gram-negative isolates tested were all multiresistant (i.e., resistant to at least three different categories of antibiotics tested) and representative of *Escherichia coli*, *Klebsiella pneumoniae*, and *Pseudomonas aeruginosa* species.

#### Minimal Inhibitory Concentrations (MICs)

Values of the Minimal Inhibitory Concentration (MIC) were determined following the microdilution procedures as detailed by the European Committee on Antimicrobial Susceptibility Testing EUCAST (The European Committee on Antimicrobial Susceptibility Testing—EUCAST; Available online: https://www.eucast.org/).

Briefly, appropriate serial 2-fold dilutions in cation adjusted Mueller–Hinton (MH) broth of all the samples and reference antibiotics tested were used. Bacterial cultures obtained after overnight incubation were diluted to yield a standardized inoculum of 1.5 × 10^8^ CFU/mL. Aliquots of each suspension were added to 96-well microplates containing dilutions of compounds to be tested to yield a final concentration of about 5 × 10^5^ cells/mL. After 18 ± 2 h of incubation at 37 °C, the lowest concentration of sample that prevented visible growth was recorded as the MIC value. All MICs were obtained at least in triplicate; results were expressed by reporting the modal value.

#### Minimum Bactericidal Concentration (MBC) Determination

The minimum bactericidal concentration (MBC) of compounds **1c** and **1h** was determined from the same 96-well microplates previously used for MIC determination. After MIC reading, 25 μL aliquots were collected from wells showing no visible bacterial growth, including the well corresponding to the MIC and wells containing higher concentrations of the tested compound. Aliquots were plated onto Mueller–Hinton agar plates without the tested compound and incubated at 35–37 °C for 18–24 h under aerobic conditions.

After incubation, the plates were examined for bacterial colony growth. The MBC was defined as the lowest concentration of the tested compound resulting in no detectable colony growth, corresponding to a reduction of at least 99.9% of the initial bacterial inoculum. The experiment was performed in triplicate, and MBC values were reported as the modal values obtained from independent assays.

#### Time-Kill Curve Assays

Killing curve assays for compounds of interest were performed on three selected *S*. *aureus* isolates (18, 187, and A) as previously reported.^27^ Briefly, mid logarithmic phase bacterial cultures were diluted in MH broth (10 mL) containing 4× MIC of the two compounds to give a final inoculum of 5.0 × 10^5^ CFU/mL. The same inoculum was added to MH broth as a growth control. Tubes were incubated at 37 °C with constant shaking for 24 h. Samples of 0.20 mL from each tube were removed at 0, 2, 4, 6, and 24 h, diluted appropriately with a 0.9% sodium chloride solution to avoid carryover of compounds being tested, plated onto MH plates, and incubated for 24 h at 37 °C. Growth controls were run in parallel. The percentage of surviving bacterial cells was determined for each sampling time by comparing colony counts with those of standard dilutions of the growth control. Results have been expressed as log_10_ of viable cell numbers (CFU/mL) of surviving bacterial cells over a 24 h period. All time-kill curve experiments were performed in triplicate.

#### Biofilm Inhibition Assay

Inhibition of biofilm production was evaluated by the 96-well microplate (Euroclone-Milan, Italy) method, based on crystal-violet staining, with spectrophotometric quantification of the adherent mass according to a procedure described by Cramton et al.^43^ Selected bacterial strains were initially grown for 24 h in Tryptic Soy Broth (TSB) medium supplemented with 0.25% glucose to promote biofilm production. The stationary phase cultures were then diluted 1:100 in TSB-glucose and supplemented with the appropriate amount of test compounds or reference antibiotic at concentrations corresponding to 1/4 MIC, 1/2 MIC, and MIC of each strain. Growth controls were run in parallel. Each strain was evaluated for each condition in triplicate. The microplates were incubated for 24 h at 37 °C under static conditions. At the end of incubation, the contents of the wells were removed and each well was gently washed three times with saline to remove nonadherent cells. The plates were then allowed to air-dry and stained with 0.1% (w/v) crystal violet for 30 min at room temperature. Excess dye was removed by washing with saline, after which the plates were again allowed to air-dry. The crystal-violet permeated into the biofilm was solubilized with 95% ethanol; the absorbance of each well was read at 570 nm by a microplate spectrophotometer (CLARIOstar Plus - BMG LABECH), and all readings were subtracted from the blank value, corresponding to the absorbance of wells containing only TSB-glucose treated with crystal-violet and ethanol. Each experiment was repeated at least three times. The percentage of biofilm growth inhibition was calculated by the formula:

[1−(At/Ac)]×100

where Ac is the average absorbance measured for the control wells and At is the average absorbance measured in the presence of the compound under study.

#### Cytotoxicity Assay (MTT Test)

To perform the MTT assay, GM-6114 (embryonic human fibroblast, ATCC, Manassas, VA) cell lines were cultured in DMEM (including 10% FBS, 2 mM glutamine, and 1% penstrep). Reagents were acquired from EuroClone (Milan, Italy) and incubated in a humidified environment at 37 °C with 5% CO_2_. All chemical compounds were dissolved in DMSO to give a 10 mM stock solution. Then, after an intermediate dilution in growth medium, they were added to the cultured cells at a final working concentration of 100 μM and incubated for 48 h. At the end of the incubation, 30 μL of MTT (3-(4,5-dimethyl-2-thiazolyl)-2,5-diphenyl-2*H*-tetrazolium bromide) at a concentration of 2 mg/mL in PBS was added to each well and incubated 4 h. Finally, the supernatants were removed, and 100 μL of DMSO was added to each well to dissolve the Formazan precipitates. After 20 min, the results were read at 570 nm. Results are expressed as percentage of the control samples where cells have been treated with the same amount of DMSO but without any chemical compound. The assay was repeated three times, and a single compound was tested six times. Means and standard deviations were calculated.

## Supplementary Material



## Notes

^1^H and ^13^C NMR spectra for compounds **1**–**3** and **5**; HRMS (ESI) of compounds **1c**, **1h**, **2a**, and **3c**; HPLC chromatograms of compounds **1c** and **1h**; Bacterial growth of strain *S*. *aureus* ATCC 29213 and clinical isolates (**24**, **18**, **10**, **11**, **N2**, **189**, **A**, **B**, **C**, **D**, and **187**) treated with vancomycin at subinhibitory concentrations (1/4 MIC and 1/2 MIC) and at the minimum inhibitory concentration (MIC); Optical density at 570 nm (OD_570_) values and associated standard deviations measured after 24 h of incubation with vancomycin; MIC values of vancomycin against selected *S*. *aureus* strains expressed in μg/mL; Predicted physiochemical and pharmacokinetic properties of compounds **1c** and **1h** (PDF)

## Abbreviations

BBB: Blood–brain barrier
ECVAM: European Union Reference Laboratory for Alternatives to Animal Testing
MDR: Multidrug resistance
MIC: Minimum inhibitory concentration
MRSA: Methicillin-resistant *Staphylococcus aureus*
MRSE: Methicillin-resistant *Staphylococcus epidermidis*
NIH: National Institutes of Health
OD_570_: Optical density at 570 nm
PUs: Pyrazolyl-ureas
SARs: Structure–activity relationships
SD: Standard deviation
THF: Tetrahydrofuran
VRE: Vancomycin-resistant *Enterococcus faecalis*
VREF: Vancomycin-resistant *Enterococcus faecium*
