# Supplementary material for: Design, Synthesis, and Microbiological Evaluation of Differently Decorated Pyrazoles Able to Overcome Antimicrobial Resistance and Biofilm Formation
Source: ACS Omega. 2026 Sep 22;11(39):58851–61. doi: 10.1021/acsomega.6c03582 (PMC13647960; doi:10.1021/acsomega.6c03582)
Supplement: Supplementary file 1 [file ao6c03582_si_001.pdf]

Design, Synthesis and microbiological evaluation of differently decorated pyrazoles able to overcome antimicrobial resistance and biofilm formation

Matteo Lusardi [1], Debora Caviglia [1,2], Andrea Chiocca [1], Marco Ponassi [3], Gabriella Piatti [2], Annalisa Salis [4], Andrea Spallarossa [1], Anna Maria Schito [2], Chiara Brullo [1]*

[1] Department of Pharmacy (DIFAR), Section of Medicinal Chemistry, University of Genoa, Viale Benedetto XV, 3, 16132 Genoa, Italy;

[2] Department of Surgical Sciences and Integrated Diagnostics (DISC), University of Genoa, Viale Benedetto XV, 6, 16132 Genoa, Italy;

[3] Azienda Ospedaliera Metropolitana Genova, UOS Proteomica e Spettrometria di Massa, L.go. R. Benzi, 10, 16132 Genova, Italy;

[4] Department of Experimental Medicines (DIMES), University of Genoa, Largo Rosanna Benzi 10, PAD.3, 16132 Genoa, Italy.

Table of Contents

Chemistry section:

- Yield, melting point, ^{1}H and ^{13}C NMR data, elemental analysis, and HRMS spectra of intermediate compound **5** and final compounds **1–3**.
- **Figure S1**. ^{1}H NMR (400 MHz, DMSO-d_6) spectrum of compound **5**
- **Figure S2**. ^{13}C NMR (400 MHz, DMSO-d_6) spectrum of compound **5**
- **Figure S3**.^{1}H NMR (400 MHz, DMSO-d_6) spectrum of compound **1a**
- **Figure S4.** ^{13}C NMR (101 MHz, DMSO-d_6) spectrum of compound **1a**
- **Figure S5**. ^{1}H NMR (400 MHz, DMSO-d_6) spectrum of compound **1b**
- **Figure S6**. ^{13}C NMR (101 MHz, DMSO-d_6) spectrum of compound **1b**
- **Figure S7**. ^{1}H NMR (400 MHz, DMSO-d_6) spectrum of compound **1c**
- **Figure S8.** ^{13}C NMR (101 MHz, DMSO-d_6) spectrum of compound **1c**
- **Figure S9.** ^{1}H NMR (400 MHz, DMSO-d_6) spectrum of compound **1d**
- **Figure S10.** ^{13}C NMR (101 MHz, DMSO-d_6) spectrum of compound **1d**
- **Figure S11.** ^{1}H NMR (400 MHz, DMSO-d_6) spectrum of compound **1e**
- **Figure S12.** ^{13}C NMR (101 MHz, DMSO-d_6) spectrum of compound **1e**
- **Figure S13.** ^{1}H NMR (400 MHz, DMSO-d_6) spectrum of compound **1f**
- **Figure S14.** ^{13}C NMR (101 MHz, DMSO-d_6) spectrum of compound **1f**
- **Figure S15.** ^{1}H NMR (400 MHz, DMSO-d_6) spectrum of compound **1g**
- **Figure S16.** ^{13}C NMR (101 MHz, DMSO-d_6) spectrum of compound **1g**
- **Figure S17.** ^{1}H NMR (400 MHz, DMSO-d_6) spectrum of compound **1h**
- **Figure S18.** ^{13}C NMR (101 MHz, DMSO-d_6) spectrum of compound **1h**
- **Figure S19.** ^{1}H NMR (400 MHz, DMSO-d_6) spectrum of compound **2a**
- **Figure S20.** ^{13}C NMR (101 MHz, DMSO-d_6) spectrum of compound **2a**
- **Figure S21.** ^{1}H NMR (400 MHz, DMSO-d_6) spectrum of compound **2b**
- **Figure S22.** ^{13}C NMR (101 MHz, DMSO-d_6) spectrum of compound **2b**
- **Figure S23.** ^{1}H NMR (400 MHz, DMSO-d_6) spectrum of compound **2c**
- **Figure S24**. ^{13}C NMR (101 MHz, DMSO-d_6) spectrum of compound **2c**
- **Figure S25.** ^{1}H NMR (400 MHz, DMSO-d_6) spectrum of compound **2d**
- **Figure S26.** ^{13}C NMR (101 MHz, DMSO-d_6) spectrum of compound **2d**
- **Figure S27.** ^{1}H NMR (400 MHz, DMSO-d_6) spectrum of compound **3a**
- **Figure S28.** ^{13}C NMR (101 MHz, DMSO-d_6) spectrum of compound **3a**
- **Figure S29.** ^{1}H NMR (400 MHz, DMSO-d_6) spectrum of compound **3b**
- **Figure S30.** ^{13}C NMR (101 MHz, DMSO-d_6) spectrum of compound **3b**
- **Figure S31.** ^{1}H NMR (400 MHz, DMSO-d_6) spectrum of compound **3c**
- **Figure S32.** ^{13}C NMR (101 MHz, DMSO-d_6) spectrum of compound **3c**
- **Figure S33.** ^{1}H NMR (400 MHz, DMSO-d_6) spectrum of compound **3d**
- **Figure S34**. ^{13}C NMR (101 MHz, DMSO-d_6) spectrum of compound **3d**
- **Figure S35.** HPLC of compound **1c**
- **Figure S36.** HPLC of compound **1h**

Biological section:

Figures and Tables

- **Figure S37**. Bacterial growth of strain *S. aureus* ATCC 29213 and clinical isolates (**24**, **18**, **10**, **11**, **N2**, **189**, **A**, **B**, **C**, **D**, **187**) treated with vancomycin at sub-inhibitory concentrations (¼ MIC and ½ MIC) and at the minimum inhibitory concentration (MIC).
- **Table S1.** Optical density at 570 nm (OD_{570}) values and associated standard deviations measured after 24 h of incubation with vancomycin.
- **Table S2:** MIC values of vancomycin against selected *S. aureus* strains expressed in µg/mL.

Pharmacokinetics and drug-likeness properties of 1c and 1h (SwissADME)

Table S3. Predicted physicochemical and pharmacokinetic properties of compounds **1c** and **1h**.

Chemistry section

2-(5-amino-3-methyl-1*H*-pyrazol-1-yl)-1-phenylethanol 5. Yield: 60%. M.p. 100–102 °C. ^{1}H NMR (400 MHz, DMSO-d_6): δ 7.46-7.20 (m, 5H, Ar–H), 5.74-5.71 (m, OH, D_2O-exchangeable), 5.10 (s, 1H, CHpyr.), 4.95 (bs, 2H, NH2, D_2O-exchangeable), 4.90-4.85 (m, 1H, CHO), 3.92-3.78 (m, 2H, CH_2), 1.99 (s, 3H, CH_3). ^{13}C NMR (101 MHz, DMSO-d_6): δ 145.47, 142.99, 128.16, 127.31, 126.11, 88.20, 72.35, 53.89, 13.86. IR (KBr) ν (cm^{-1}): 3400–3100 (NH_2, OH). Anal. Calcd for $C_{12}H_{15}N_3O$: C, 66.34; H, 6.96; N, 19.34. Found: C, 66.12; H, 6.66; N, 19.22.

(1-(2-Hydroxy-2-phenylethyl)-3-methyl-1*H*-pyrazol-5-yl)-3-phenylurea (1a). Yield: 60%. M.p. 87–91°C. ^{1}H NMR (400 MHz, DMSO-d_6): δ 9.15 (bs, 1H, NH, D_2O exchangeable), 8.39 (bs, 1H, NH, D_2O exchangeable), 7.47-7.40 (m, 4H, Ar–H), 7.39-7.33 (m, 2H, Ar-H), 7.32-7.23 (m, 3H, Ar-H), 6.98-6.96 (m, 1H, Ar–H), 6.03 (s, 1H, CHpyr.), 5.88-5.85 (m, 1H, OH, D_2O exchangeable), 4.93 (m, 1H, CHO), 4.02-3.97 (m, 2H, CH_2), 2.12 (s, 3H, CH_3). ^{13}C NMR (101 MHz, DMSO-d_6): δ 152.09, 146.32, 143.20, 140.08, 138.72, 129.37, 128.73, 127.97, 126.60, 122.58, 118.78, 97.06, 72.52, 55.24, 14.34. Anal. Calcd for $C_{19}H_{20}N_4O_2$: C, 67.84; H, 5.99; N, 16.66. Found: C, 67.44; H, 5.63; N, 16.44.

1-(2-Fluorophenyl)-3-(1-(2-hydroxy-2-phenylethyl)-3-methyl-1*H*-pyrazol-5-yl)urea (1b). Yield: 45%. M.p. 129-132°C. ^{1}H NMR (400 MHz, DMSO-d_6): δ 8.97 (bs, 1H, NH, D_2O exchangeable), 8.76 (bs, 1H, NH, D_2O exchangeable), 8.12-8.08 (m, 1H, Ar–H), 7.45-7.39 (m, 2H, Ar–H), 7.38-7.31 (m, 2H, Ar–H), 7.30-7.20 (m, 2H, Ar–H), 7.16-7.10 (m, 1H, Ar–H), 7.05-6.98 (m, 1H, Ar–H), 6.05 (s, 1H, CHpyr.), 5.74-5.77 (m, 1H, OH, D_2O exchangeable), 4.96-4.92 (m, 1H, CHO), 4.00-3.98 (m, 2H, CH_2), 2.12 (s, 3H, CH_3). ^{13}C NMR (101 MHz, DMSO-d_6): δ 152.66 (d, $J_{C–F}$ = 242 Hz), 151.74, 146.65, 143.25, 138.52, 128.69, 127.92, 127.85 (d, J_{C-F} = 11 Hz), 126.60, 125.09 (d, $J_{C–F}$ = 3 Hz), 123.31 (d, $J_{C–F}$ = 7 Hz), 121.32 (d, $J_{C–F}$ = 2 Hz), 115.60 (d, $J_{C–F}$ = 19 Hz), 96.62, 72.27, 55.10, 14.35. Anal. Calcd for $C_{19}H_{19}FN_4O_2$: C, 64.40; H, 5.40; F, 5.36; N, 15.81. Found: C, 64.30; H, 5.22; F, 5.00; N, 15.41.

1-(3-Fluorophenyl)-3-(1-(2-hydroxy-2-phenylethyl)-3-methyl-1*H*-pyrazol-5-yl)urea (1c). Yield: 53%. M.p. 90-93°C. ^{1}H NMR (400 MHz, DMSO-d$_6$): δ 9.36 (bs, 1H, NH, D_2O exchangeable), 8.42 (bs, 1H, NH, D_2O exchangeable), 7.51–7.27 (m, 7H, Ar–H), 7.17–7.11 (m, 1H, Ar–H), 6.87–6.63 (m, 1H, Ar–H), 6.03 (s, 1H, CHpyr.), 5.86-5.88 (m, 1H, OH, D_2O exchangeable), 4.94–4.90 (m, 1H, CHO), 4.02–3.96 (m, 2H, CH_2), 2.13 (s, 3H, CH_3). ^{13}C NMR (101 MHz, DMSO-d$_6$): δ 162.42 (d, $J_{C–F}$ = 242 Hz), 151.47, 145.83, 142.66, 141.48 (d, $J_{C–F}$ = 11 Hz), 137.89, 130.43 (d, $J_{C–F}$ = 10 Hz), 128.24, 127.49, 126.10, 113.99, 108.41 (d, $J_{C–F}$ = 21 Hz), 104.93 (d, $J_{C–F}$ = 26 Hz), 96.83, 72.04, 54.78, 13.83. The HRMS (ESI) calculated for $C_{19}H_{20}FN_4O_2$ $[M+H]^+$ 355.15702 found 355.15657. HPLC purity: 97.35%. Anal. Calcd for $C_{19}H_{19}FN_4O_2$: C, 64.40; H, 5.40; N, 15.81. Found: C, 64.35; H, 5.42; N, 15.49.

1-(4-fluorophenyl)-3-(1-(2-hydroxy-2-phenylethyl)-3-methyl-1*H*-pyrazol-5-yl)urea (1d). Yield: 25%. M.p. 180–182°C**.** ^{1}H NMR (400 MHz, DMSO-d$_6$): δ 9.16 (bs, 1H, NH, D_2O exchangeable), 8.35 (bs, 1H, NH, D_2O exchangeable), 7.49–7.40 (m, 4H, Ar–H), 7.39–7.32 (m, 2H, Ar–H), 7.31–7.24 (m, 1H, Ar–H), 7.16–7.08 (m, 2H, Ar–H), 6.01 (s, 1H, CHpyr.), 5.85-5.88 (m, 1H, OH, D_2O exchangeable), 4.94–4.90 (m, 1H, CHO), 4.05–3.91 (m, 2H, CH_2), 2.12 (s, 3H, CH_3). ^{13}C NMR (101 MHz, DMSO-d$_6$): δ 157.99 (d, $J_{C–F}$ = 239 Hz), 152.22, 146.30, 143.19, 138.65, 136.42, 128.73, 127.97, 126.59, 120.59 (d, $J_{C–F}$ = 7 Hz), 115.87 (d, $J_{C–F}$ = 22 Hz), 97.22, 72.53, 55.27, 14.34. Anal. Calcd for $C_{19}H_{19}FN_4O_2$: C, 64.40; H, 5.40; N, 15.81. Found: C, 64.35; H, 5.29; N, 15.79.

1-(3-Chlorophenyl)-3-(1-(2-hydroxy-2-phenylethyl)-3-methyl-1*H*-pyrazol-5-yl)urea (1e). Yield: 44%. M.p. 97–99°C. ^{1}H NMR (400 MHz, DMSO-d$_6$): δ 9.38 (bs, 1H, NH, D_2O exchangeable), 8.45 (bs, 1H, NH, D_2O exchangeable), 7.78–7.70 (m, 1H, Ar–H), 7.48–7.24 (m, 7H, Ar–H), 7.06–6.99 (m, 1H, Ar–H), 6.04 (s, 1H, CHpyr.), 5.90-5.93 (m, 1H, OH, D_2O exchangeable), 4.95–4.91 (m, 1H, CHO), 4.03–3.99 (m, 2H, CH_2), 2.13 (s, 3H, CH_3). ^{13}C NMR (101 MHz, DMSO-d$_6$): δ 151.52, 145.90, 142.69, 141.21, 137.97, 133.31, 130.53, 128.25, 127.53, 126.13, 121.76, 117.70, 116.72, 96.94, 72.11, 54.84, 13.88. Anal. Calcd for $C_{19}H_{19}ClN_4O_2$: C, 61.54; H, 5.16; N, 15.11. Found: C, 61.34; H, 5.00; N, 15.03.

1-(3-Bromophenyl)-3-(1-(2-hydroxy-2-phenylethyl)-3-methyl-1*H*-pyrazol-5-yl)urea (1f) Yield: 58%. M.p. 102–104°C. ^{1}H NMR (400 MHz, DMSO-d_6): δ 8.44 (bs, 1H, NH, D_2O exchangeable), 7.86 (bs, 1H, NH, D_2O exchangeable), 7.45–7.12 (m, 9H, Ar–H), 6.04 (s, 1H, CHpyr.), 5.89-5.92 (m, 1H, OH, D_2O exchangeable), 4.95–4.90 (m, 1H, CHO), 4.04–3.95 (m, 2H, CH_2), 2.13 (s, 3H, CH_3). ^{13}C NMR (101 MHz, DMSO-d_6): δ 151.50, 145.88, 142.68, 141.34, 137.92, 130.83, 128.27, 121.83, 120.71, 120.53, 117.28, 117.09, 96.92, 72.11, 54.85, 13.87. Anal. Calcd for $C_{19}H_{19}BrN_4O_2$: Calcd: C, 54.95; H, 4.61; N, 13.49. Found: C, 54.66; H, 4.32; N, 13.33.

1-(1-(2-Hydroxy-2-phenylethyl)-3-methyl-1*H*-pyrazol-5-yl)-3-(m-tolyl)urea (1g). Yield: 49%. M.p. 142-143°C. ^{1}H NMR (400 MHz, DMSO-d_6): δ 9.06 (bs, 1H, NH, D_2O exchangeable), 8.35 (bs, 1H, NH, D_2O exchangeable), 7.45–7.41 (m, 2H, Ar–H), 7.39–7.33 (m, 2H, Ar–H), 7.31–7.28 (m, 2H, Ar–H), 7.27–7.20 (m, 1H, Ar–H), 7.18–7.13 (m, 1H, Ar–H), 6.81–6.73 (m, 1H, Ar–H), 6.02 (s, 1H, CHpyr.), 5.85-5.87 (m, 1H, OH, D_2O exchangeable), 4.94–4.90 (m, 1H, CHO), 3.98 (m, 2H, CH_2), 2.27 (s, 3H, CH_3), 2.12 (s, 3H, CH_3). ^{13}C NMR (101 MHz, DMSO-d_6): δ 151.54, 145.78, 142.71, 139.50, 138.24, 138.02, 128.70, 128.22, 127.46, 126.09, 122.80, 118.80, 115.44, 96.48, 71.99, 54.73, 21.25, 13.84. Anal. Calcd for $C_{20}H_{22}N_4O_2$: C, 68.55; H, 6.33; N, 15.99. Found: C, 68.20; H, 6.00; N, 15.66.

1-(1-(2-Hydroxy-2-phenylethyl)-3-methyl-1*H*-pyrazol-5-yl)-3-(3 (trifluoromethyl) phenyl)urea (1h). Yield: 47%. M.p. 103–105°C. ^{1}H NMR (400 MHz, DMSO-d_6): δ 9.54 (bs, 1H, D_2O exchangeable), 8.49 (bs, 1H, D_2O exchangeable), 8.01-7.99 (m, 1H, Ar–H), 7.63–7.56 (m, 1H, Ar–H), 7.55–7.48 (m, 1H, Ar–H), 7.46–7.38 (m, 1H, Ar–H), 7.38–7.25 (m, 4H, Ar–H), 6.04 (s, 1H, CHpyr), 5.90-5.92 (m, 1H, OH, D_2O exchangeable), 4.95–4.92 (m, 1H, CHO), 4.07–3.90 (m, 2H, CH_2), 2.18–2.10 (m, 3H, CH_3).^{13}C NMR (101 MHz, DMSO-d_6): δ 151.59, 145.80, 142.61, 140.46, 137.76, 129.99, 128.98 (q, J = 31.3 Hz), 128.20, 127.45, 126.06, 124.20 (q, J = 272.5 Hz), 121.81, 118.28 (q, J = 3.6 Hz), 114.17 (q, J = 3.9 Hz), 97.00, 72.05, 54.80, 13.79. The HRMS (ESI) calculated for $C_{20}H_{20}F_3N_4O_2$ $[M+H]^+$ 405.15382 found 405.15336. HPLC purity: 95.44%. Anal. Calcd for $C_{20}H_{19}F_3N_4O_2$: C, 59.40; H, 4.74; N, 13.85. Found: C, 59.20; H, 5.07; N, 13.60.

N-((1-(2-Hydroxy-2-phenylethyl)-3-methyl-1*H*-pyrazol-5-yl)carbamothioyl)benzamide (2a). Yield: 44%. M.p. 185-187°C. ^{1}H NMR (400 MHz, DMSO-d_6): δ 11.82 (bs, 1H, NH, D_2O exchangeable), 8.03–7.96 (m, 2H, Ar–H), 7.64–7.61 (m, 1H, Ar–H), 7.61–7.51 (m, 2H, Ar–H), 7.46–7.38 (m, 2H, Ar–H), 7.37–7.31 (m, 2H, Ar–H), 7.30–7.23 (m, 1H, Ar–H), 6.44 (s, 1H, CHpyr.), 5.80-5.83 (m, 1H, OH, D_2O exchangeable), 4.90–4.86 (m, 1H, CH), 4.09–3.99 (m, 2H, CH_2), 2.18 (s, 3H, CH_3). ^{13}C NMR (101 MHz, DMSO-d_6): δ 179.18, 168.25, 145.83, 142.54, 137.40, 133.26, 132.01, 128.81, 128.49, 128.22, 127.53, 126.08, 100.18, 72.01, 55.32, 13.83. The HRMS (ESI) calculated for $C_{20}H_{21}N_4O_2S$ $[M+H]^+$ 381.13851found 381.13814. Anal. Calcd for $C_{20}H_{20}N_4O_2S$: C, 63.14; H, 5.30; N, 14.73; S, 8.43. Found: C, 63.14; H, 5.18; N, 14.92; S, 8.45.

2-Fluoro-N-((1-(2-hydroxy-2-phenylethyl)-3-methyl-1*H*-pyrazol-5-yl)carbamothioyl) benzamide (2b). Yield: 20%. M.p. 182-185°C**.** ^{1}H NMR (400 MHz, DMSO-d_6): δ 12.42 (bs, 1H, NH, D_2O exchangeable), 11.97 (bs, 1H, NH, D_2O exchangeable), 7.76–7.25 (m, 9H, Ar–H), 6.45 (s, 1H, CHpyr.), 5.85-5.87 (m, 1H, OH, D_2O exchangeable), 4.88–4.84 (m, 1H, CH), 4.09–3.97 (m, 2H, CH_2), 2.18 (s, 3H, CH_3). ^{13}C NMR (101 MHz, DMSO-d_6): δ 178.44, 165.24, 160.56 (d, $J_{C\text{–}F}$ = 252 Hz), 145.83, 142.50, 137.27, 134.26 (d, $J_{C\text{–}F}$ = 8.7 Hz), 130.47 (d, $J_{C\text{–}F}$ = 2.4 Hz), 128.23, 128.12, 127.55, 126.06 (d, $J_{C\text{–}F}$ = 2.8 Hz), 124.62 (d, $J_{C\text{–}F}$ = 3.4 Hz), 122.16, 122.03, 116.35, 116.14, 100.22, 72.05, 55.35, 13.81. Anal. Calcd for $C_{20}H_{19}FN_4O_2S$: C, 60.29; H, 4.81; N, 14.06; S, 8.05. Found: C, 60.44; H, 4.74; N, 14.46; S, 8.30**.**

3-Fluoro-N-((1-(2-hydroxy-2-phenylethyl)-3-methyl-1*H*-pyrazol-5-yl)carbamothioyl) benzamide (2c). Yield: 41%. M.p. 171-174°C. ^{1}H NMR (400 MHz, DMSO-d_6): δ 11.93 (bs, 1H, NH, D_2O exchangeable), 7.87–7.28 (m, 9H, Ar–H), 6.44 (s, 1H, CHpyr.), 5.84-5.86 (m, 1H, OH, D_2O exchangeable), 4.90–4.87 (m, 1H, CH), 4.16–3.99 (m, 2H, CH_2), 2.19 (s, 3H, CH_3). ^{13}C NMR (101 MHz, DMSO-d_6): δ 179.02, 166.90, 160.41 (d, $J_{C\text{–}F}$ = 244 Hz), 145.82, 142.50, 137.32, 134.27 (d, $J_{C\text{–}F}$ = 7 Hz), 130.63 (d, $J_{C\text{–}F}$ = 9 Hz), 128.22, 127.52, 126.08, 125.06 (d, $J_{C\text{–}F}$ = 3 Hz), 120.19, 119.98,

115.53 (d, J_{C-F} = 23 Hz), 100.24, 72.01, 55.29, 13.8. Anal. Calcd for $C_{20}H_{19}FN_4O_2S$: C, 60.29; H, 4.81; N, 14.06; S, 8.05. Found: C, 60.22; H, 4.46; N, 13.86; S, 8.35**.**

4-Fluoro-N-((1-(2-hydroxy-2-phenylethyl)-3-methyl-1*H*-pyrazol-5-yl)carbamothioyl) benzamide (2d). Yield: 75%. M.p. 181-188°C. ^{1}H NMR (400 MHz, DMSO-d$_6$): δ 11.87 (bs, 1H, NH, D_2O exchangeable), 8.12–8.06 (m, 2H, Ar-H), 7.44–7.32 (m, 6H, Ar-H), 7.31–7.25 (m, 1H, Ar-H), 6.43 (s, 1H, CHpyr.), 5.80-5.82 (m, 1H, OH, D_2O exchangeable), 4.89–4.85 (m, 1H, CH), 4.09–3.98 (m, 2H, CH_2), 2.18 (s, 3H, CH_3). ^{13}C NMR (101 MHz, DMSO-d$_6$): δ 179.15, 167.16, 166.28 (d, J_{C-F} = 253 Hz), 145.82, 142.54, 137.38, 131.94 (d, J_{C-F} = 16 Hz), 128.56, 128.24, 127.54, 126.09, 115.68 (d, J_{C-F} = 16 Hz), 100.20, 72.01, 55.31, 13.84. Anal. Calcd for $C_{20}H_{19}FN_4O_2S$: C, 60.29; H, 4.81; N, 14.06; S, 8.05. Found: C, 60.14; H, 4.79; N, 13.97; S, 8.26**.**

***N*-(1-(2-hydroxy-2-phenylethyl)-3-methyl-1*H*-pyrazol-5-yl)benzamide (3a).** Yield: 55%. M.p. 199-201°C. ^{1}H NMR (400 MHz, DMSO-d$_6$): δ 10.45 (bs, 1H, NH, D_2O exchangeable), 7.94–7.29 (m, 10H, Ar-H), 6.20 (s, 1H, CHpyr.), 6.10-6.12 (m, 1H, OH, D_2O exchangeable), 4.99–4.94 (m, 1H, CH), 4.29–4.09 (m, 2H, CH_2), 2.14 (s, 3H, CH_3). ^{13}C NMR (101 MHz, DMSO-d$_6$): δ 164.76, 146.28, 142.95, 137.76, 134.17, 132.61, 129.84, 129.19, 129.03, 128.73, 128.45, 128.11, 127.99, 126.53, 98.55, 72.94, 56.31, 14.28. Anal. Calcd for $C_{19}H_{19}N_3O_2$: C, 71.01; H, 5.96; N, 13.08. Found: C, 71.40; H, 5.61; N, 13.15.

2-Fluoro-N-(1-(2-hydroxy-2-phenylethyl)-3-methyl-1*H*-pyrazol-5-yl)benzamide (3b). Yield: 53%. M.p. 225-228 °C. ^{1}H NMR (400 MHz, DMSO-d$_6$): δ 10.76 (bs, 1H, NH, D_2O exchangeable), 7.79–7.62 (m, 2H, Ar-H), 7.44–7.26 (m, 7H, Ar-H), 6.48 (s, 1H, CHpyr.), 5.04–5.01 (m, 1H, CH), 4.39–4.27 (m, 2H, CH_2), 2.26 (s, 3H, CH_3).^{13}C NMR (101 MHz, DMSO-d$_6$): δ 161.71, 160.72 (d, J_{C-F} = 252 Hz), 145.52, 142.03, 138.62, 133.86 (d, J_{C-F} = 9 Hz), 130.62, 128.29, 127.66, 126.07, 124.87 (d, J_{C-F} = 3 Hz), 122.63 (d, J_{C-F} = 13 Hz), 116.60 (d, J_{C-F} = 22 Hz), 98.43, 71.61, 55.10, 12.74. Anal. Calcd for $C_{19}H_{18}FN_3O_2$: C, 67.24; H, 5.35; N, 12.38. Found: C, 67.66; H, 5.84; N, 12.24.

3-Fluoro-N-(1-(2-hydroxy-2-phenylethyl)-3-methyl-1*H*-pyrazol-5-yl)benzamide (3c). Yield: 56%. M.p. 188-190°C**.** ^{1}H NMR (400 MHz, DMSO-d$_6$): δ 10.48 (bs, 1H, NH, D_2O exchangeable),

7.78–7.23 (m, 9H, Ar-H), 6.19 (s, 1H, CHpyr.), 6.15 (m, 1H, OH, D_2O exchangeable), 4.99–4.95 (m, 1H, CH), 4.31–4.10 (m, 2H, CH_2), 2.14 (s, 3H, CH_3). ^{13}C NMR (101 MHz, DMSO-d_6): δ 161.88 (d, $J_{C–F}$ = 224 Hz), 145.82, 142.44, 136.98, 135.98 (d, $J_{C–F}$ = 7 Hz), 130.89 (d, $J_{C–F}$ = 8 Hz), 128.18, 127.44, 126.00, 123.80 (d, $J_{C–F}$ = 3 Hz), 118.99 (d, $J_{C–F}$ = 21 Hz), 114.51 (d, $J_{C–F}$ = 23 Hz), 98.20, 72.33, 55.70, 13.74. The HRMS (ESI) calculated for $C_{19}H_{19}FN_3O_2$ $[M+H]^+$ 340.14612 found 340.14582. Anal. Calcd for $C_{19}H_{18}FN_3O_2$: C, 67.24; H, 5.35; N, 12.32. Found: C, 67.28; H, 5.45; N, 12.48.

4-Fluoro-N-(1-(2-hydroxy-2-phenylethyl)-3-methyl-1*H*-pyrazol-5-yl)benzamide (3d). Yield: 60%. M.p. 201-204°C. ^1H NMR (400 MHz, DMSO-d_6): δ 10.42 (bs, 1H, NH, D_2O exchangeable), 7.97–8.02 (m, 2H, Ar-H), 7.44–7.23 (m, 7H, Ar-H), 6.18 (s, 1H, CHpyr.), 6.11-6.14 (m, 1H, OH, D_2O exchangeable), 4.98–4.95 (m, 1H, CH), 4.28–4.09 (m, 2H, CH_2), 2.14 (s, 3H, CH_3). ^{13}C NMR (101 MHz, DMSO-d_6): δ 164.42 (d, $J_{C–F}$ = 232 Hz), 163.09, 145.77, 142.45, 137.17, 130.39 (d, $J_{C–F}$ = 9 Hz), 130.14 (d, $J_{C–F}$ = 3 Hz), 128.66, 128.18, 127.44, 126.37, 126.00, 115.64 (d, $J_{C–F}$ = 21 Hz), 98.16, 72.33, 55.70, 13.75. Anal. Calcd for $C_{19}H_{18}FN_3O_2$: C, 67.24; H, 5.35; N, 12.32. Found: C, 67.18; H, 5.05; N, 12.42.

Figure S1. ^{1}H NMR (400 MHz, DMSO-d$_6$) spectrum of compound **5**

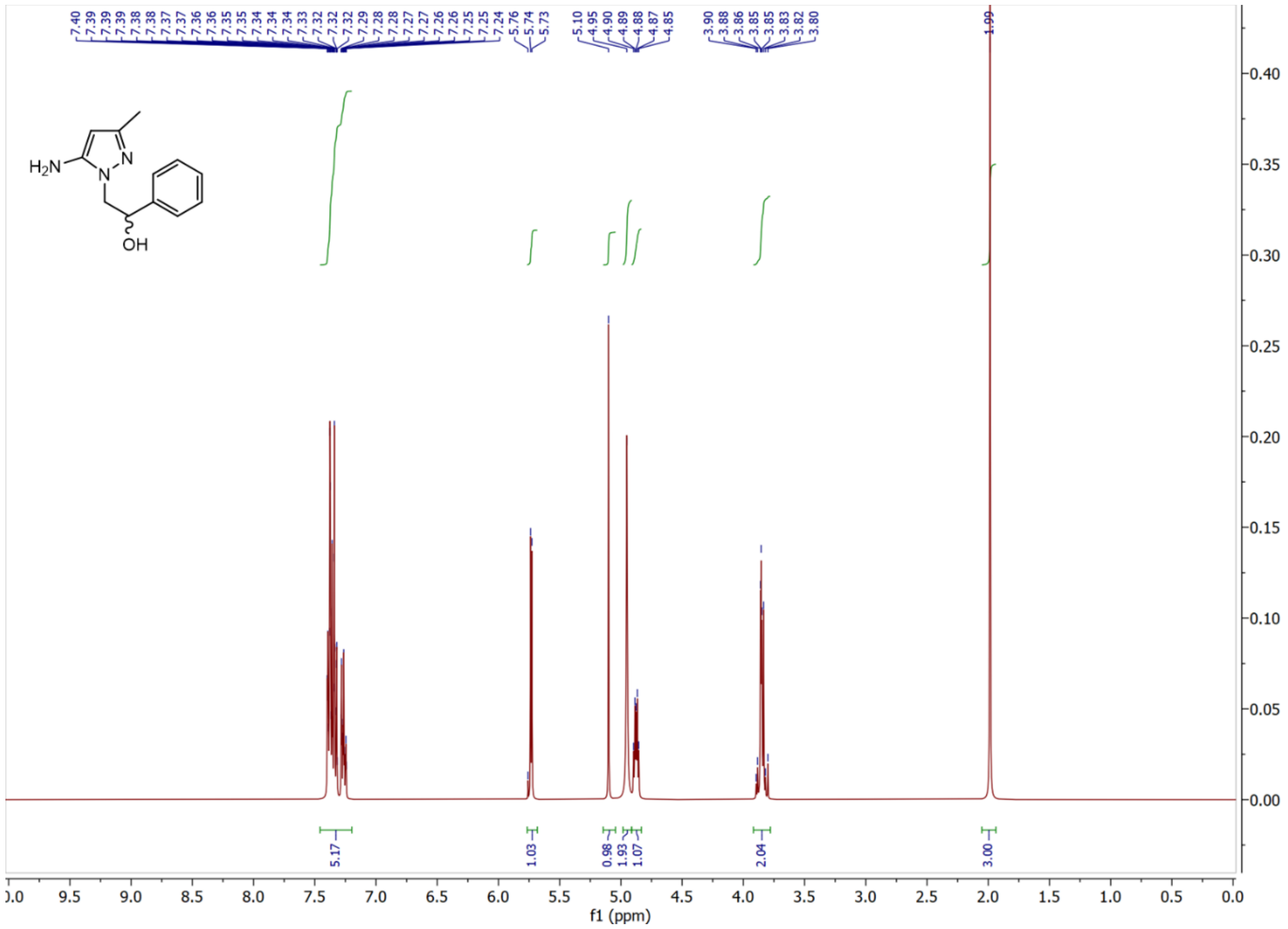

Figure S2. ^{13}CNMR (400 MHz, DMSO-d$_6$) spectrum of compound **5**

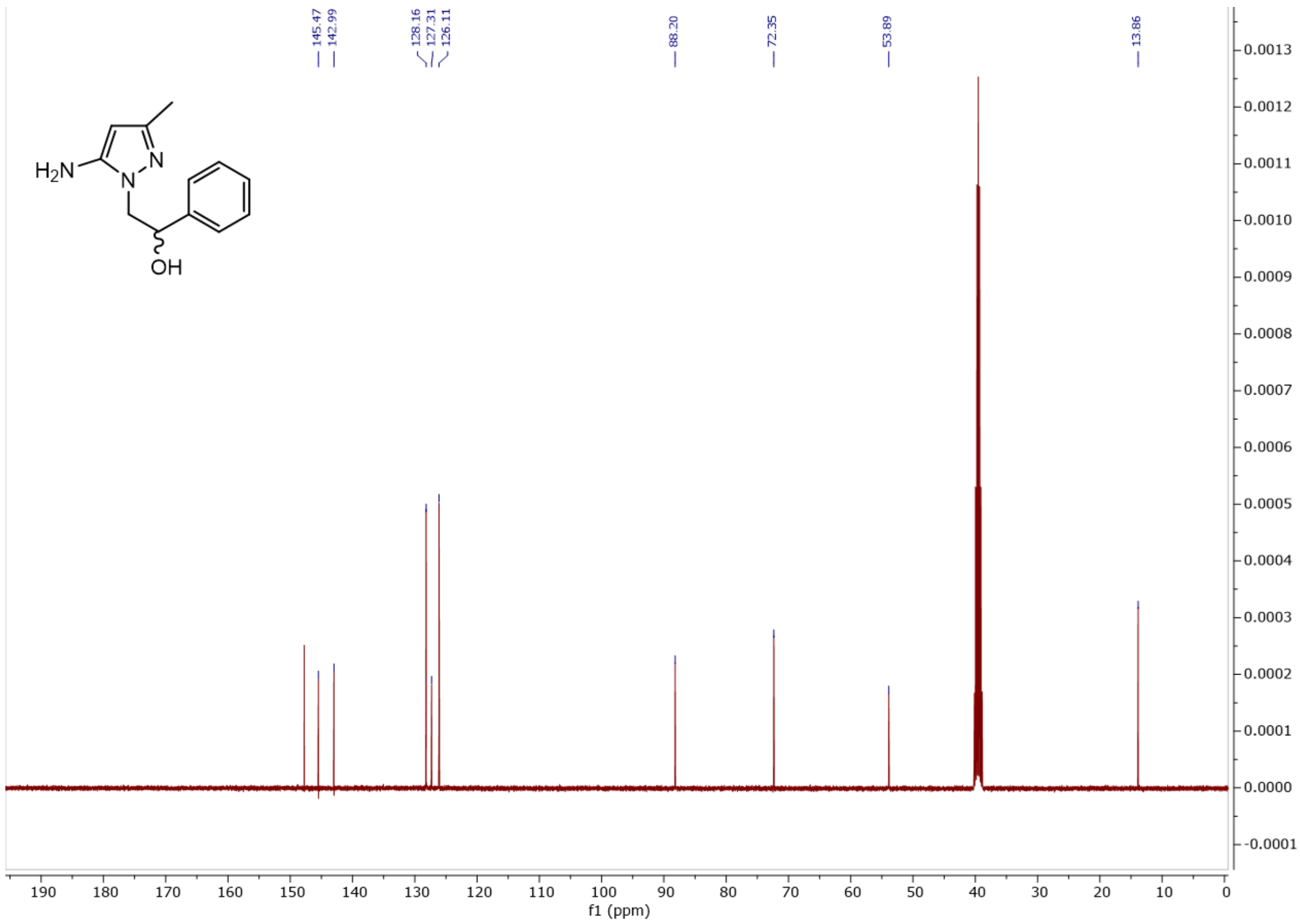

Figure S3. ^{1}H NMR (400 MHz, DMSO-d$_6$) spectrum of compound **1a**

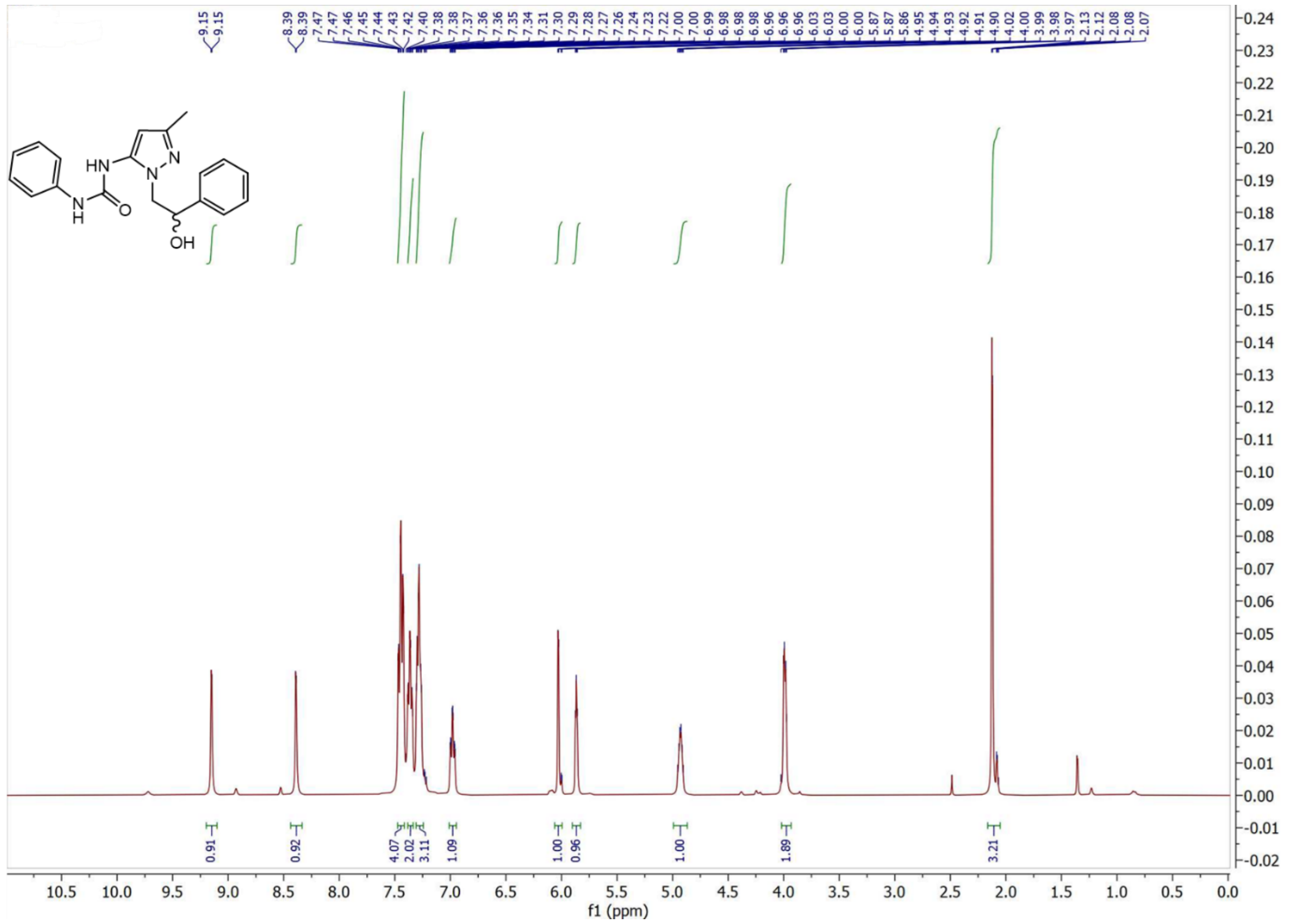

Figure S4. ^{13}C NMR (101 MHz, DMSO-d$_6$) spectrum of compound **1a**

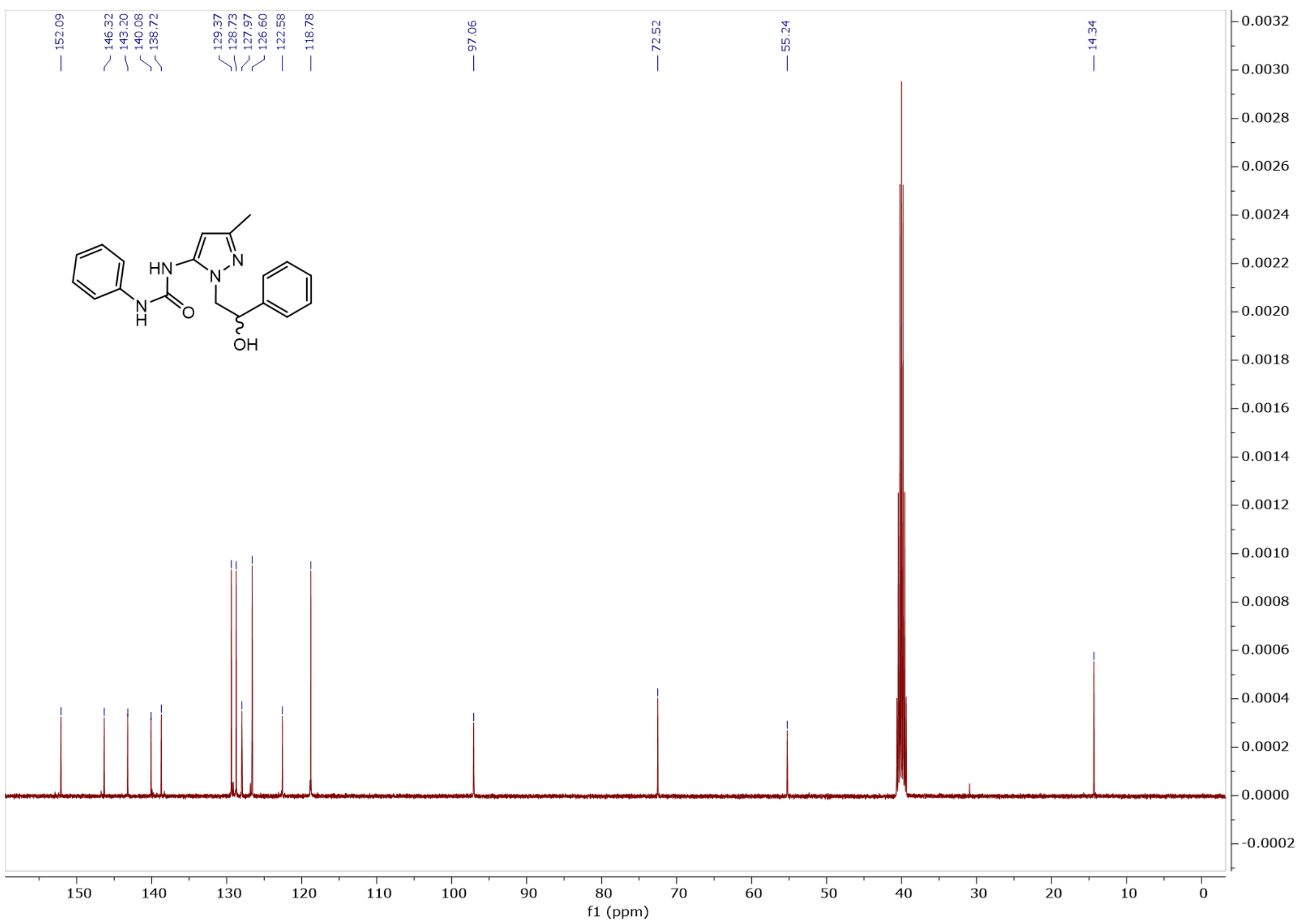

Figure S5. ^{1}H NMR (400 MHz, DMSO-d_6) spectrum of compound **1b**

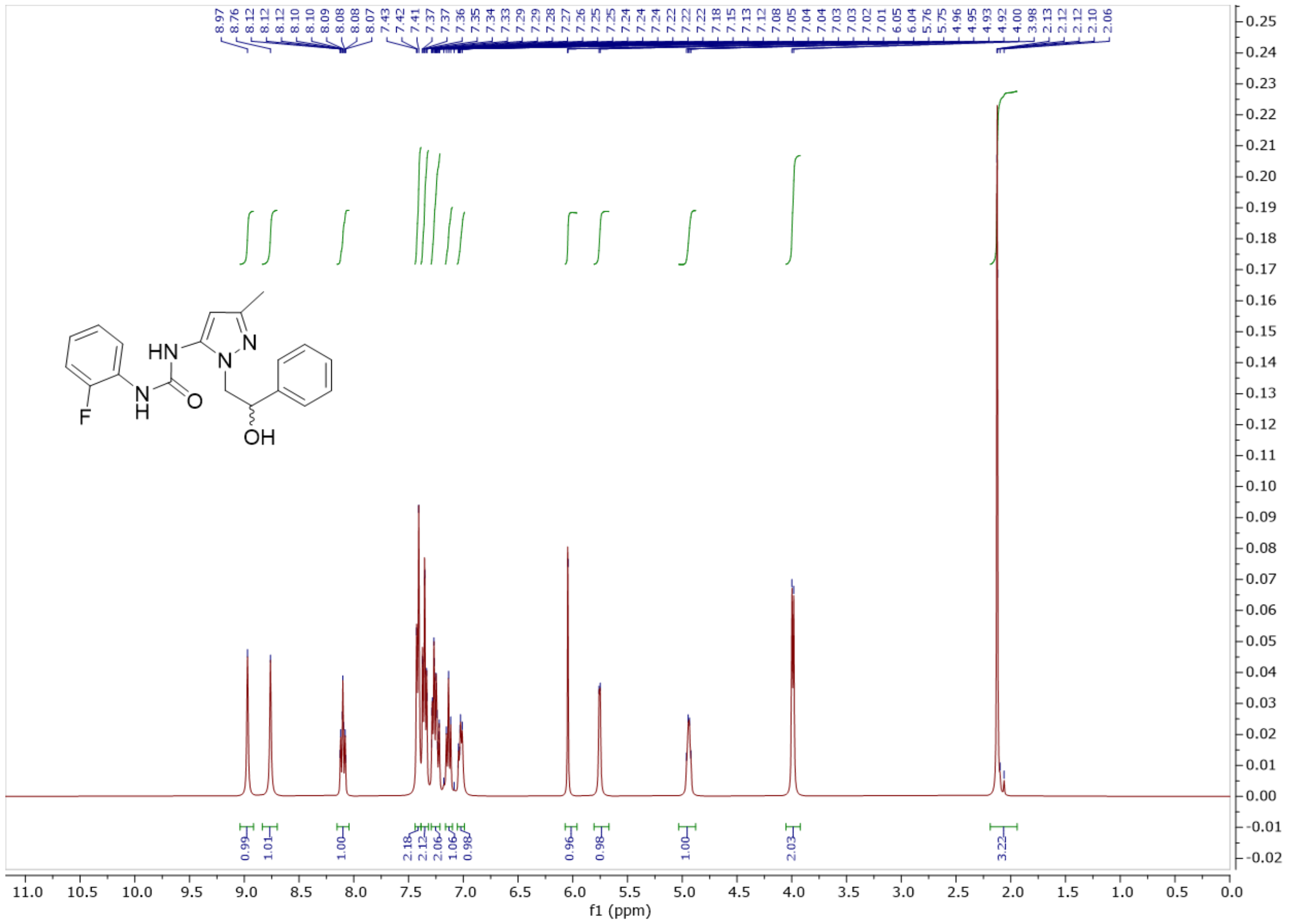

Figure S6. ^{13}C NMR (101 MHz, DMSO-d_6) spectrum of compound **1b**

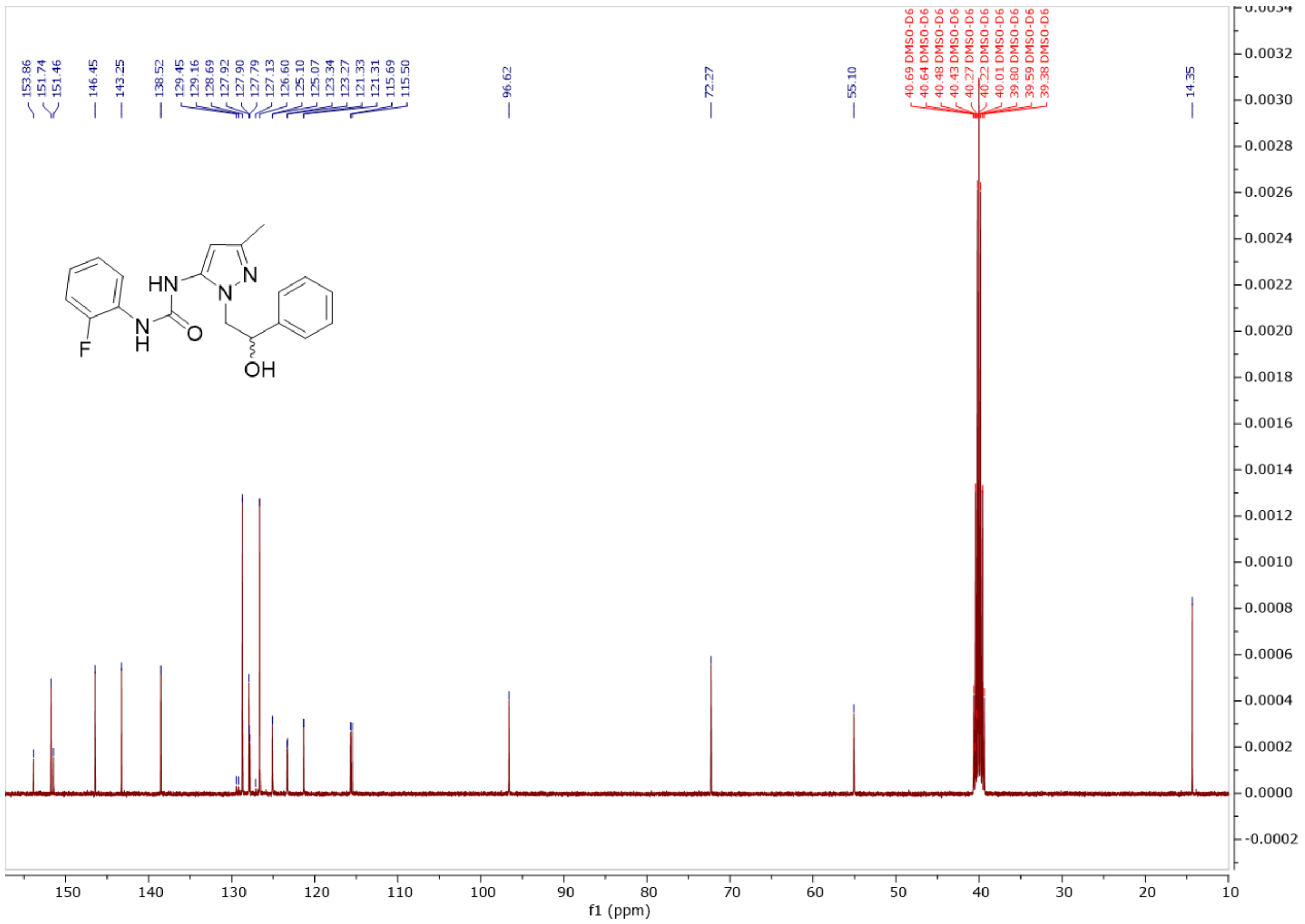

Figure S7. ^{1}H NMR (400 MHz, DMSO-d$_6$) spectrum of compound **1c**

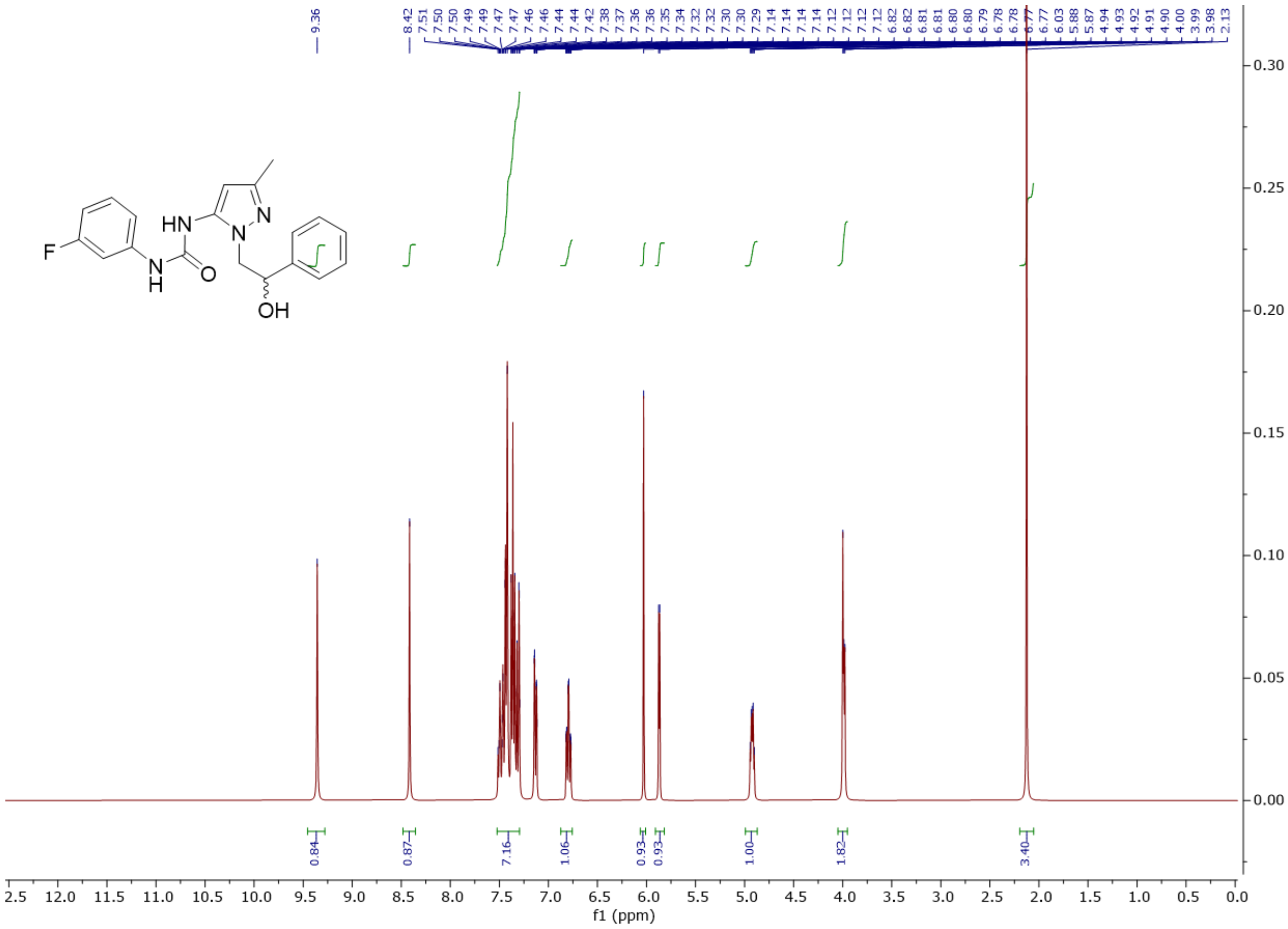

Figure S8. ^{13}C NMR (101 MHz, DMSO-d$_6$) spectrum of compound **1c**

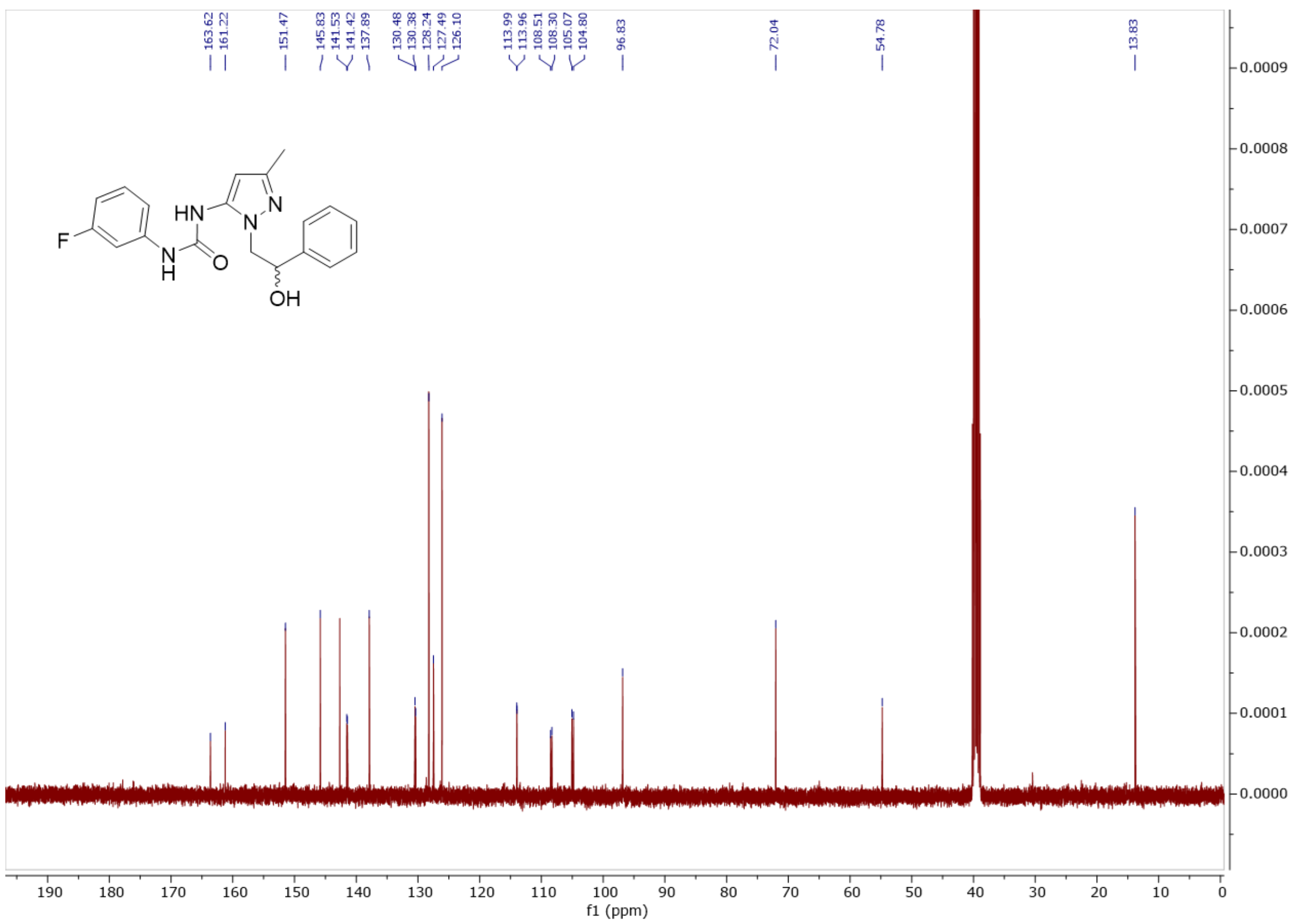

Figure S9. ^{1}H NMR (400 MHz, DMSO-d$_6$) spectrum of compound **1d**

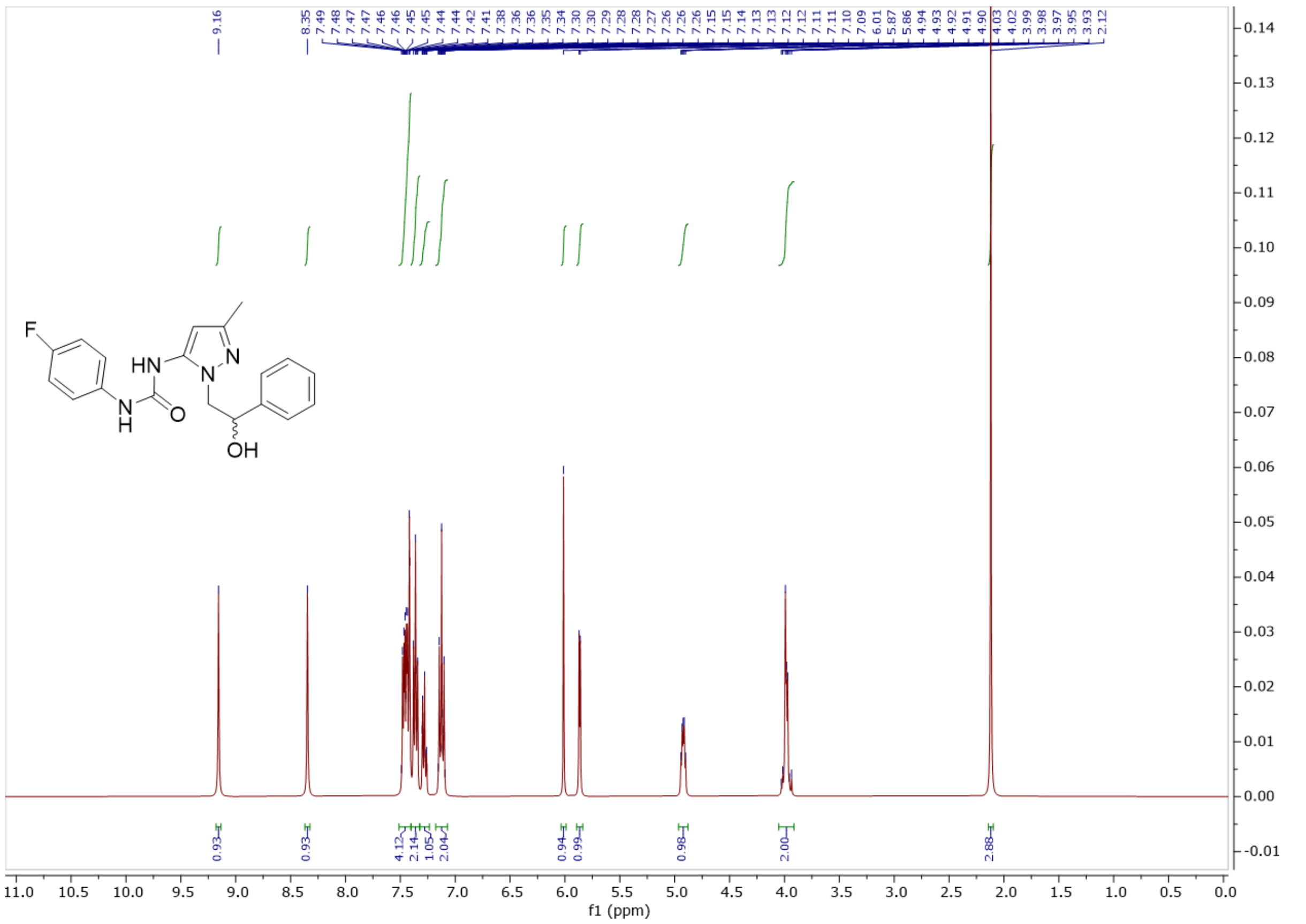

Figure S10. ^{13}C NMR (101 MHz, DMSO-d$_6$) spectrum of compound **1d**

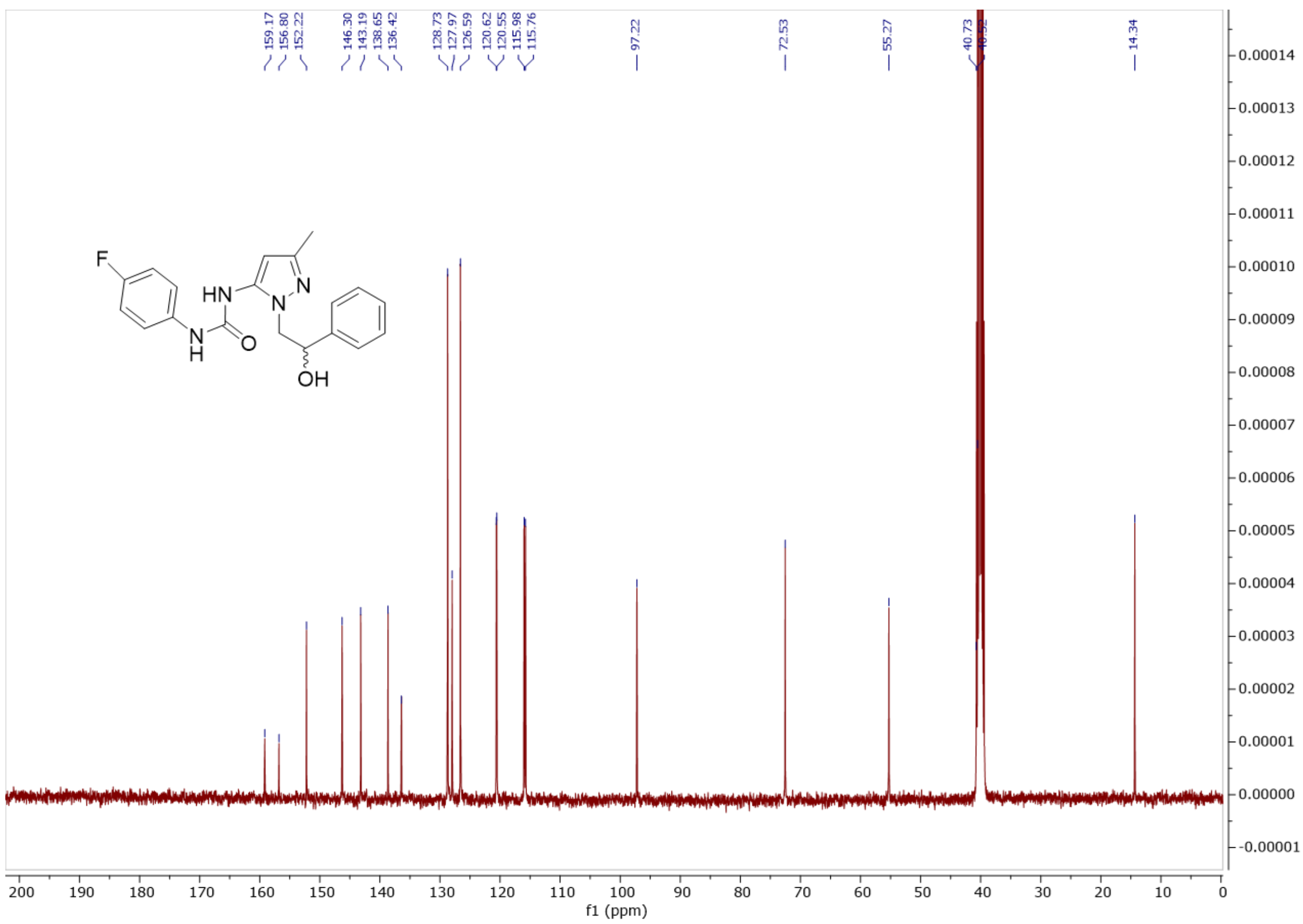

Figure S11. ^{1}H NMR (400 MHz, DMSO-d_6) spectrum of compound **1e**

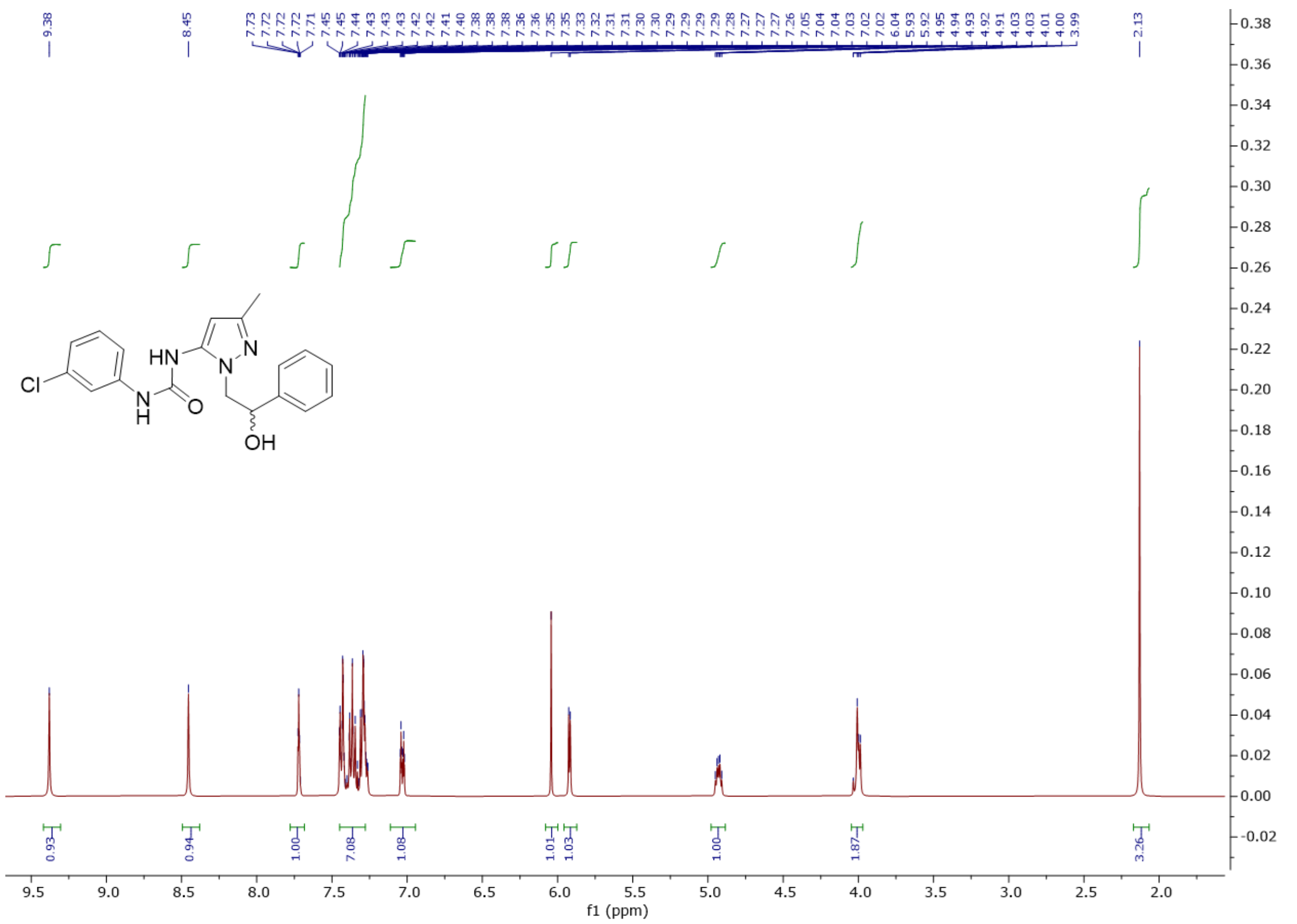

Figure S12. ^{13}C NMR (101 MHz, DMSO-d_6) spectrum of compound **1e**

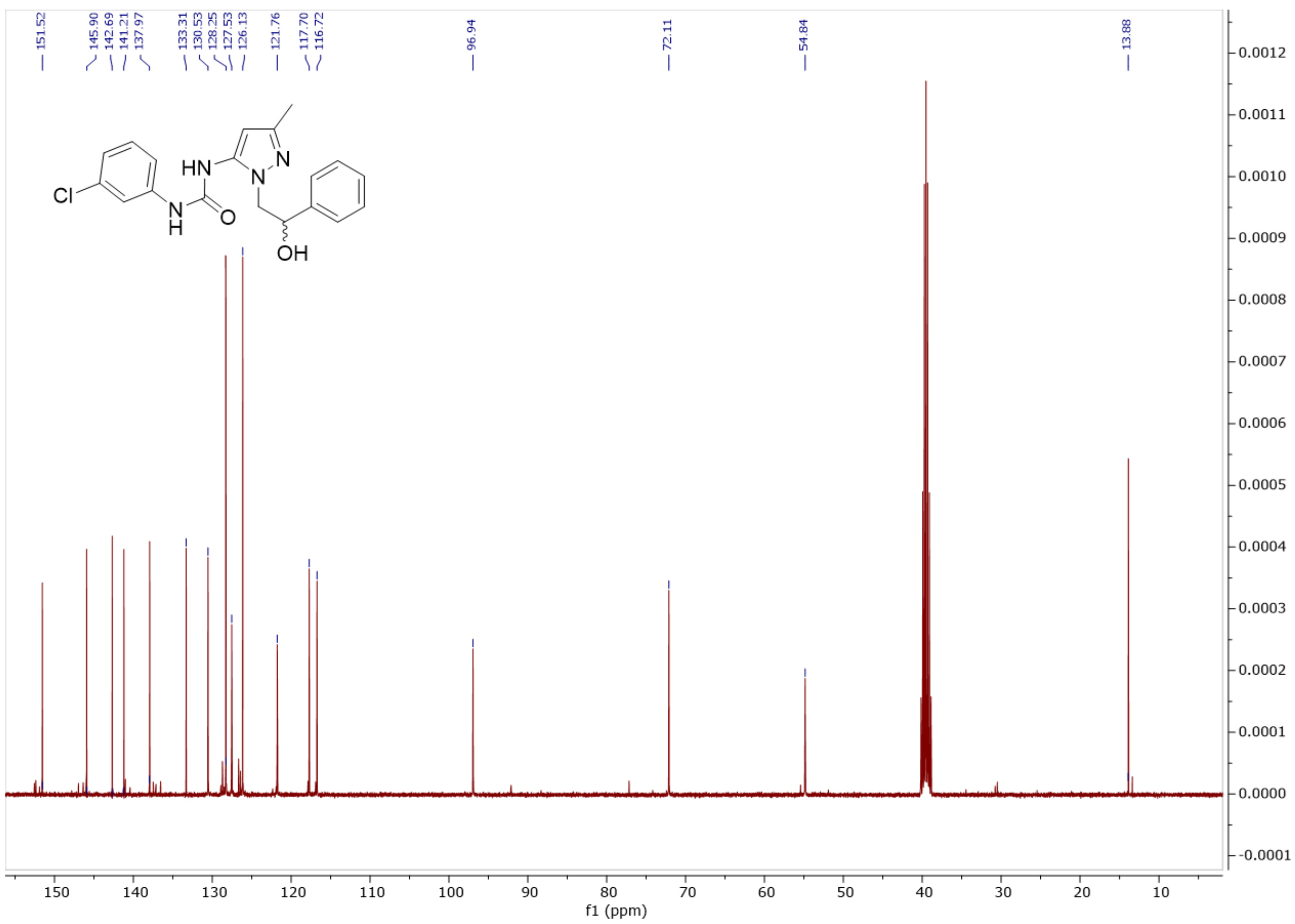

Figure S13. ^{1}H NMR (400 MHz, DMSO-d$_6$) spectrum of compound **1f**

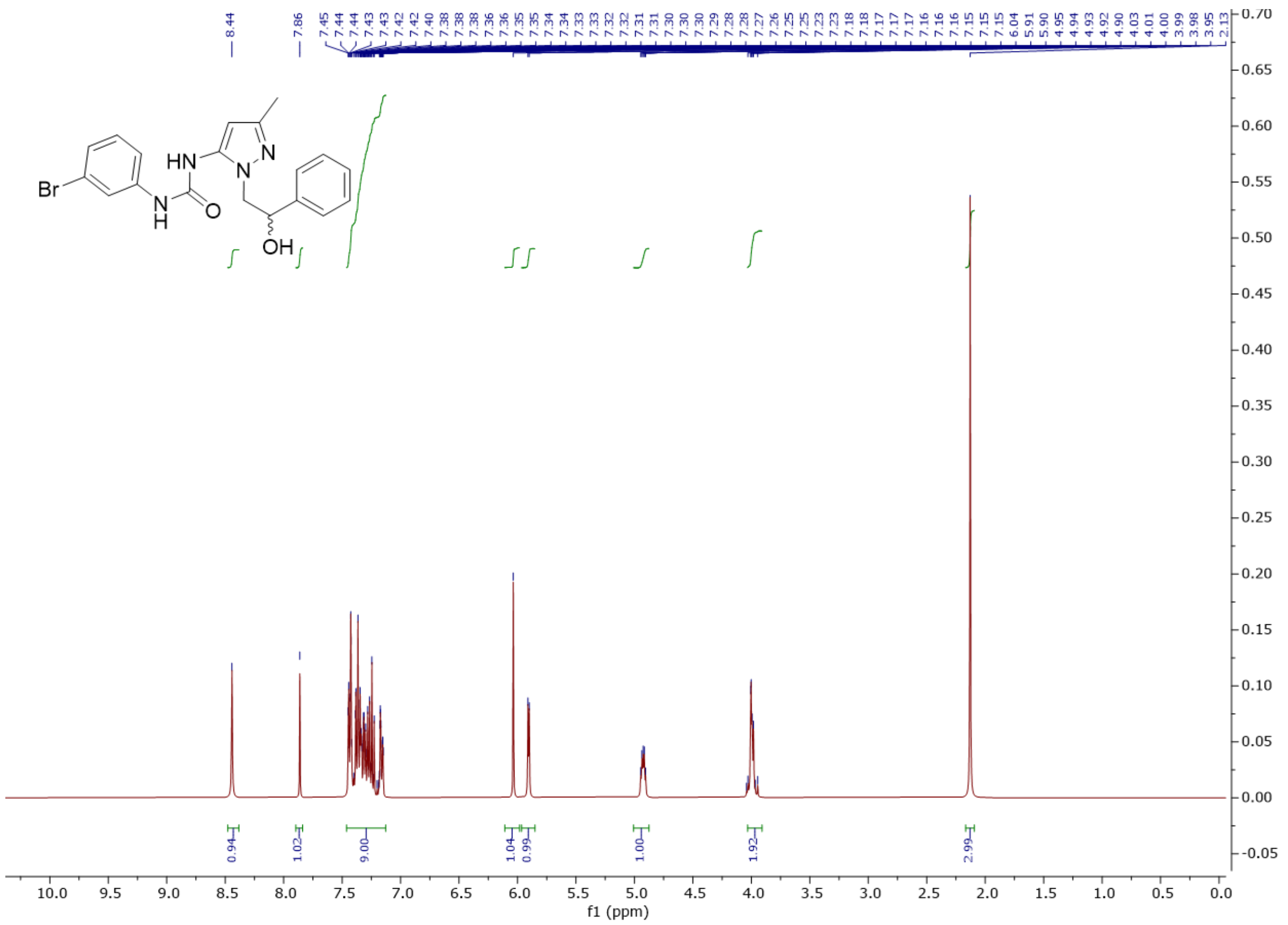

Figure S14. ^{13}C NMR (101 MHz, DMSO-d$_6$) spectrum of compound **1f**

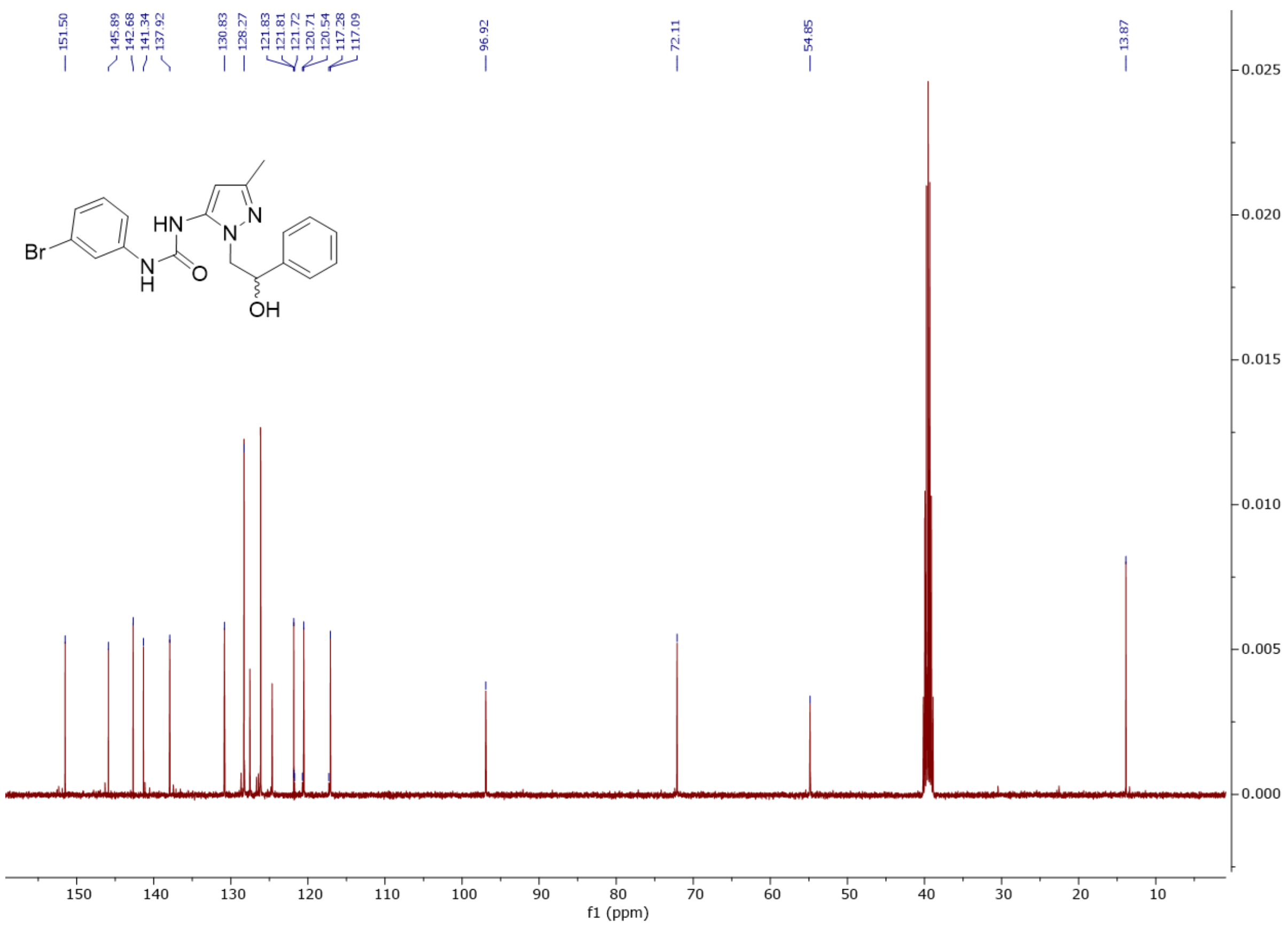

Figure S15. ^{1}H NMR (400 MHz, DMSO-d_6) spectrum of compound **1g**

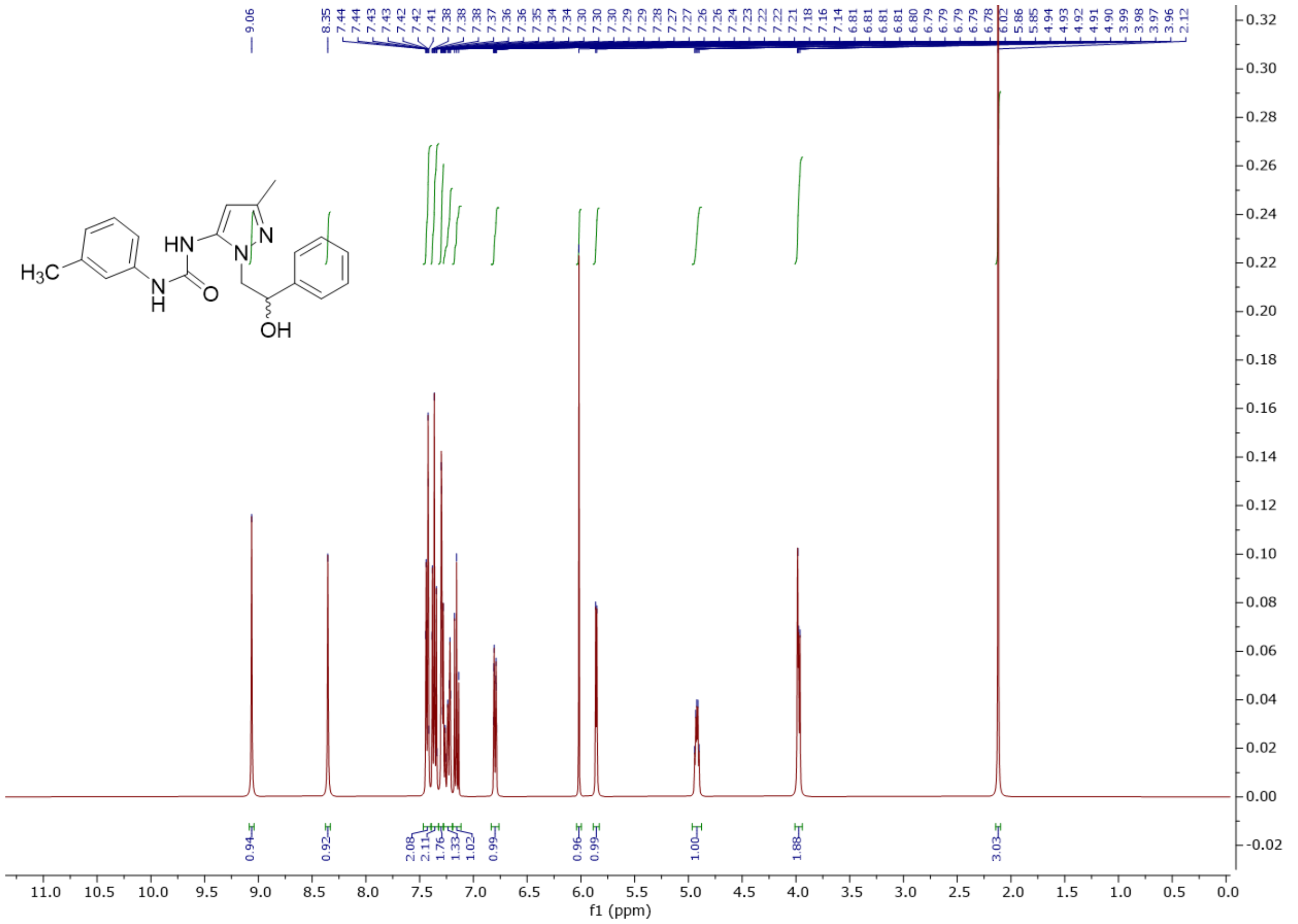

Figure S16. ^{13}C NMR (101 MHz, DMSO-d_6) spectrum of compound **1g**

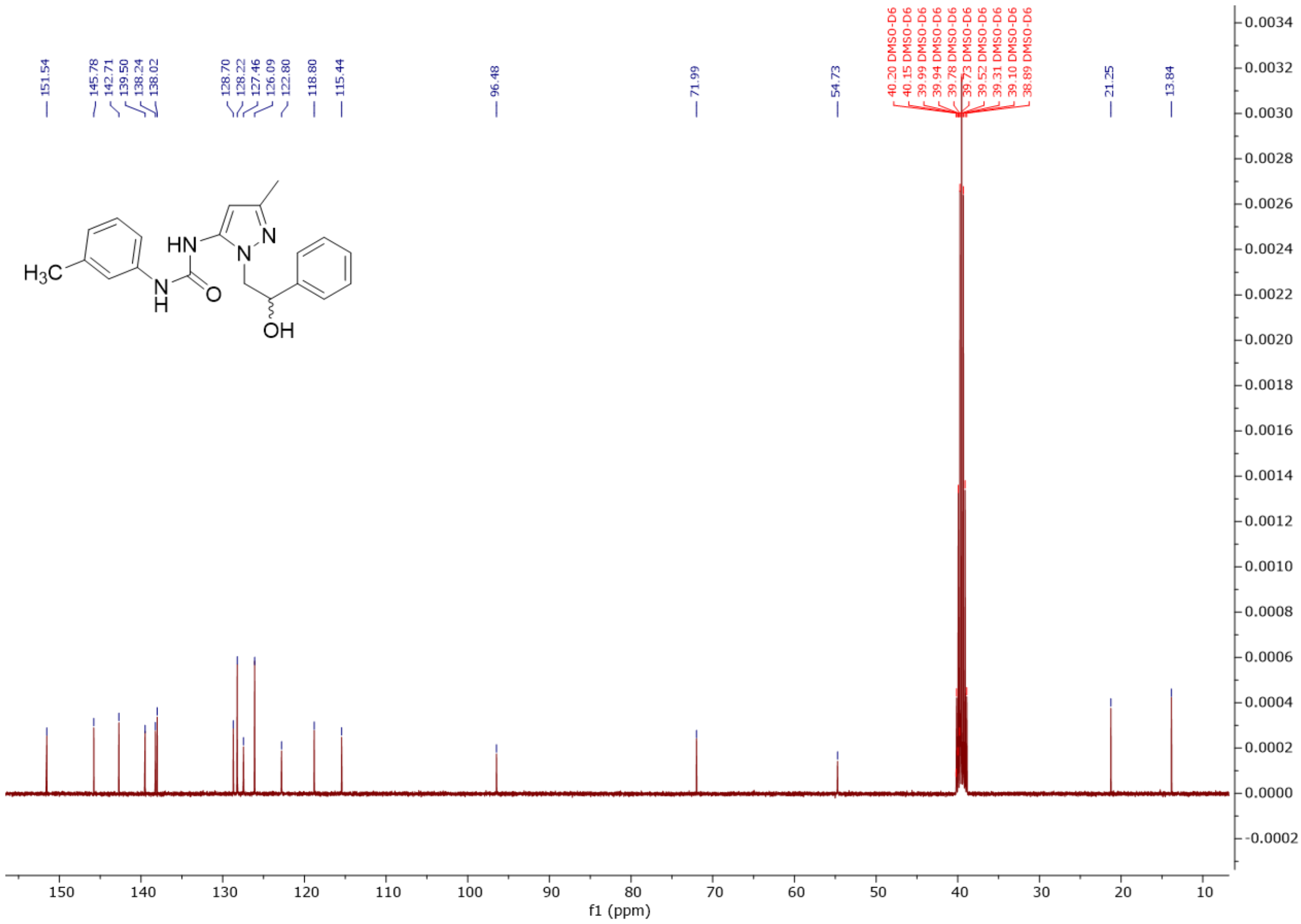

Figure S17. ^{1}H NMR (400 MHz, DMSO-d_6) spectrum of compound **1h**

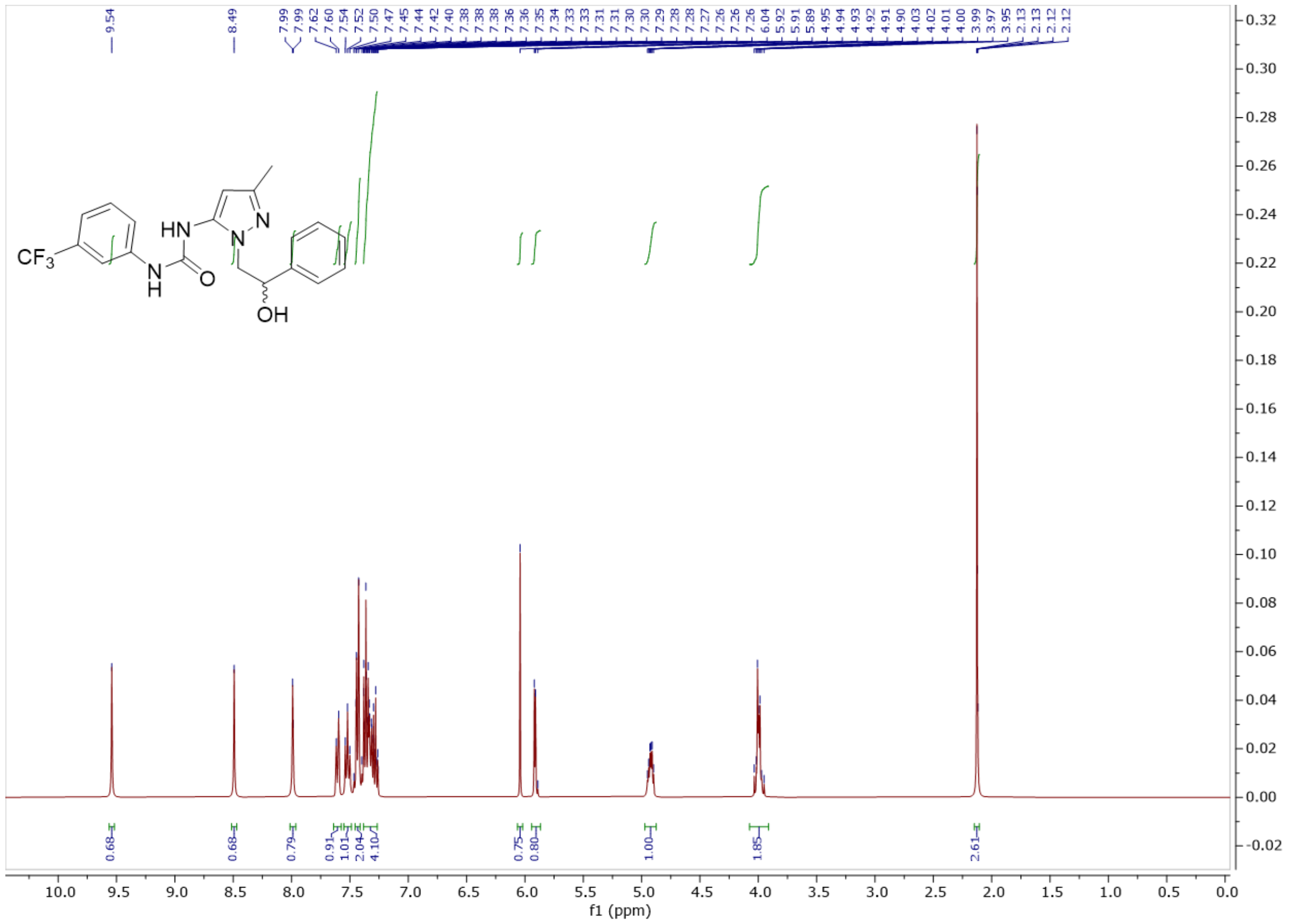

Figure S18. ^{13}C NMR (101 MHz, DMSO-d_6) spectrum of compound **1h**

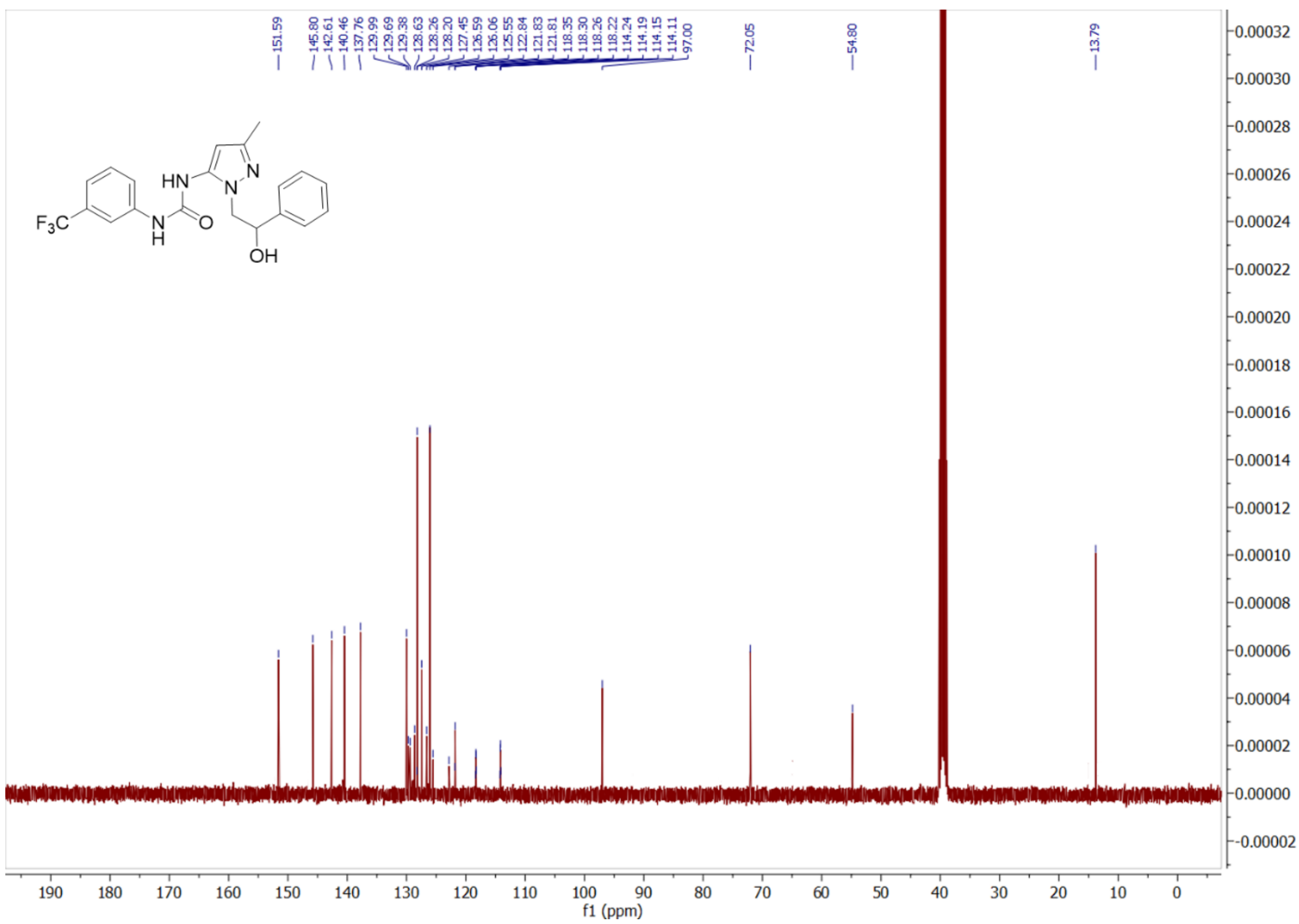

Figure S19. ^{1}H NMR (400 MHz, DMSO-d$_6$) spectrum of compound **2a**

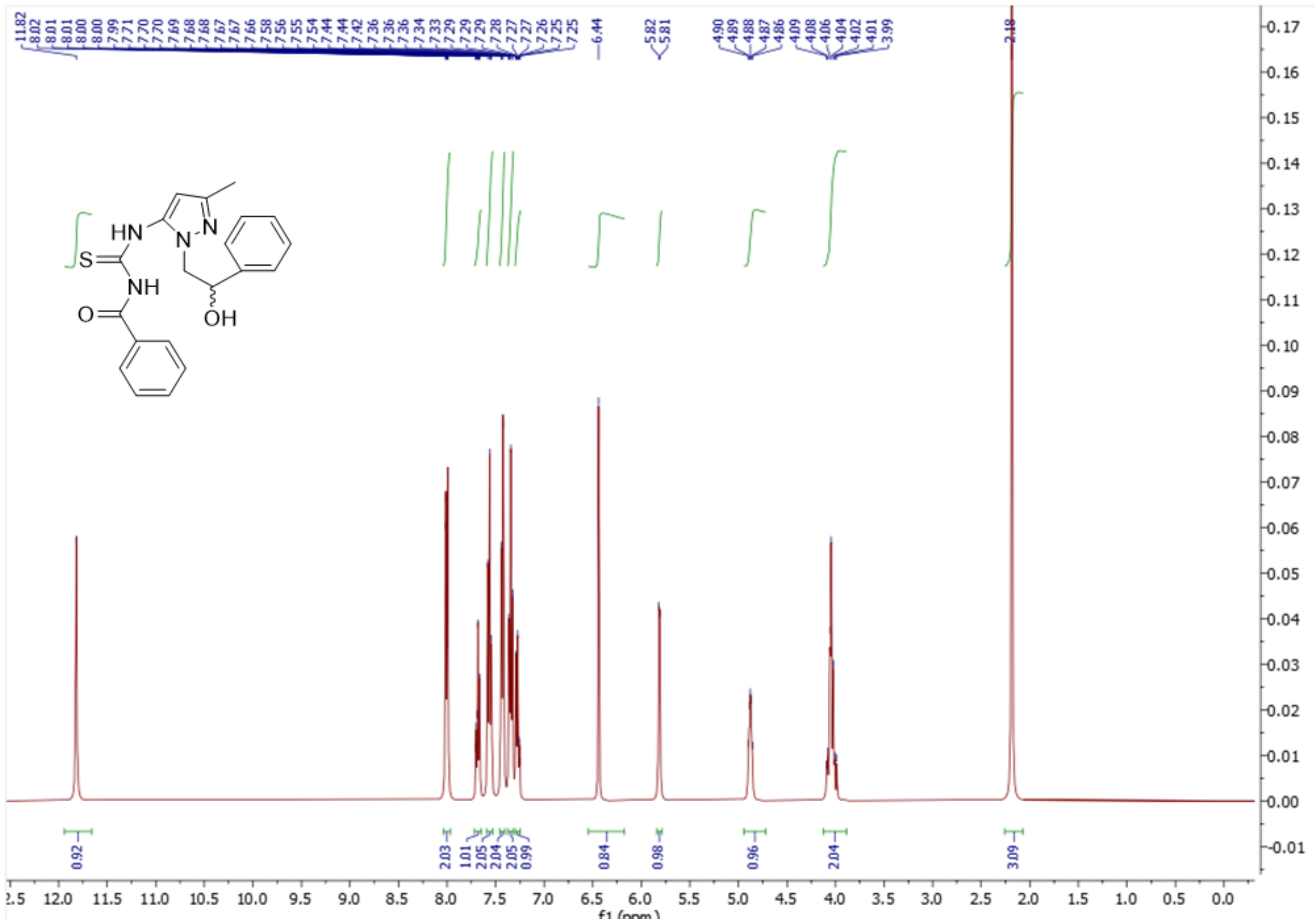

Figure S20. ^{13}C NMR (101 MHz, DMSO-d$_6$) spectrum of compound **2a**

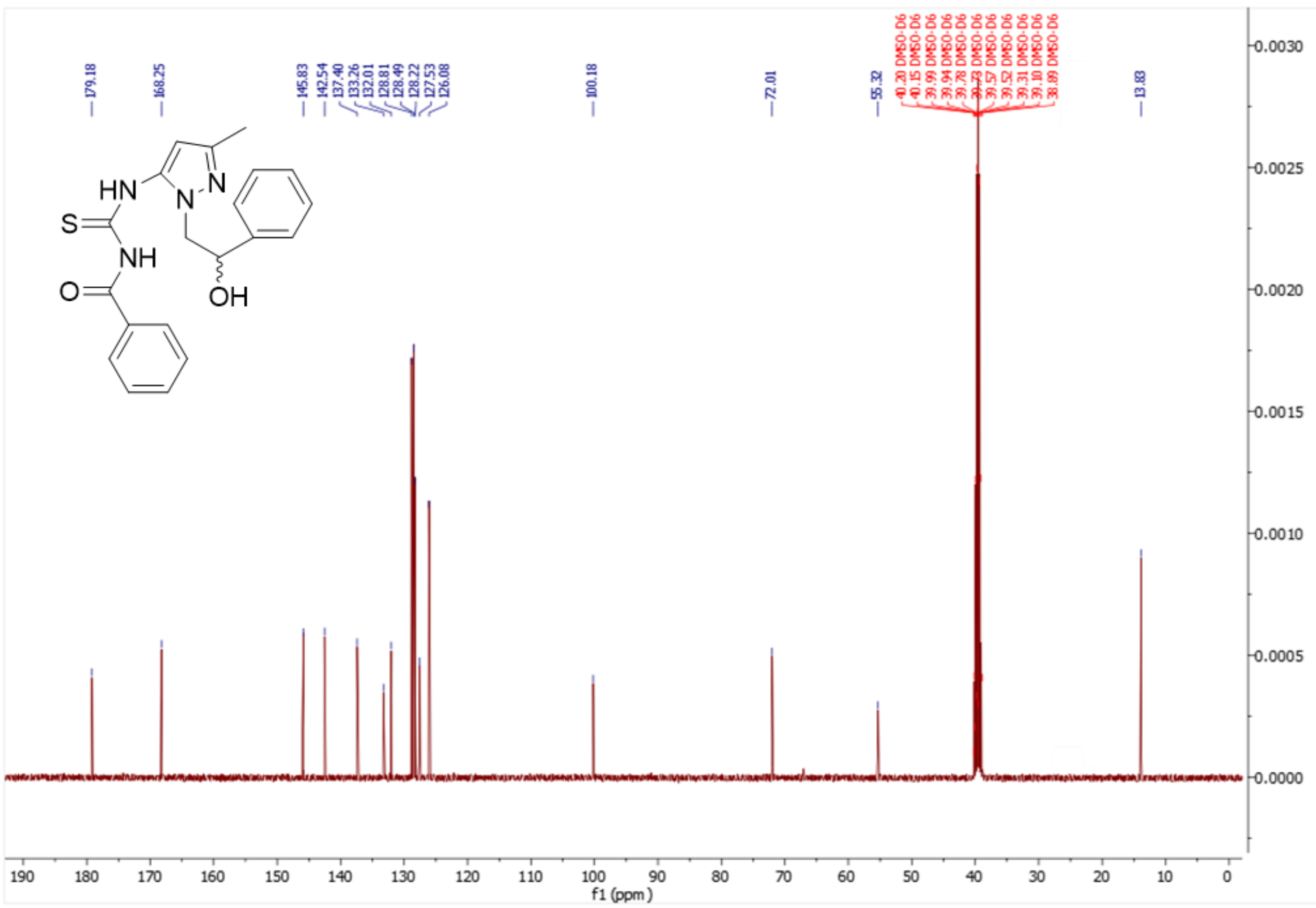

Figure S21. ^{1}H NMR (400 MHz, DMSO-d$_6$) spectrum of compound **2b**

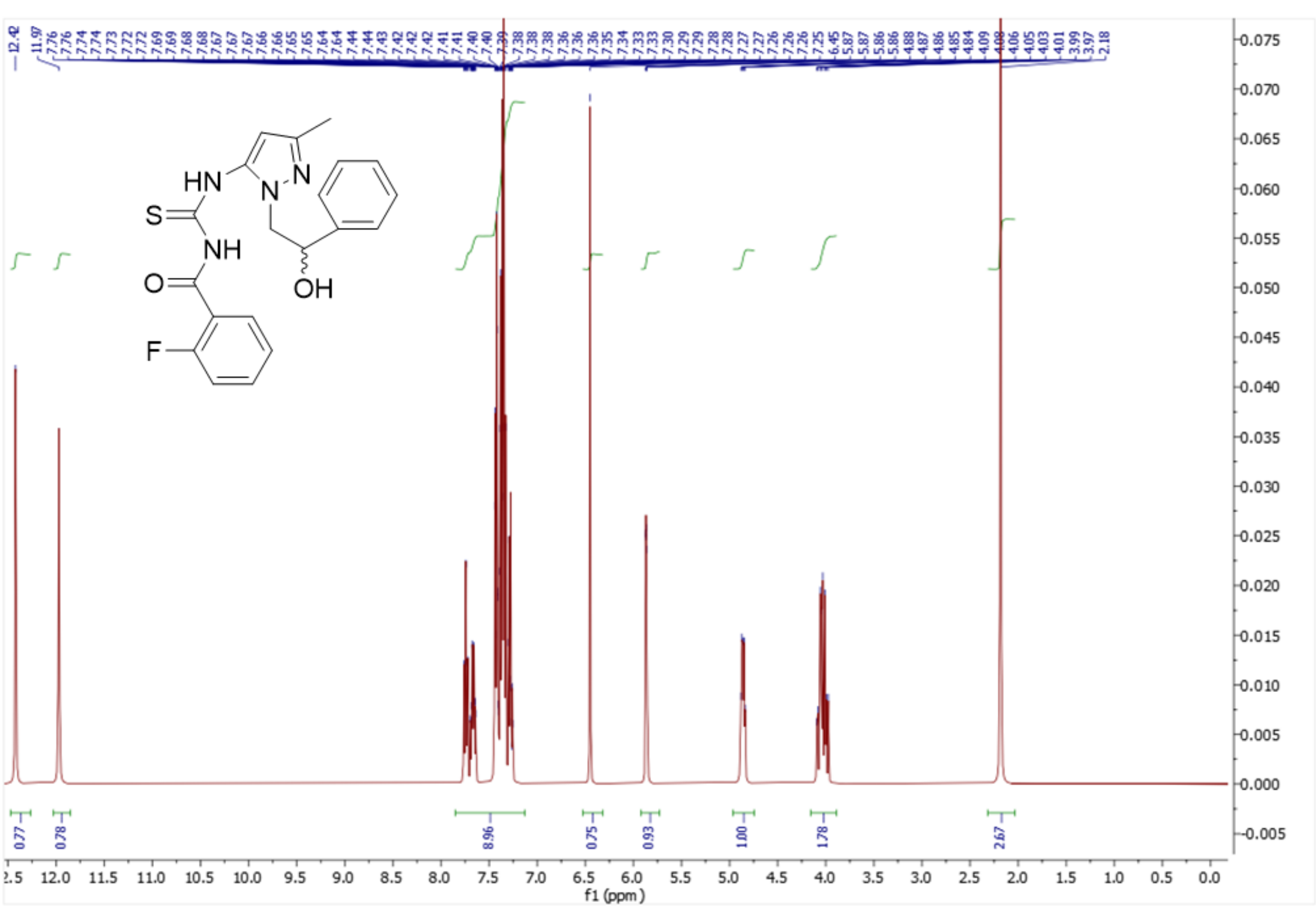

Figure S22. ^{13}C NMR (101 MHz, DMSO-d$_6$) spectrum of compound **2b**

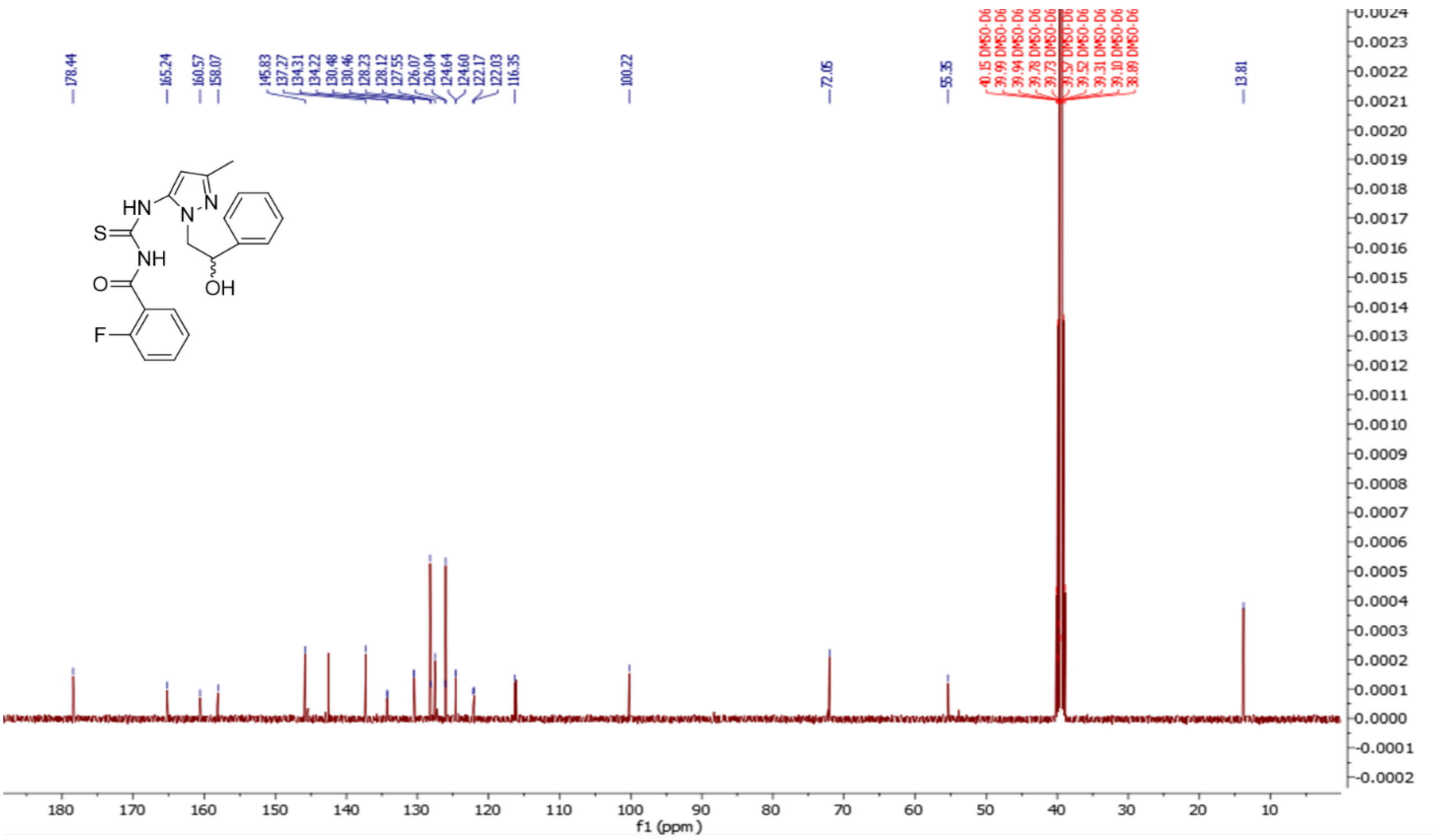

Figure S23. ^{1}H NMR (400 MHz, DMSO-d$_6$) spectrum of compound **2c**

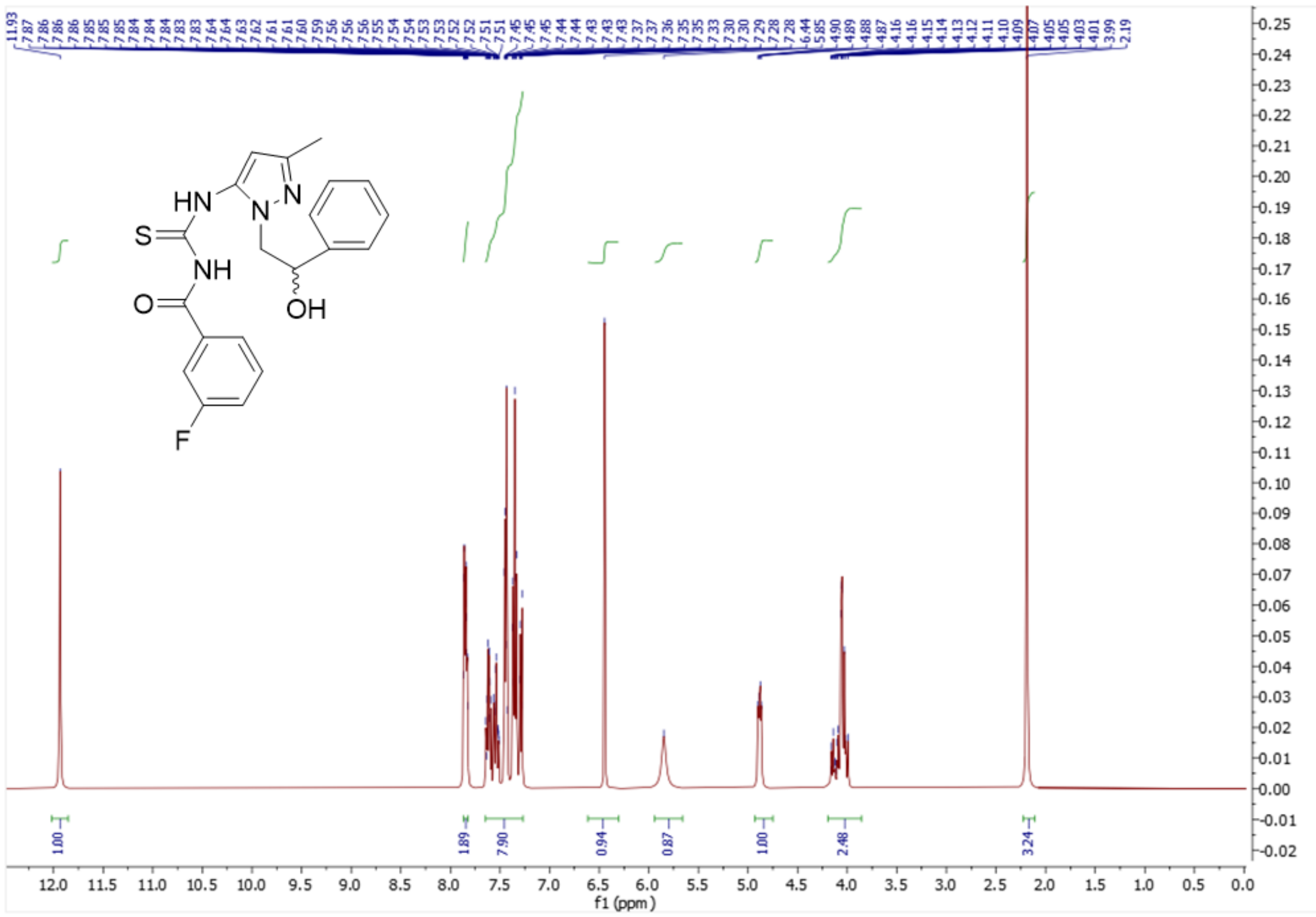

Figure S24. ^{13}C NMR (101 MHz, DMSO-d$_6$) spectrum of compound **2c**

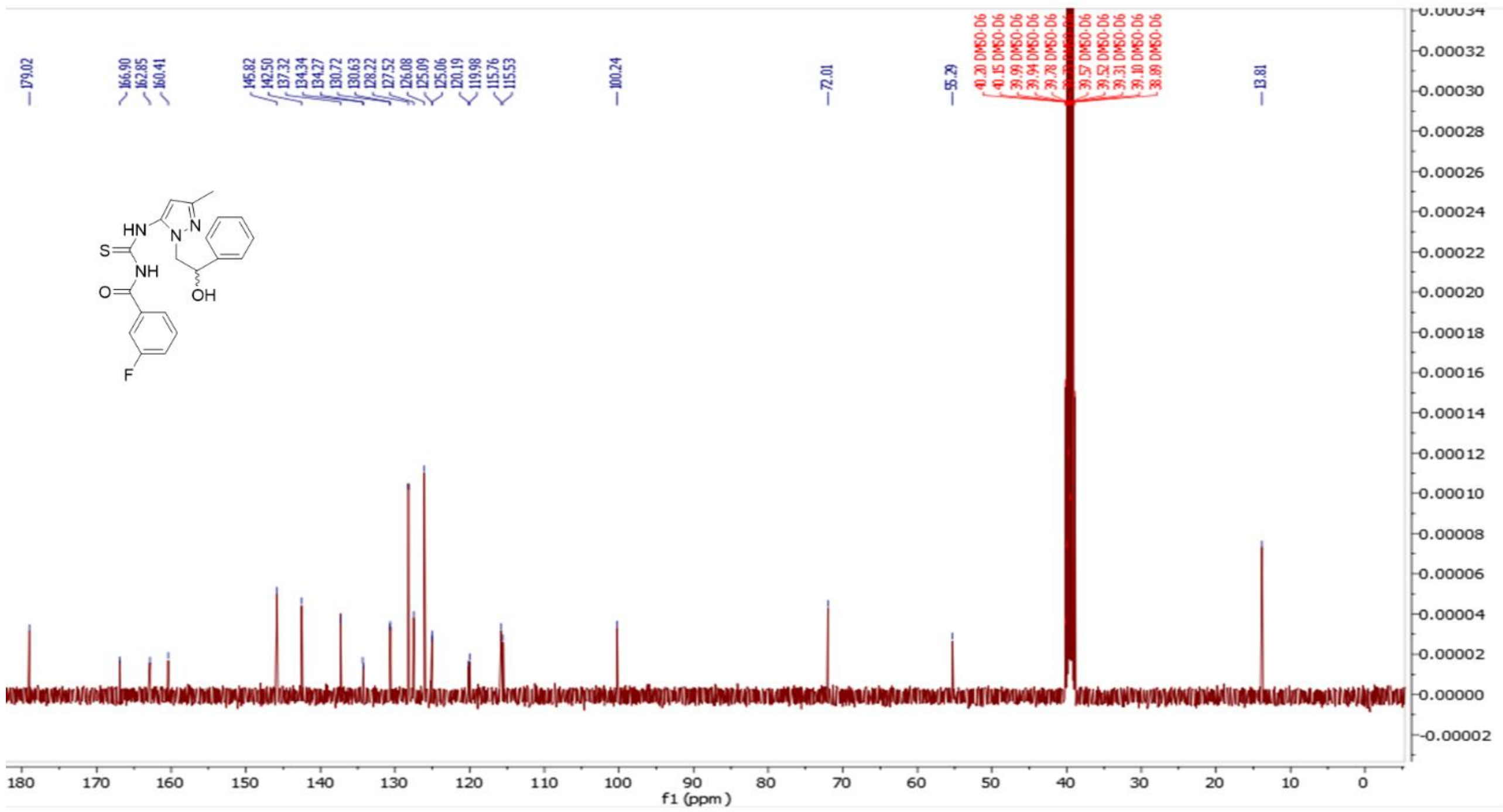

Figure S25. ^{1}H NMR (400 MHz, DMSO-d_6) spectrum of compound **2d**

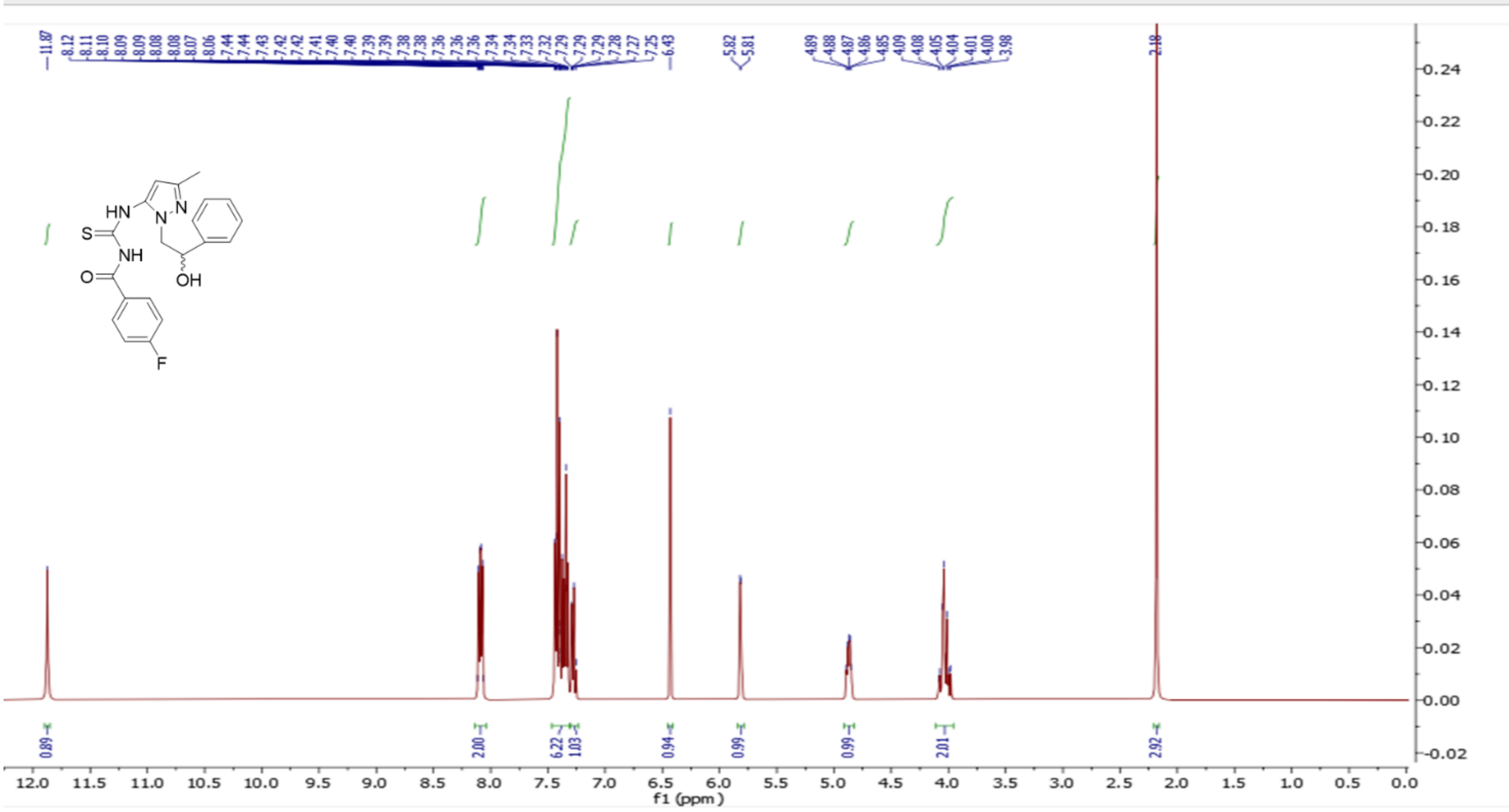

Figure S26. ^{13}C NMR (101 MHz, DMSO-d_6) spectrum of compound **2d**

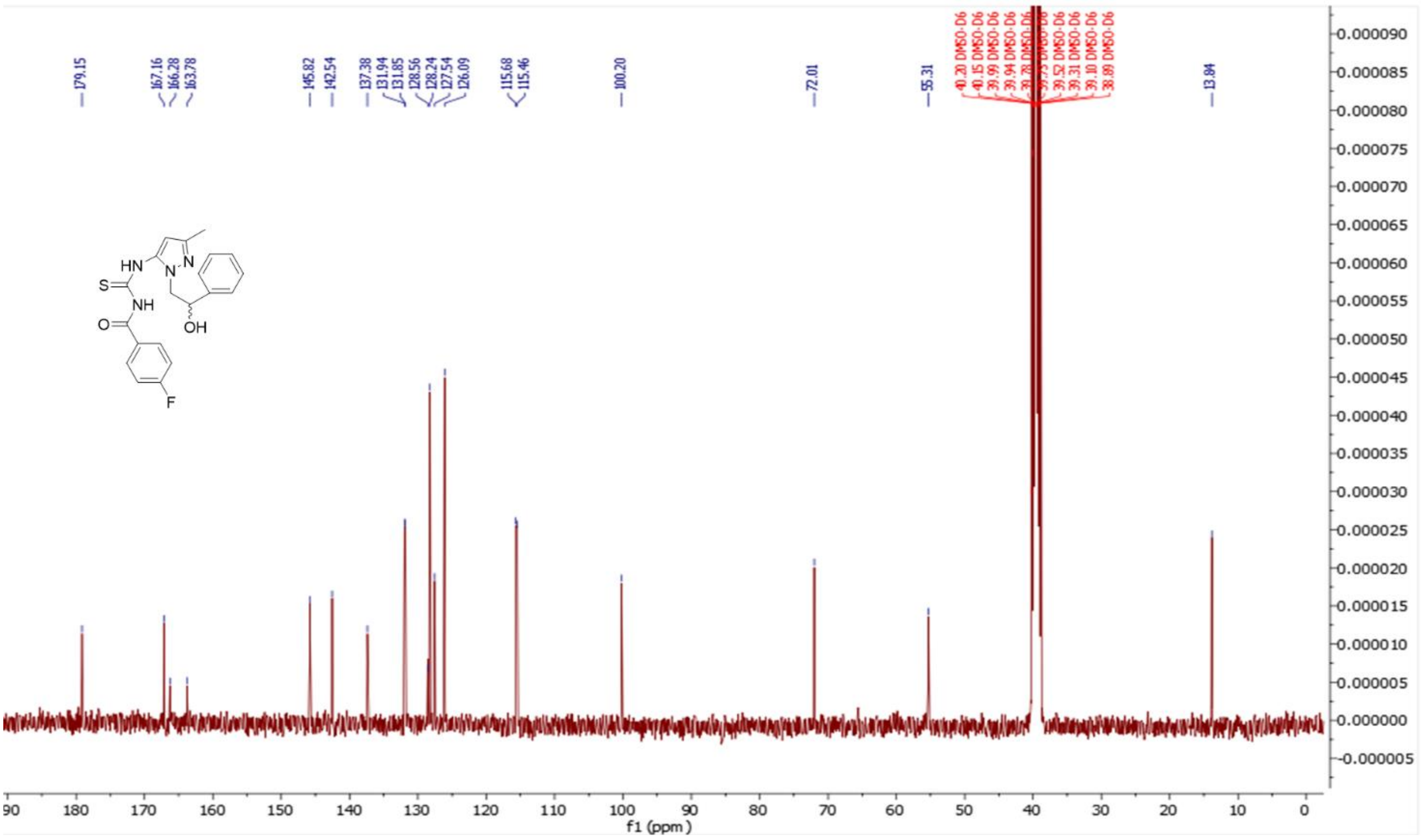

Figure S27. ^{1}H NMR (400 MHz, DMSO-d_6) spectrum of compound **3a**

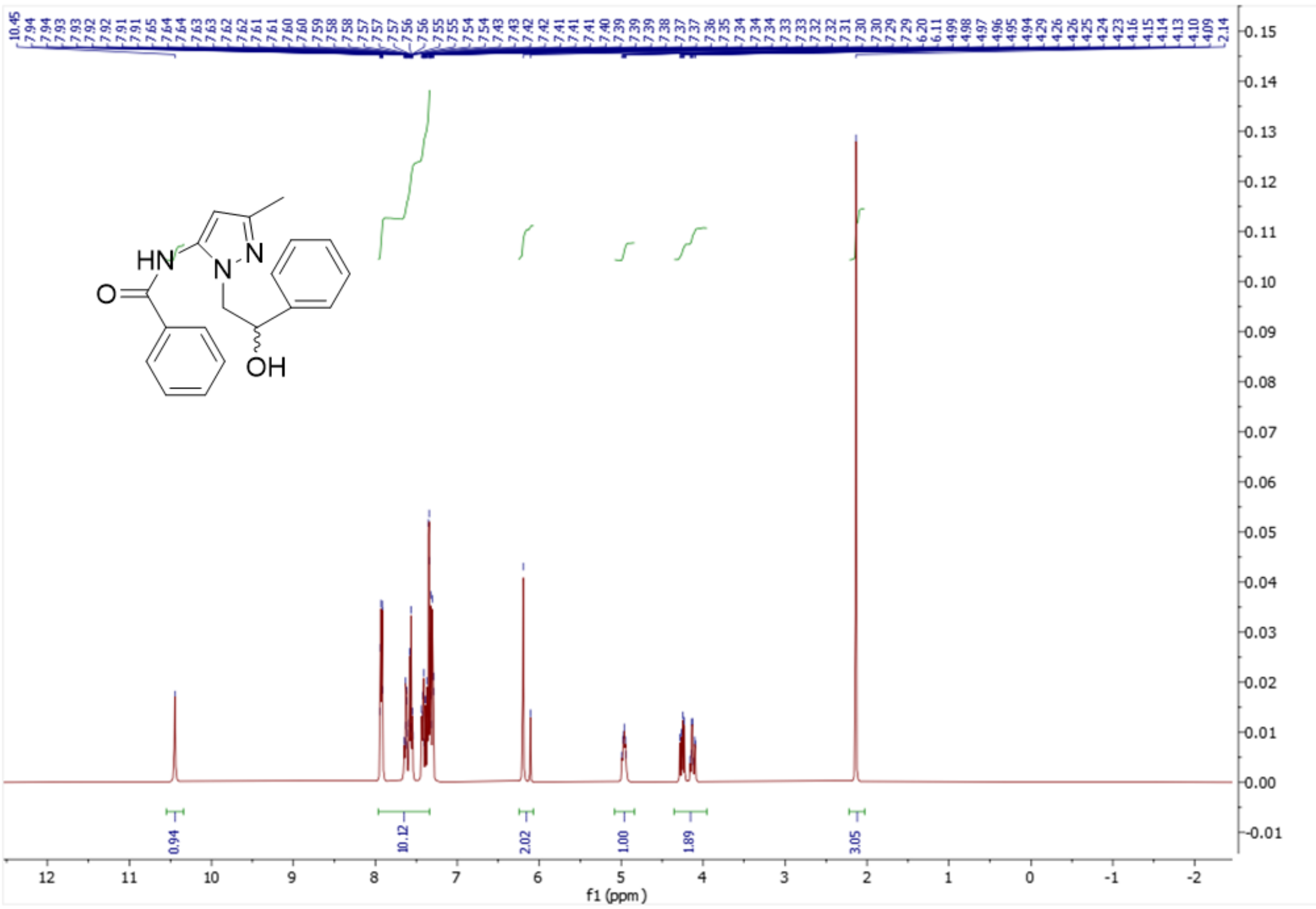

Figure S28. ^{13}C NMR (101 MHz, DMSO-d_6) spectrum of compound **3a**

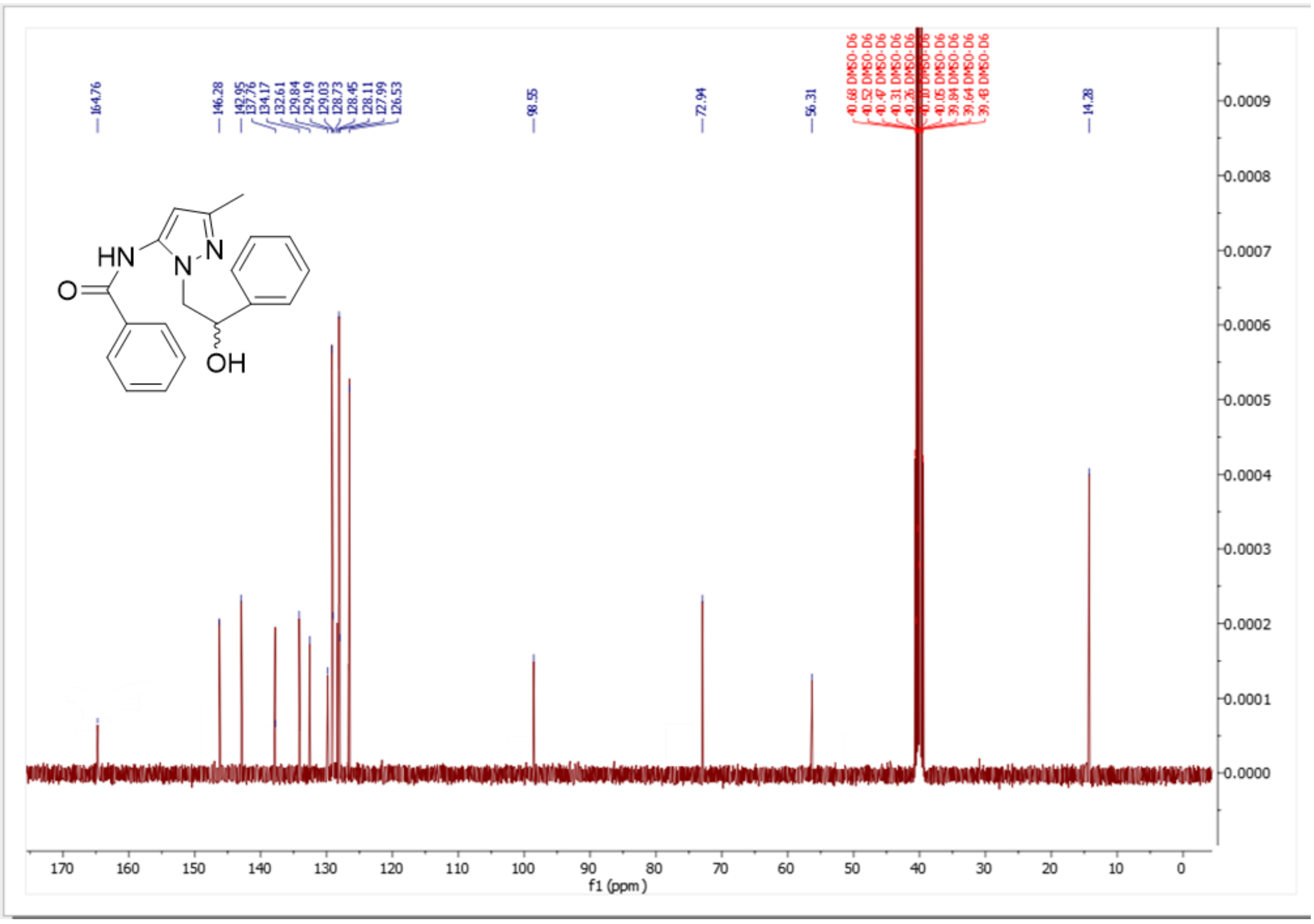

Figure S29. ^{1}H NMR (400 MHz, DMSO-d$_6$) spectrum of compound **3b**

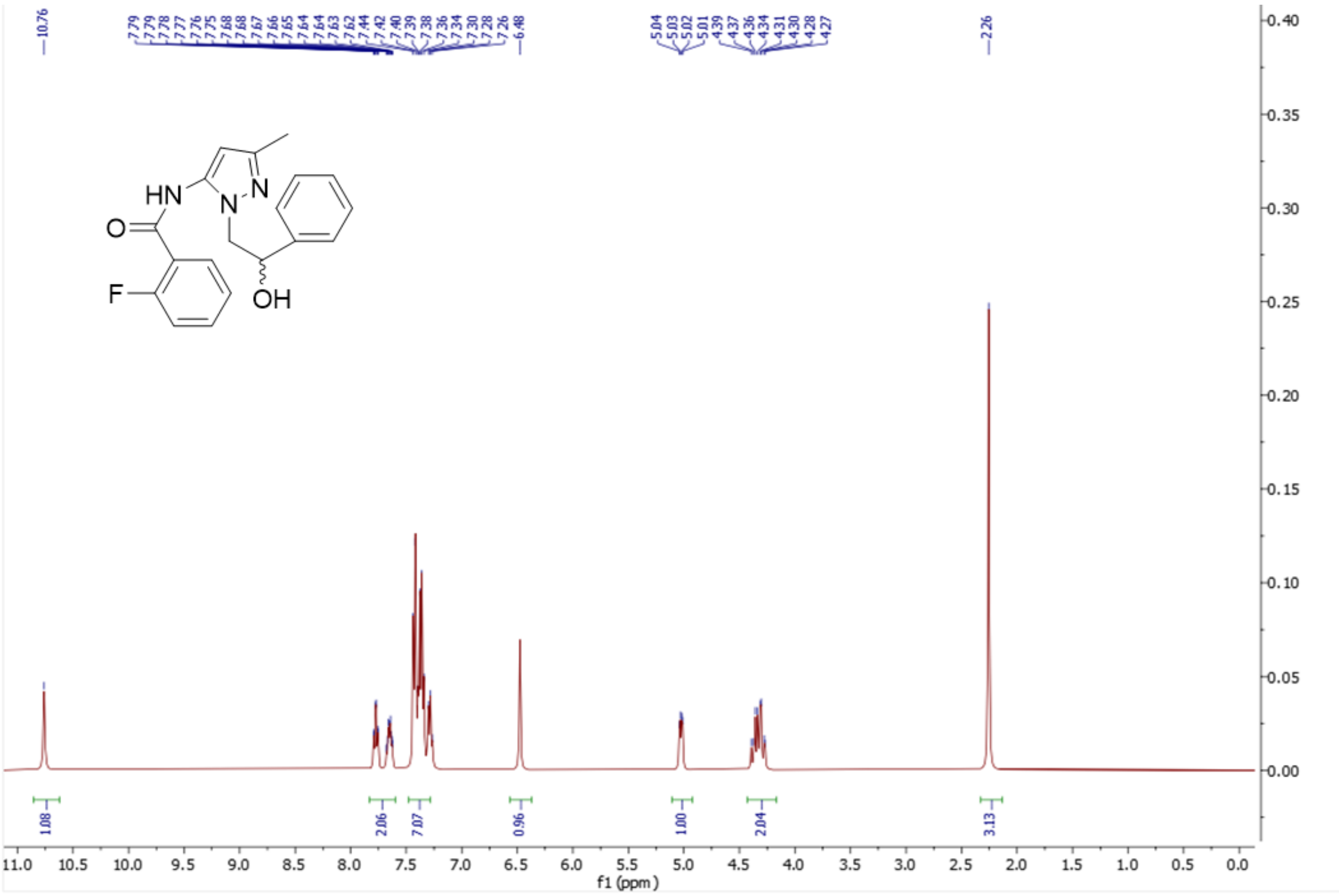

Figure S30. ^{13}C NMR (101 MHz, DMSO-d$_6$) spectrum of compound **3b**

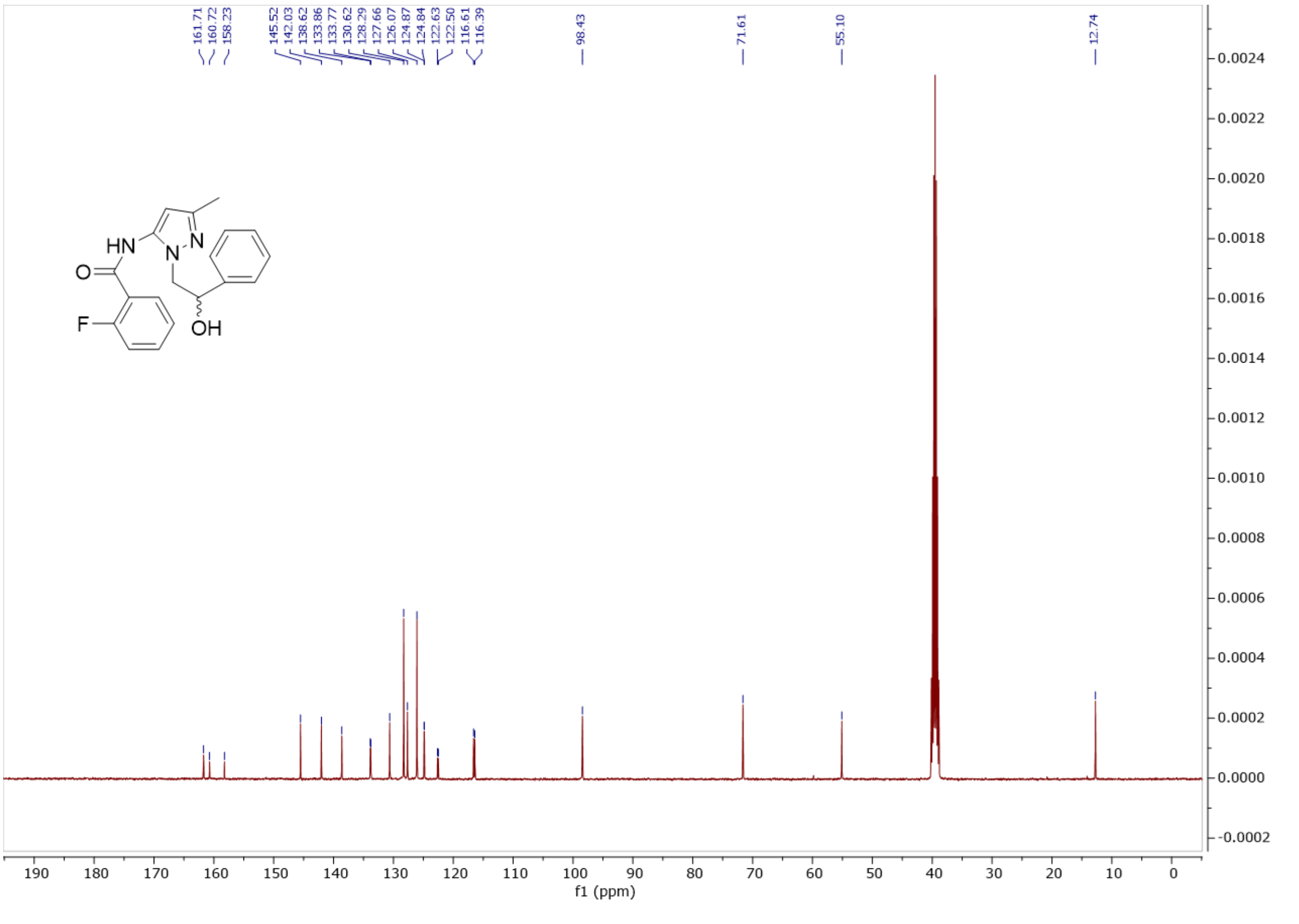

Figure S31. ^{1}H NMR (400 MHz, DMSO-d_6) spectrum of compound **3c**

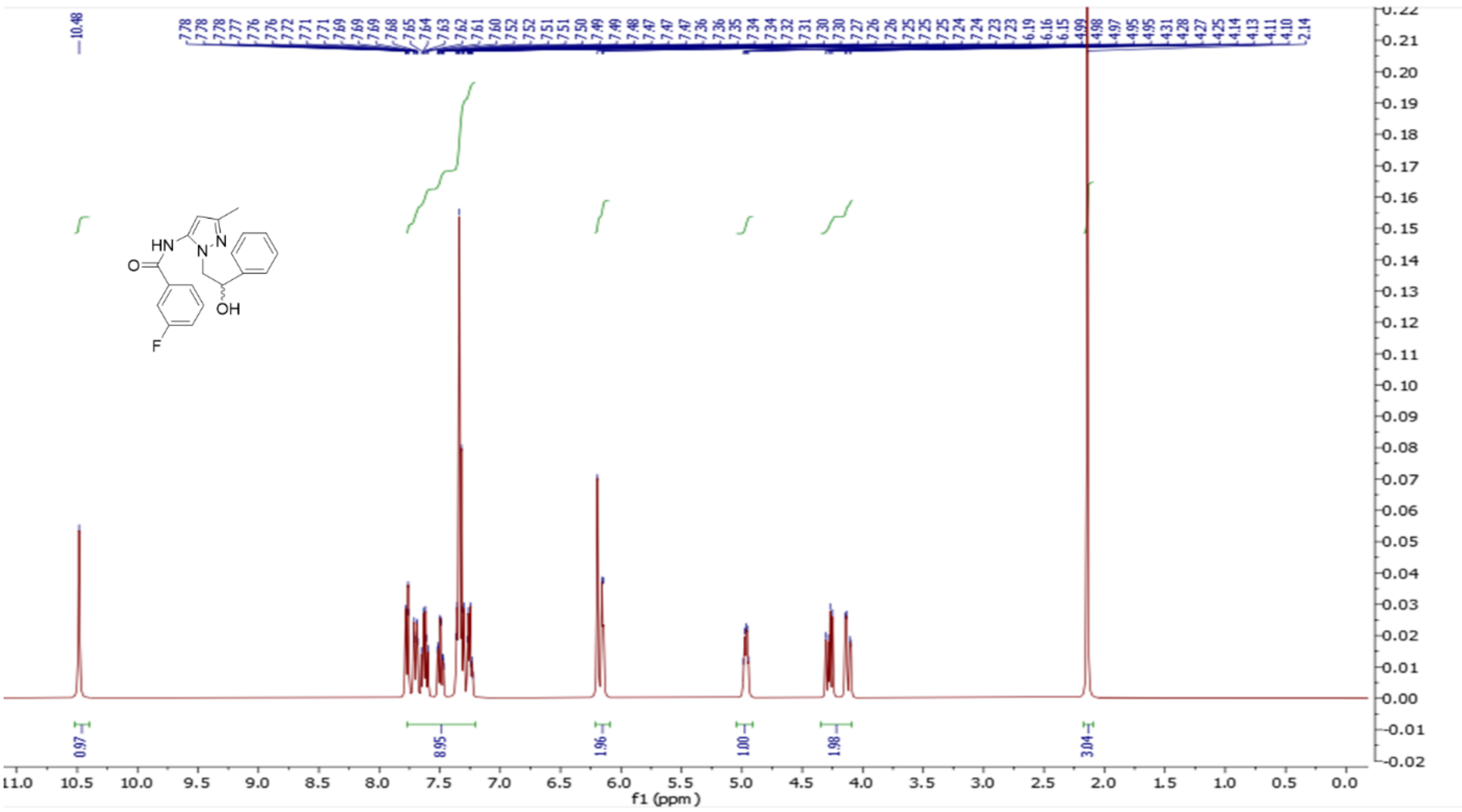

Figure S32. ^{13}C NMR (101 MHz, DMSO-d_6) spectrum of compound **3c**

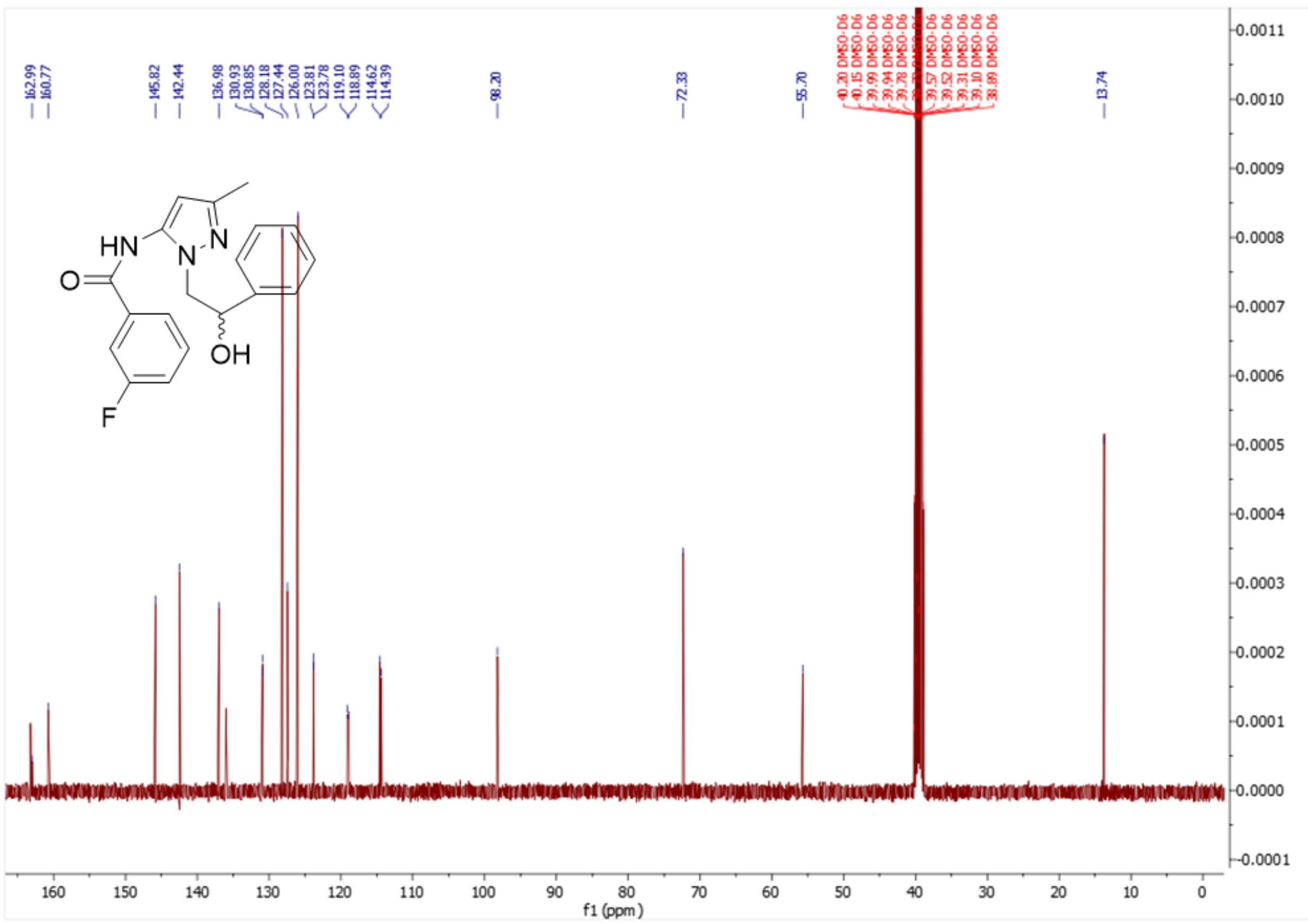

Figure S33. ^{1}H NMR (400 MHz, DMSO-d_6) spectrum of compound **3d**

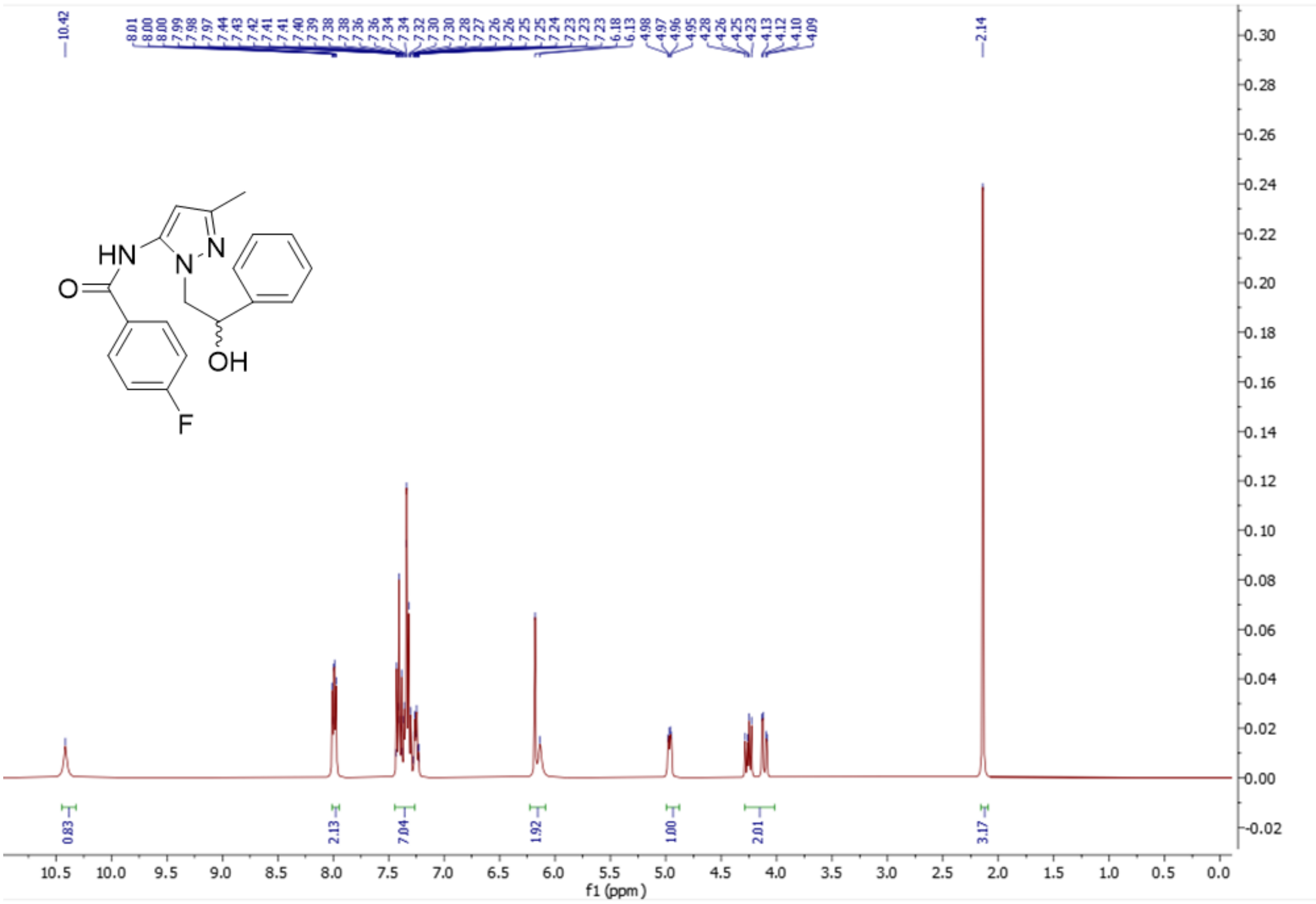

Figure S34. ^{13}C NMR (101 MHz, DMSO-d_6) spectrum of compound **3d**

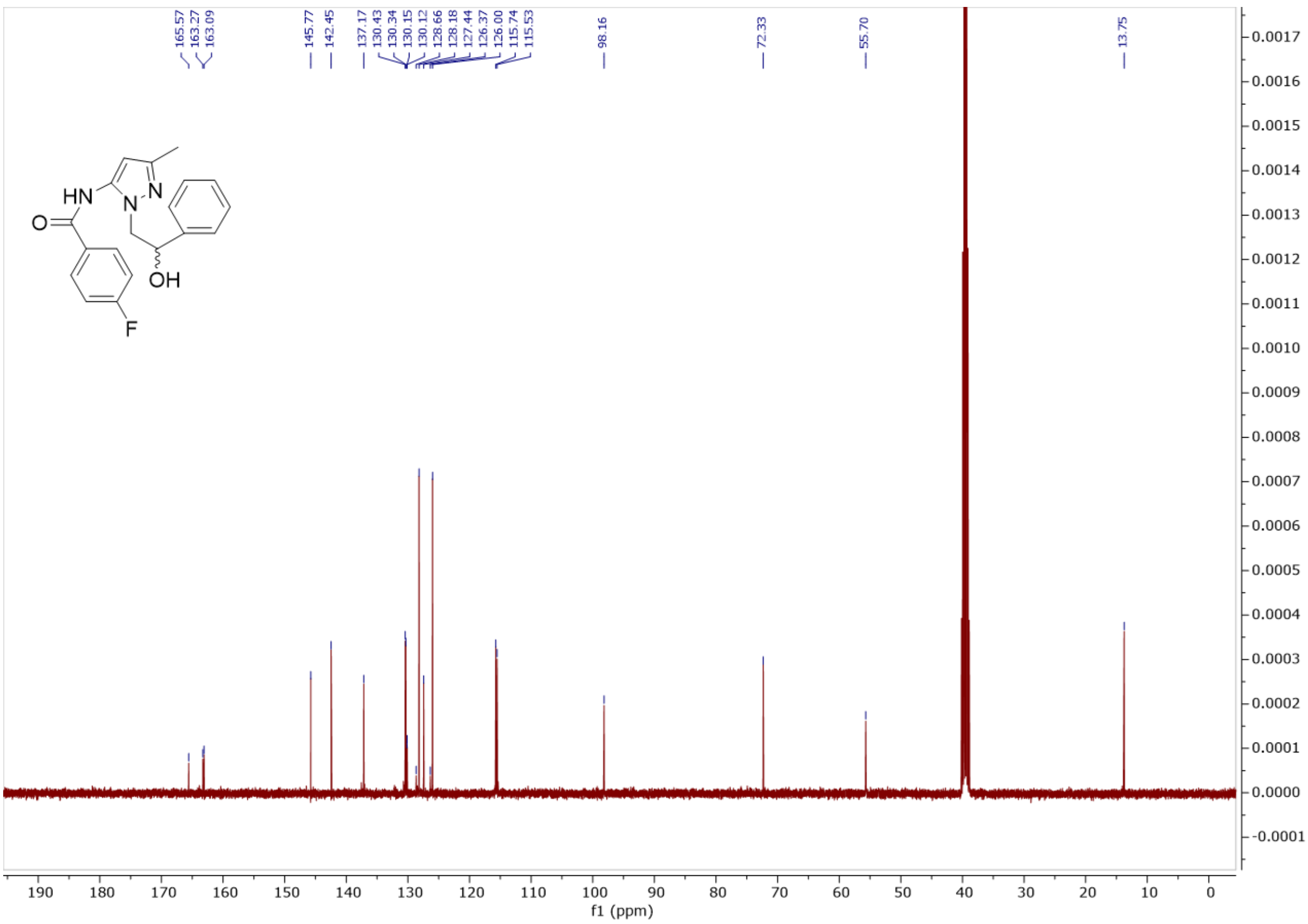

Figure S35. HPLC of compound **1c**

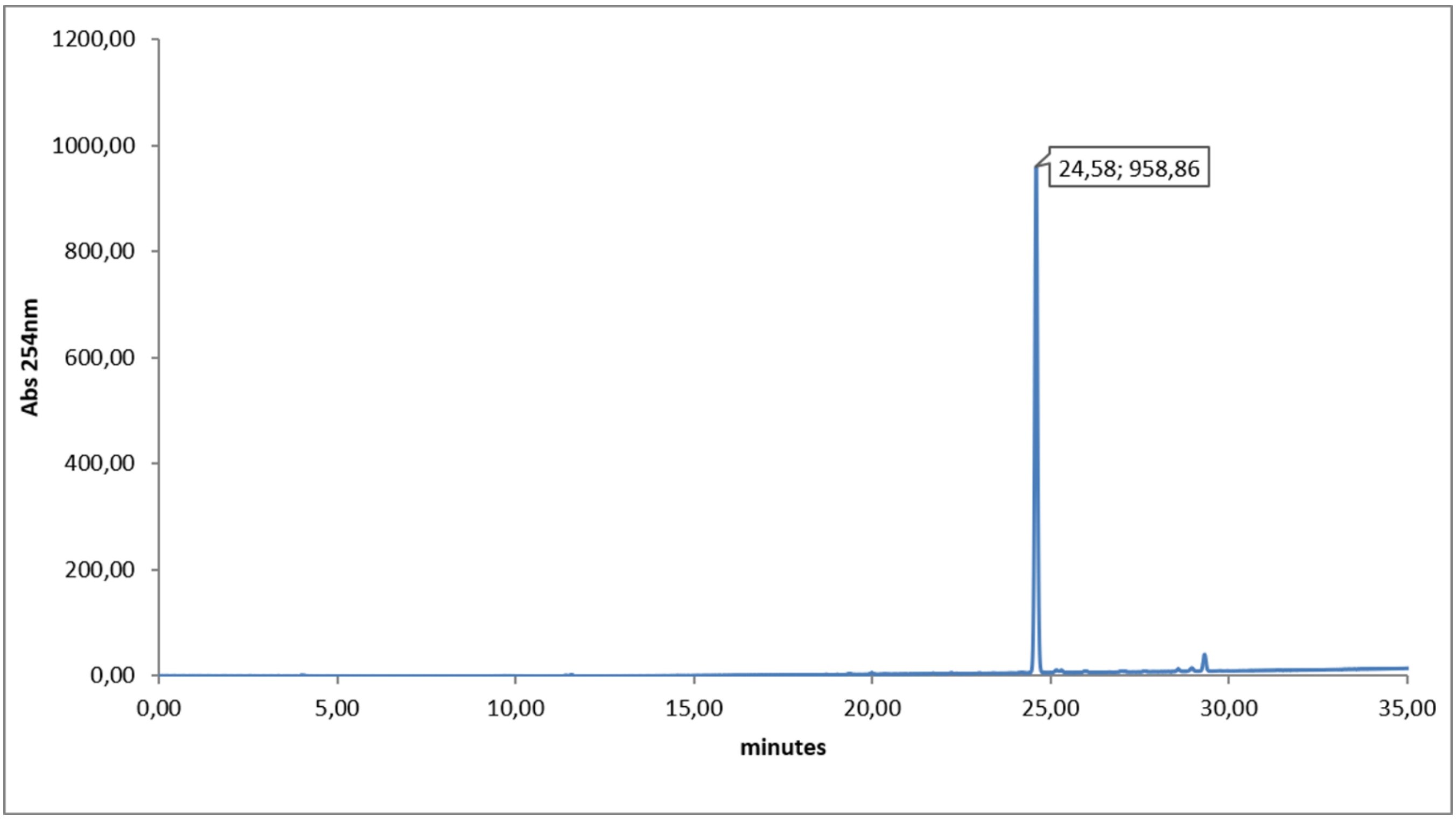

Figure S36. HPLC of compound **1h**

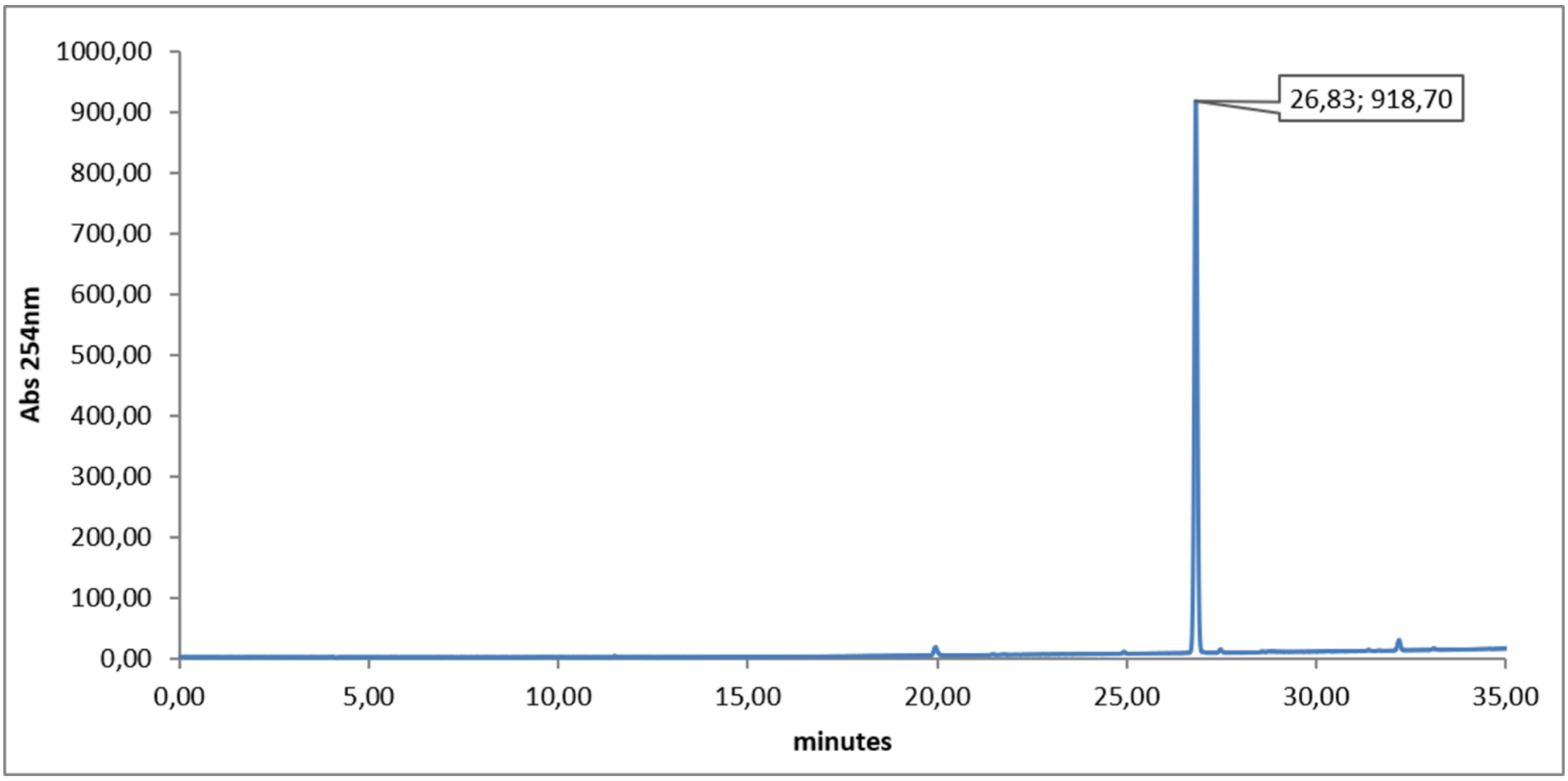

Biological section.

Figure S37. Bacterial growth was quantified by measuring optical density at 570 nm (OD_{570}) after 24 h of incubation. The reference strain *S. aureus* ATCC 29213 and clinical isolates (24, 18, 10, 11, N2, 189, A, B, C, D, 187) were treated with vancomycin at sub-inhibitory concentrations (¼ MIC and ½ MIC) and at the minimum inhibitory concentration (MIC) and compared with untreated controls (CNT). Data are presented as mean ± standard deviation of at least three independent experiments. Statistical analysis was performed using one-way ANOVA test; asterisks indicate statistically significant differences compared to the corresponding control (Dunnett test, $p < 0.0001$).

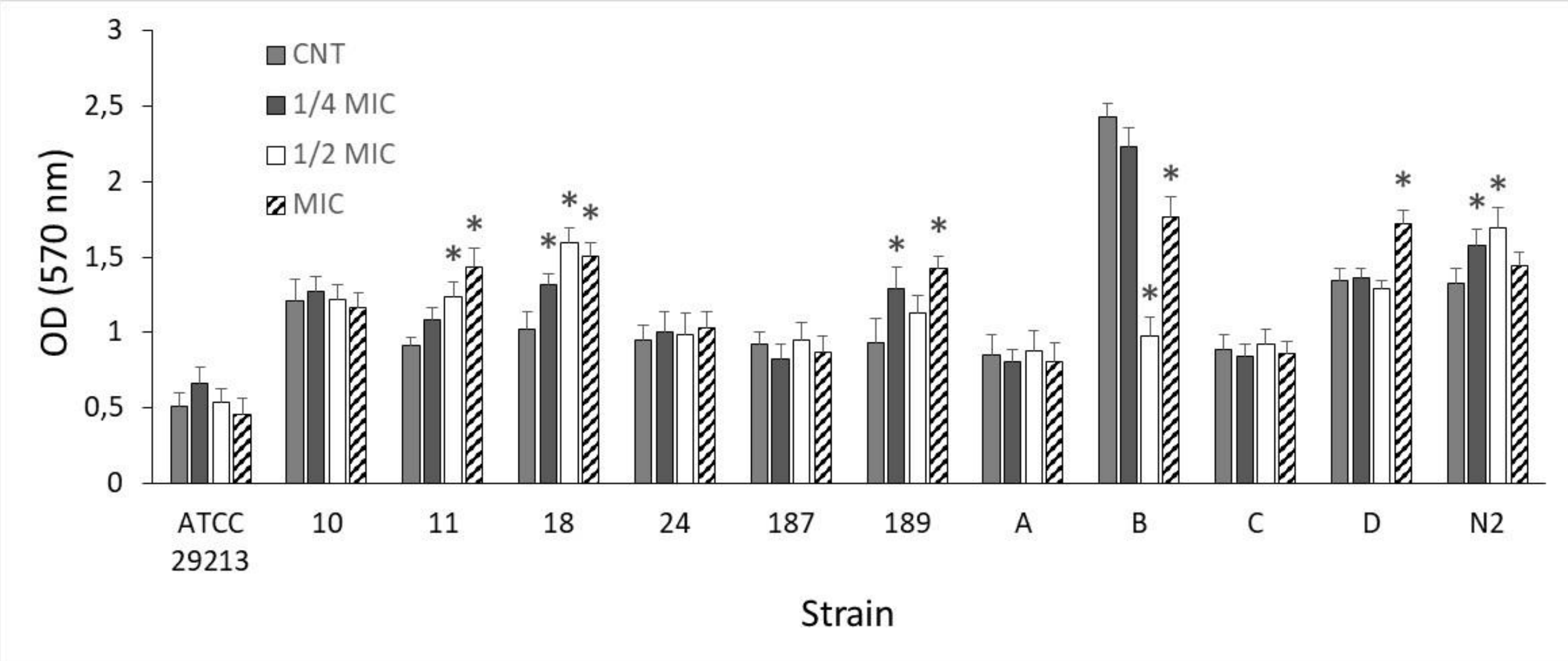

Table S1. Optical density at 570 nm (OD_{570}) values and associated standard deviations measured after 24 h of incubation with vancomycin. Data refers to three independent experiments carried out in triplicate.

	**CTRL**	**1/4 MIC**	**1/2 MIC**	**MIC**
	mean± sd	*mean± sd*	*mean± sd*	*mean± sd*
S. Aureus 24	0.95±0.10	1.00±0.13	0.99±0.14	1.03±0.11
S. Aureus ATCC 29213	0.51±0.08	0.66±0.10	0.54±0.09	0.45±0.11
S. Aureus 18	1.02±0.12	1.31±0.07	1.59±0.10	1.50±0.09
S. Aureus 10	1.21±0.14	1.27±0.10	1.22±0.10	1.16±0.10
S. Aureus 11	0.91±0.06	1.08±0.08	1.23±0.10	1.43±0.13
S. Aureus N2	1.33±0.10	1.58±0.10	1.70±0.13	1.44±0.09
S. Aureus 189	0.93±0.16	1.29±0.14	1.13±0.11	1.42±0.09
S. Aureus A	0.85±0.13	0.80±0.09	0.88±0.14	0.81±0.12
S. Aureus B	2.43±0.09	2.23±0.13	0.97±0.13	1.76±0.13
S. Aureus C	0.89±0.10	0.84±0.08	0.92±0.10	0.85±0.09
S. Aureus D	1.34±0.08	1.36±0.07	1.29±0.05	1.72±0.09
S. Aureus 187	0.92±0.07	0.82±0.10	0.95±0.11	0.87±0.10

Table S2. MIC values of vancomycin against selected *S. aureus* strains expressed in μg/mL. Experiments were carried out in triplicate, * indicates resistance towards methicillin.

**Strains**	**vancomycin MIC (μg/mL)**
S. Aureus A *	0.5
S. Aureus B *	0.5
S. Aureus C *	0.25
S. Aureus D *	0.5
S. Aureus 10 *	0.5
S. Aureus 11 *	0.5
S. Aureus 18 *	0.5
S. Aureus 24 *	0.5
S. Aureus ATCC29213	0.5
S. Aureus N_2	0.5
S. Aureus 187*	0.5
S. Aureus 189 *	0.5

Pharmacokinetics and drug-likeness properties of 1c and 1h (SwissADME)

Table S3. Predicted properties of **1c** and **1h**

**Physicochemical Property**	**1c**	**1h**
MW (g/mol)	353.39	403.40
Fraction Csp^3	0.20	0.24
Rotatable bonds	7	8
H bond acceptors	4	6
H bond donors	2	2
TPSA[a] ($Å^2$)	67.15 $Å^2$	67.15 $Å^2$
**Lipophilicity**		
LogP[b]	2.32	3.10
**Water solubility**		
Solubility (mg/mL)[c]	1.08 x 10^{-2} mg/mL	2.55 x 10^{-2} mg/mL
Solubility	soluble	Moderately soluble
**Pharmacokinetics**		
GI absorption	high	high
BBB permeant	yes	yes
Pgp substrate	yes	yes
CYP1A2 inhibitor	no	no
CYP2C19 inhibitor	yes	yes
CYP2C9 inhibitor	no	yes
CYP2D6 inhibitor	yes	yes
CYP3A4 inhibitor	yes	yes
**Drug-likeness**		
Lipinski violations	0	0
**Medicinal Chemistry**		
PAINS alerts	0	0
Brenk alerts	0	0

[a]Topological Polar Surface Area.
[b] Predicted according to XLOGP3 program.
[c] Values predicted by ESOL method
